# Scalable watermarking for identifying large language model outputs

**DOI:** 10.1038/s41586-024-08025-4

**Published:** 2024-10-23

**Authors:** Sumanth Dathathri, Abigail See, Sumedh Ghaisas, Po-Sen Huang, Rob McAdam, Johannes Welbl, Vandana Bachani, Alex Kaskasoli, Robert Stanforth, Tatiana Matejovicova, Jamie Hayes, Nidhi Vyas, Majd Al Merey, Jonah Brown-Cohen, Rudy Bunel, Borja Balle, Taylan Cemgil, Zahra Ahmed, Kitty Stacpoole, Ilia Shumailov, Ciprian Baetu, Sven Gowal, Demis Hassabis, Pushmeet Kohli

**Affiliations:** 1 Google DeepMind, London, UK; 2 Google, Mountain View, CA USA

**Keywords:** Computer science, Information technology

## Abstract

Large language models (LLMs) have enabled the generation of high-quality synthetic text, often indistinguishable from human-written content, at a scale that can markedly affect the nature of the information ecosystem^1–3^. Watermarking can help identify synthetic text and limit accidental or deliberate misuse^4^, but has not been adopted in production systems owing to stringent quality, detectability and computational efficiency requirements. Here we describe SynthID-Text, a production-ready text watermarking scheme that preserves text quality and enables high detection accuracy, with minimal latency overhead. SynthID-Text does not affect LLM training and modifies only the sampling procedure; watermark detection is computationally efficient, without using the underlying LLM. To enable watermarking at scale, we develop an algorithm integrating watermarking with speculative sampling, an efficiency technique frequently used in production systems^5^. Evaluations across multiple LLMs empirically show that SynthID-Text provides improved detectability over comparable methods, and standard benchmarks and human side-by-side ratings indicate no change in LLM capabilities. To demonstrate the feasibility of watermarking in large-scale-production systems, we conducted a live experiment that assessed feedback from nearly 20 million Gemini^6^ responses, again confirming the preservation of text quality. We hope that the availability of SynthID-Text^7^ will facilitate further development of watermarking and responsible use of LLM systems.

## Abstract (web summary)

A scheme for watermarking the text generated by large language models shows high text quality preservation and detection accuracy and low latency, and is feasible in large-scale-production settings.

## Main

Large language models (LLMs) are widely adopted tools for synthetic text generation, finding applications in language-based assistants, code generation, writing support and various other domains. As LLMs advance in quality, coherence, coverage and expertise, it can become difficult to distinguish synthetically generated text from human-written text^1–3^. Given the widespread use of LLMs in education, software development and web content generation, identification and attribution of LLM text is critical to ensure safe and responsible use of the technology^8–11^.

Multiple strategies have emerged to address this problem. One is a retrieval-based approach, which involves keeping a growing record of all generated texts and checking against it for matches^12^. This requires scale and coordination, and raises privacy concerns as it requires accessing and storing all LLM interactions. Another approach is post hoc detection, often using the statistical features of text or training a machine-learning-based classifier to distinguish human-written from artificial-intelligence-generated text^13–15^. This approach can potentially provide broader detection without the need for record-keeping or any intervention at the text generation stage. However, post hoc detection systems can themselves be computationally expensive to run, and their practical usage is limited by their inconsistent performance^16^. In particular, they are known to perform poorly on out-of-domain data and may have higher false-positive rates for certain groups, such as non-native speakers^17^. Furthermore, such classifiers fundamentally rely on underlying differences between machine and human text, which may diminish as LLMs improve. This necessitates continuous maintenance of the classifier, including re-training and re-calibrating.

A third approach is text watermarking—a way of marking the generated text so that it can subsequently be identified. Text watermarking can be done during the generative process (generative watermarking), by editing already generated text (edit-based watermarking) or by altering the LLM’s training data (data-driven watermarking)^4^. Edit-based watermarking frequently relies on applying rule-based transformations such as synonym substitution or inserting special Unicode characters^18^, whereas data-driven watermarking involves training the LLM on specific trigger phrases^19^. With data-driven watermarking, the model outputs are watermarked only when the model is prompted with specific trigger phrases; the primary objective is to identify unauthorized misuse of LLMs rather than attributing pieces of text to an LLM more broadly. Furthermore, both of these approaches can leave noticeable artefacts in the text^4^.

When watermarking an LLM deployed within a large-scale-production setting, it is important to carefully control any impact from watermarking on text quality and, by extension, user experience. It is also important that we are able to watermark with minimal computational costs. To meet both of these criteria, this work focuses on generative watermarking, which allows us to embed watermarks while carefully controlling the impact on quality and maintaining low computational cost. However, we note that no text detection method is foolproof, and many of the approaches discussed in this section are complementary and can be used in conjunction^4^.

**Fig. 1:**
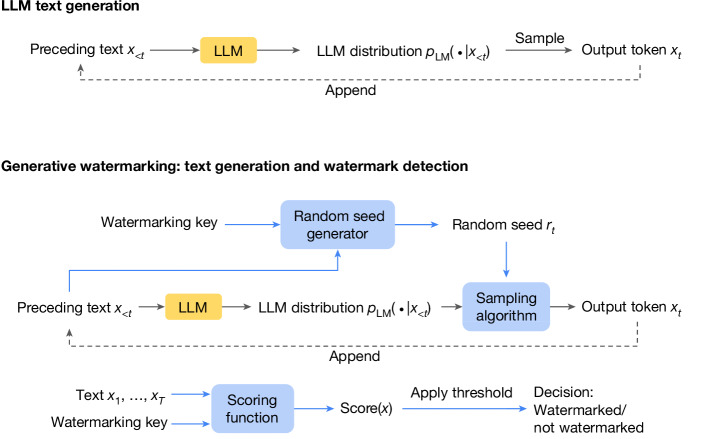
Overview of LLM text generation and generative watermarking. Top: LLM text generation typically involves generating text from left to right by repeatedly sampling from the LLM distribution. Bottom: a generative watermarking scheme typically consists of the three components, in the blue boxes: random seed generator, sampling algorithm and scoring function. These can be used to provide a text generation method and a watermark detection method. In the SynthID-Text generative watermarking scheme, we use the Tournament sampling algorithm.

Generating text with an LLM is often autoregressive: the LLM assigns probabilities to the elements (tokens) of the vocabulary and then selects the next token by sampling according to these probabilities conditional on text generated so far (Fig. 1, top). Generative watermarking (Fig. 1, bottom) works by carefully modifying the next-token sampling procedure to inject subtle, context-specific modifications into the generated text distribution. Such modifications introduce a statistical signature into the generated text; during the watermark detection phase, the signature can be measured to determine whether the text was indeed generated by the watermarked LLM. A key benefit of the approach is that the detection process does not require performing computationally expensive operations or even access to the underlying LLM (which is often proprietary).

In this work, we propose a generative watermarking scheme, SynthID-Text, which builds on previous generative watermarking components, but uses a novel sampling algorithm, Tournament sampling. SynthID-Text can be configured to be non-distortionary (preserving text quality) or distortionary (improving watermark detectability at the cost of text quality). We show that in both settings, SynthID-Text provides improved detection rates, compared with the best existing approaches in each category. We show empirically that non-distortionary SynthID-Text preserves text quality, including through a large-scale user feedback assessment over nearly 20 million responses from live Gemini interactions. Consequently, SynthID-Text has been used to watermark Gemini and Gemini Advanced^20^. This serves as practical proof that generative text watermarking can be successfully implemented and scaled to real-world production systems, serving millions of users and playing an integral role in the identification and management of artificial-intelligence-generated content.

Furthermore, we provide an algorithm to combine generative watermarking with speculative sampling^5^—a frequently used technique to increase LLM text generation speed—allowing for the integration of SynthID-Text into large-scale production systems with negligible additional computational overhead.

## Watermarking with SynthID-Text

LLMs generate text based on preceding context (for example, a response to a provided prompt). More precisely, given a sequence of input text *x*_<*t*_ = *x*_1_, …, *x*_*t*−1_ consisting of *t* − 1 tokens from a vocabulary *V*, the LLM computes the probability distribution *p*_LM_(⋅∣*x*_<*t*_) of the next token *x*_*t*_ given the preceding text *x*_<*t*_. To generate the full response, *x*_*t*_ is sampled from *p*_LM_(⋅∣*x*_<*t*_), and the process repeats until either a maximum length is reached or an end-token is generated. The process is illustrated in Fig. 1 (top).

A generative watermarking scheme typically comprises three components: a random seed generator, a sampling algorithm and a scoring function^21^. As shown in Fig. 1 (bottom), the random seed generator provides a random seed *r*_*t*_ on each generation step *t* (potentially based on the preceding text along with the watermarking key), and the sampling algorithm uses *r*_*t*_ to sample the next token *x*_*t*_ from *p*_LM_(⋅∣*x*_<*t*_). Importantly, the sampling algorithm introduces correlations between *r*_*t*_ and *x*_*t*_; during watermark detection, these correlations are measured by the scoring function. Given a piece of text and the watermarking key, the scoring function provides a score that quantifies the strength of the correlation (that is, the watermarking evidence); this can be compared with a threshold to determine whether the text originates from the watermarked LLM.

**Fig. 2:**
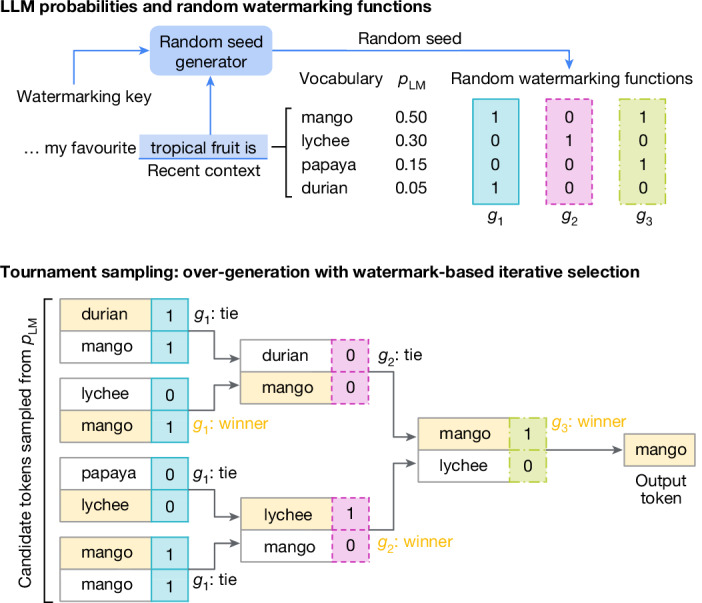
SynthID-Text’s Tournament-based watermarking. Top: to generate a new token *x*_*t*_, we first score each token in the vocabulary using multiple (in this case, *m* = 3) random watermarking functions *g*_1_, …, *g*_*m*_. These assign random values using a random seed, which is generated based on both the recent context and a watermarking key. Bottom: then, we choose the next token using a tournament process. First, we sample 2^*m*^ = 8 (possibly non-unique) tokens from *p*_LM_(⋅∣*x*_<*t*_). These are split into pairs of competing tokens; in each pair, the highest scoring one (based on *g*_1_) is chosen, breaking ties randomly. The resulting tokens compete in the next layer, where winners are chosen based on *g*_2_, until in the last tournament layer the final winner is selected based on *g*_*m*_: this becomes the next generated token *x*_*t*_.

In this work, we present the sampling algorithm Tournament sampling, which is described in the following section. For the random seed generator, in our experiments we use the existing sliding-window method^22,23^, where the random seed is a hash of the most recent *H* tokens (*x*_*t*−*H*_, …, *x*_*t*−1_; we use *H* = 4) along with the watermarking key (Fig. 2, top); but we note that Tournament sampling can be paired with any random seed generator. We experiment with several scoring functions, some of which are from existing work and others are from this work; we discuss them in the following sections. Together, our generative watermarking scheme is called SynthID-Text.

### SynthID-Text’s Tournament sampling approach

The key idea of Tournament sampling is to use a tournament-like process to choose an output token that scores highly with respect to some random watermarking functions. An illustration is given in Fig. 2 (top). First, we take the random seed *r*_*t*_ provided by the random seed generator. This seed is passed to *m* (in this case, *m* = 3) watermarking functions *g*_1_, *g*_2_, *g*_3_, …, *g*_*m*_—these are independent pseudorandom number functions that assign a score *g*_*ℓ*_(*x*_*t*_, *r*_*t*_) (in this case, a 0 or 1) to any candidate token *x*_*t*_ ∈ *V*.

In the second stage (Fig. 2, bottom), we start by sampling *M* = 2^*m*^ candidate tokens from the LLM distribution *p*_LM_(⋅∣*x*_<*t*_) (some tokens may appear multiple times): these are the initial participants of the *m*-layer tournament. We randomly divide these candidates into *M*/2 pairs, and, in the first tournament layer, in each pair the token with the higher score under *g*_1_(⋅, *r*_*t*_) is selected, and the other discarded (any ties are broken randomly). The remaining *M*/2 tokens are regrouped randomly into *M*/4 pairs, and the function *g*_2_(⋅, *r*_*t*_) determines the winners for this second tournament layer. This iterative process continues until one token emerges as the final winner, which becomes the output token *x*_*t*_. A formal description of Tournament sampling is given in Algorithm 2 in Methods.

### Watermark detection

By design, Tournament sampling selects a token from the LLM distribution that is likely to score higher under the random watermarking functions *g*_1_(⋅, *r*_*t*_), …, *g*_*m*_(⋅, *r*_*t*_). To detect whether a piece of text *x* = *x*_1_, …, *x*_*T*_ is watermarked, we measure how highly *x* scores with respect to these functions. Specifically, we compute the mean *g*-values of the text:

1 $$\,\text{Score}\,(x)=\frac{1}{mT}\mathop{\sum }\limits_{t=1}^{T}\mathop{\sum }\limits_{\ell =1}^{m}{g}_{\ell }({x}_{t},{r}_{t}).$$

Given the selection of tokens *x*_*t*_ based on higher *g*-values, we expect watermarked text generally to score higher under this score than unwatermarked text.

There are two primary factors that affect the detection performance of the scoring function. The first is the length of the text *x*: longer texts contain more watermarking evidence, and so we have more statistical certainty when making a decision. The second is the amount of entropy in the LLM distribution when it generates the watermarked text *x*. For example, if the LLM distribution is very low entropy, meaning it almost always returns the exact same response to the given prompt, then Tournament sampling cannot choose tokens that score more highly under the *g* functions. In short, like other generative watermarks^21^, Tournament sampling performs better when there is more entropy in the LLM distribution, and is less effective when there is less entropy. In Supplementary Information section H, we provide a theoretical analysis describing the watermarking strength of a layer of Tournament sampling as a function of a certain kind of entropy; similar analyses have been done for other generative watermarks^23–25^. The entropy of the LLM distribution itself depends on several factors, including the model—for example, larger or more advanced models tend to be more certain and thus lower entropy^21^, and reinforcement learning from human feedback can reduce entropy (also known as ‘mode collapse’)^26^. Other factors that affect LLM distribution entropy include the prompts, the temperature and other decoding settings such as top-*k* and top-*p* sampling settings (see ‘The LLM distribution’ in Methods).

Increasing the number of tournament layers *m* provides additional watermarking evidence per token, and decreases the variance of the score in equation (1). This allows SynthID-Text to provide better detectability than other methods (see ‘Evaluation’). However, detectability does not increase indefinitely with the number of layers. Each layer of the tournament uses some of the available entropy to embed a watermark, and the strength of the watermark corresponding to a layer diminishes deeper into the tournament. For our experiments, we generally use *m* = 30 layers unless otherwise stated; see Supplementary Information section C.1 for full details.

Finally, we note that there are other scoring functions beyond equation (1); in Supplementary Information section A, we describe several others, and find that some can improve detection performance.

### Preserving the quality of generative text

As previously mentioned, a watermarking scheme can be non-distortionary, a property relating to quality preservation; however, the phrase and its variants have been used in the literature to mean several distinct definitions^24,25,27^, causing some confusion. In this work, we resolve the confusion by providing clear definitions of non-distortion, from weakest to strongest. The weakest version is single-token non-distortion, which says that, on average over the random seed *r*_*t*_, the distribution of the output token *x*_*t*_ generated by the watermarking sampling algorithm is equal to the original LLM distribution *p*_LM_(⋅∣*x*_<*t*_) (Fig. 1). Stronger versions of non-distortion expand this definition to one or more sequences of text, ensuring that on average the probability of the watermarking scheme generating a particular text or sequence of texts is the same as for the original LLM. Full definitions are provided in Supplementary Information section G.

In Supplementary Information section G.1, we show that when Tournament sampling is configured with exactly two ‘competitors’ for each match in the tournament (as in the example in Fig. 2), then Tournament sampling is single-token non-distortionary. Furthermore, in Supplementary Information section G.2, we show that by applying repeated context masking^27^, we can make the scheme non-distortionary for one or more sequences. Choosing the level of non-distortion involves a trade-off; weaker levels of non-distortion can reduce text quality and diversity, whereas stronger levels of non-distortion can reduce detectability and increase computational complexity (Supplementary Information section G.3). For our experiments, we configure SynthID-Text to be single-sequence non-distortionary; this preserves text quality and provides good detectability, while having some reduction to inter-response diversity. We call this configuration ‘non-distortionary SynthID-Text’ (and where not otherwise specified, ‘SynthID-Text’ also refers to this).

Alternatively, for instances where strong watermark detectability is critical, SynthID-Text can take a distortionary configuration that provides higher detectability, at the cost of some quality loss. In this configuration of Tournament sampling, there are more than two competitors in each match of the tournament (a formal definition is given in Algorithm 2 in Methods). We show that in this case, Tournament sampling is distortionary at the token level (Supplementary Information section G.1); however, it applies a stronger watermark (Supplementary Information section H.3). We call this configuration ‘distortionary SynthID-Text’.

In ‘Evaluation’, we compare non-distortionary and distortionary SynthID-Text to the best existing methods in each category and show that SynthID-Text provides better detectability in both categories.

### Ensuring computational scalability

Generative watermarking schemes (Fig. 1, bottom) are typically computationally inexpensive as the text generation process involves a modification to only the sampling layer, which is often negligible in the context of the LLM’s forward pass. For Tournament sampling, in some cases, it is more efficient to use a vectorized implementation, which we describe in Supplementary Information section E. We provide a theoretical complexity analysis of both implementations, and existing baselines in Supplementary Information section F. Overall, we show empirically in ‘Evaluation’ that, in practice, SynthID-Text induces negligible additional latency.

In large-scale productionized systems, the text generation process is often more complex than the simple loop depicted in Fig. 1 (top). For example, productionized systems often use speculative sampling^5^, a method to accelerate text generation from large models. Speculative sampling works by having a smaller draft model propose the next few tokens; these are then checked against the large target model, which either accepts or rejects the proposed tokens. Combining generative watermarking with speculative sampling is an important step to make watermarking practically useful to production systems; however, to our knowledge, it has not yet been investigated.

To make progress in this area, we propose two generative watermarking with speculative sampling algorithms, which can combine a generative watermarking scheme with speculative sampling (Supplementary Information section I). First, we propose high-detectability watermarked speculative sampling, which preserves the detectability of the watermark, but may decrease the efficiency (and thus increase the overall latency) of speculative sampling (Supplementary Information section I.4). Alternatively, we propose fast watermarked speculative sampling, which (provided the watermark is single-token non-distortionary) preserves the efficiency of speculative sampling, but may decrease the detectability of the watermark (Supplementary Information section I.5). For this approach, we also propose a learned Bayesian scoring function that improves the detectability of this method (Supplementary Information section I.5.2). Fast watermarked speculative sampling is most helpful when speed is important in production environments.

## Evaluation

We compare SynthID-Text to, at the time of writing, the best-performing non-distortionary and distortionary generative text watermarking schemes and show empirically that SynthID-Text provides superior detectability in both categories. In the non-distortionary category, we compare against Gumbel sampling^22,24^, and in the distortionary category, we compare against the Soft Red List sampling algorithm^23^; see Supplementary Information section B.1 for a full description and discussion of how we chose our baselines. To create a like-for-like comparison, we focus on comparing our sampling algorithm, Tournament sampling, against the Gumbel and Soft Red List sampling algorithms while keeping the other parts of the watermarking scheme the same (Fig. 1). Accordingly, for all baselines we use the same sliding-window random seed generator, and the same repeated context masking methodology as described in ‘Watermarking with SynthID-Text’—this means that (like non-distortionary SynthID-Text) the Gumbel baseline is single-sequence non-distortionary and preserves text quality. Furthermore, we note that the hashing and scoring schemes from refs. ^24,25^ can be directly adapted with SynthID-Text, and a detailed comparison of the benefits and drawbacks of various hashing and scoring procedures (for example, the edit-distance based scoring as in ref. ^24^) is beyond the scope of this work.

In the remainder of this section, we empirically demonstrate that SynthID-Text, like some other generative watermarks, has several key desirable properties (quality and scalability) that enable its deployment in large-scale production, while also offering additional desirable properties such as improved detectability and diversity of the generated text. First, we show that (like other non-distortionary watermarks) non-distortionary SynthID-Text preserves response quality; our evaluations include the first large-scale human evaluation in a productionized system. Then, across multiple models, we show that SynthID-Text provides improved detection performance while also preserving a greater amount of the underlying diversity within the LLM responses. We also show that SynthID-Text, similar to other generative watermarking schemes, has negligible computational impact in the context of a large-production LLM.

### SynthID-Text preserves quality including in a large-scale-production system

To evaluate the production readiness of non-distortionary SynthID-Text, we ran a live experiment with the Gemini production system (previously known as Bard). A random fraction of queries were routed to a watermarked model and an equivalent number to the unwatermarked counterpart. The Gemini user interface allows users to provide feedback on model responses via a thumbs-up (good response) and a thumbs-down (bad response). We analysed approximately 20 million watermarked and unwatermarked responses and computed the thumbs-up and thumbs-down rates (both as a fraction of the total number of thumbs-up and thumbs-down feedback received). We found that the thumbs-up rate for the two models differed by 0.01% (with the watermarked model being higher); and the thumbs-down rate differed by 0.02% (with the watermarked model being lower). We found both of these differences to be statistically insignificant, and well within the 95% confidence intervals.

From this experiment, we conclude that over a wide variety of real chatbot interactions, the difference in response quality and utility, as judged by humans, is negligible. Subsequently, non-distortionary SynthID-Text has been productionized and is currently watermarking responses in Gemini and Gemini Advanced. To the best of our knowledge, this evaluation represents the first systematic watermarking investigation of its kind within a large-scale production system.

To provide a reproducible human evaluation, we also run a smaller-scale controlled human preference test, for which we also publish the collected data. In this experiment, we ask raters to compare watermarked versus unwatermarked Gemma 7B-IT responses to 3,000 ELI5 questions, assessing five aspects of response quality in a side-by-side comparison. For all five aspects—grammaticality/coherence, relevance, correctness, helpfulness and overall quality—we find no significant difference in rater preference (Extended Data Table 1). This holds both in a three-way analysis that includes tie ratings and conducts a trinomial test, as well as when restricting the analysis to the non-tie responses, using bootstrap resampling over the watermarked versus unwatermarked preference ratio (full details in Supplementary Information section C.4).

To further validate the quality-neutral property of non-distortionary SynthID-Text, we conduct additional automatic evaluations across different models and metrics. We find no significant difference between non-distortionary SynthID-Text and the equivalent unwatermarked model in terms of perplexity or performance on automated benchmarks. Full details are provided in Supplementary Information section C.5.

To summarize: human quality feedback, both from a large-scale live experiment and a small-scale controlled study, perplexity statistics and standard model capability benchmarks all indicate that non-distortionary SynthID-Text causes no loss in text quality.

### SynthID-Text provides better detectability than existing watermarks

We evaluate watermark detectability empirically across several publicly available models, including the instruction-tuned (IT) variants of Gemma 2B and Gemma 7B^28^, and the Mistral 7B-IT^29^ model (see ‘LLMs and LLM configurations’ in Methods for details). We prompt the models with questions from the ELI5 dataset^30^ (see ‘Data’ in Methods).

**Fig. 3:**
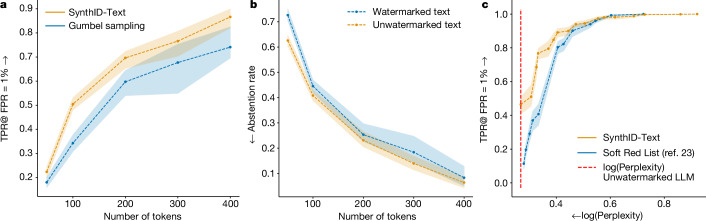
Detection performance of SynthID-Text. **a**, For non-distortionary watermarking, we compare watermark detectability as a function of text length (number of tokens), for non-distortionary SynthID-Text and the Gumbel sampling^22,24^ watermark. Watermark detectability is measured using the true-positive rate (TPR) when the false-positive rate (FPR) is set to 1%. Responses are generated from Gemma 7B-IT with temperature = 0.7 using prompts from the ELI5 dataset; other models and temperatures are provided in Extended Data Fig. 1. **b**, The fraction of watermarked and unwatermarked texts that the selective prediction mechanism (Supplementary Information section C.8) abstains on to achieve a true-positive rate of 95% and a false-positive rate of 1%. The mechanism makes predictions only when the scoring function is confident, and is thus able to maintain a low error rate when making predictions, at the cost of abstaining on some of the data. The model set-up and the prompts are same as that in **a**; other temperatures are in Extended Data Fig. 3. **c**, For distortionary watermarking, we compare detectability of watermarks that allow text quality to be traded off against detectability by adjusting a strength hyperparameter. Texts are of length 200 tokens generated by Gemma 7B-IT with temperature = 0.7; other text lengths and temperatures are provided in Extended Data Fig. 2. Compared with the distortionary Soft Red List watermark^23^, distortionary SynthID-Text offers a more favourable trade-off, with substantially higher detection rates for the same effect on text quality as measured by log(perplexity). The arrows indicates the direction for performance improvement. The dashed lines correspond to a bootstrap estimate (500 resamples) of the mean of the metric on the *y* axis and the shaded regions correspond to the 90% confidence interval on the mean estimate.

**Extended Data Fig. 1:**
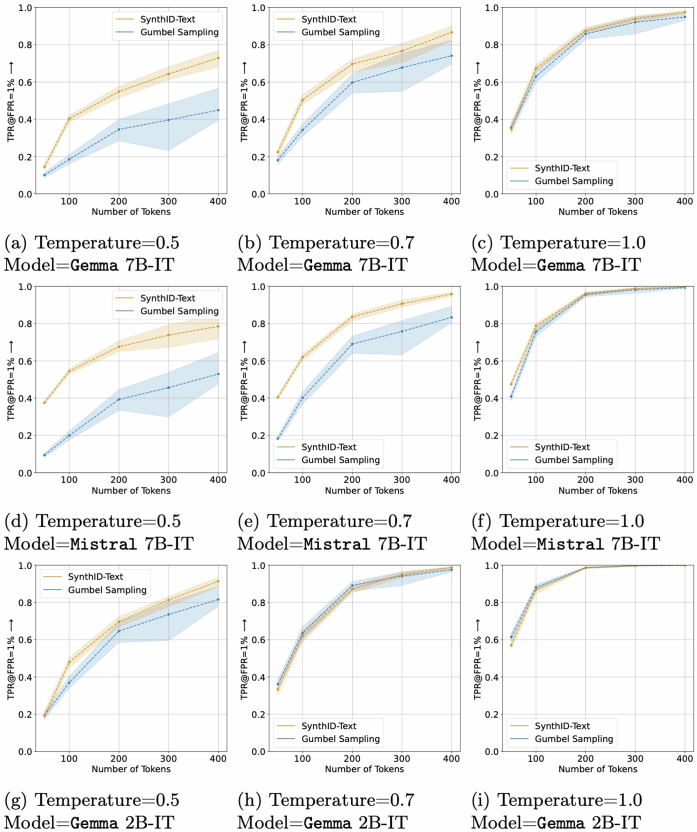
Detectability comparison of non-distortionary SynthID-Text vs Gumbel sampling, for additional model and temperature combinations. We show a range of temperatures from 0.5–1.0, which is the range most often used in practice. SynthID-Text generally provides improved detection over Gumbel sampling, with the improvements being greatest in lower entropy settings (lower temperature and larger models). The dashed lines correspond to a bootstrap estimate (500 resamples) of the mean true positive rate (TPR) at a false positive rate of 1%, and the shaded regions correspond to the 90% confidence interval on the mean estimate.

**Extended Data Fig. 4:**
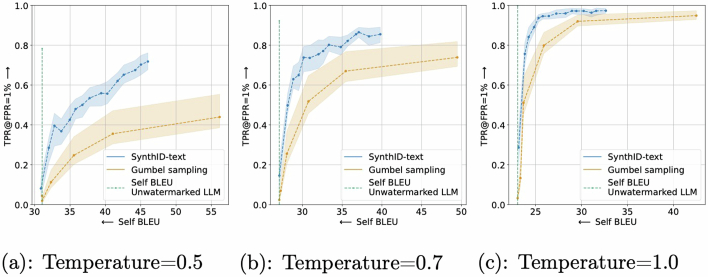
Comparing the tradeoff between diversity and detectability for non-distortionary SynthID-Text and Gumbel sampling. Diversity is indicated by low Self-BLEU and detectability is measured by true positive rate when false positive rate is fixed at 1% (TPR@FPR=1%). Texts are length 400 tokens, generated from Gemma 7B-IT at three different model temperatures. We observe that SynthID-Text provides a more favorable trade-off across temperatures. Dashed lines correspond to a bootstrap estimate (500 resamples) of the mean TPR@FPR=1%, and shaded regions correspond to the 90% confidence interval on the mean estimate. See Supplementary Information section C.3 for details.

In the non-distortionary category, Fig. 3a shows that non-distortionary SynthID-Text provides better detectability than Gumbel sampling, for the same length text. We find that the SynthID-Text’s improvement over Gumbel sampling is greater in lower-entropy settings (for example, lower temperatures); when the entropy is higher, the detectability of the two methods is more comparable (Extended Data Fig. 1). In Extended Data Fig. 4, we also show that although both non-distortionary SynthID-Text and the Gumbel sampling baseline reduce inter-response diversity, SynthID-Text provides a better diversity/detectability trade-off than Gumbel sampling. In scenarios where low error rates are desirable, we can use a selective prediction mechanism (Supplementary Information section C.8) to abstain on samples for which the scoring function is uncertain, thus achieving the desired error rates on the remaining data (Fig. 3b).

In the distortionary category, we compare the trade-off of detectability and text quality for distortionary SynthID-Text and Soft Red List. Both methods have a strength parameter that controls this trade-off; in Fig. 3c, we see that the trade-off is more favourable for distortionary SynthID-Text.

### SynthID-Text has minimal computational impact

As discussed in ‘Watermarking with SynthID-Text’ and Supplementary Information section F, Tournament sampling does in some cases have greater computational complexity than Gumbel or Soft Red List sampling, but these differences are minimal relative to the cost of generating text from an LLM. For example, the Gemma 7B-IT model served on 4 v5e tensor processing units^31^ generates text at a rate of 15.527 ms per token; this increases to 15.615 ms per token with 30-layer Tournament sampling, a latency increase of only 0.57%. In comparison, Gumbel sampling increases latency by 0.26% and Soft Red List by 0.28%. Furthermore, the computational complexity of all three watermarks remains constant even as the LLM grows. Thus, as large production models can grow by orders of magnitude larger than Gemma 7B in terms of computational complexity, so too does the relative complexity of watermarking shrink by orders of magnitude.

As described in ‘Watermarking with SynthID-Text’, we propose an algorithm—fast watermarked speculative sampling—to integrate generative watermarking with speculative sampling and thus enable fast deployment of watermarked LLMs at scale. We evaluate our algorithm with non-distortionary SynthID-Text, using Gemma 7B-IT as the target model and Gemma 2B-IT as the smaller draft model that proposes three ‘lookahead’ tokens at a time. When paired with a non-distortionary watermark (Supplementary Information section I.3), fast watermarked speculative sampling is theoretically guaranteed to preserve the acceptance rate (that is, the average number of lookahead tokens accepted by the target model). We confirm this experimentally, finding that the acceptance rate (and thus overall latency) is very similar with and without watermarking (Supplementary Information section I.5.3). Although we ran our experiment with non-distortionary SynthID-Text, we expect the same result would hold for any non-distortionary generative watermark.

## Discussion

We have introduced SynthID-Text, a method for watermarking LLM text. SynthID-Text uses certain elements introduced in previous work^22,23,27^, but differs in the use of the sampling algorithm, Tournament sampling, which we find provides superior detectability compared with existing methods. SynthID-Text comes with rigorous and customizable non-distortion properties that can be configured to guarantee text quality preservation; we confirm this empirically, including via real user feedback measured over approximately 20 million Gemini chatbot interactions. We have also proposed an algorithm to combine generative watermarking with speculative sampling, thus enabling the efficient deployment of generative watermarks in high-performance, large-scale-production LLMs.

### Limitations

Generative watermarks such as SynthID-Text provide several advantages compared with other approaches. For example, in Supplementary Information section C.7, we show that SynthID-Text performs consistently across different languages. In comparison, a post hoc detector performs poorly on languages that its underlying machine-learning model was not trained on. However, generative watermarks such as SynthID-Text do not offer a complete solution to artificial-intelligence text detection; rather they are complementary to other approaches. In particular, generative watermarks require coordination between actors running the LLM text generation services to apply the watermark. To detect artificial-intelligence-generated text produced by other actors who do not to implement watermarking, other approaches are required, such as post hoc detection. Furthermore, the rise of open-source models presents a challenge, as enforcing watermarking on these models deployed in a decentralized manner is difficult. Another limitation of generative watermarks is their vulnerability to stealing, spoofing and scrubbing attacks, which is an area of ongoing research^32^. In particular, generative watermarks are weakened by edits to the text, such as through LLM paraphrasing^33^—although this usually does change the text significantly. We provide evaluations of SynthID-Text’s performance under edits and paraphrasing in Supplementary Information section C.6.

### Conclusion

Overall, our work provides proof of the real-world viability of generative text watermarks. SynthID-Text has been productionized in the user-facing Gemini and Gemini Advanced chatbots, which is, to our knowledge, the first deployment of a generative text watermark at scale, serving millions of users. As such, our work sets a practical milestone for accountable, transparent and responsible LLM deployment.

## Methods

### Detailed SynthID-Text method

In this section, we provide a detailed description of SynthID-Text.

#### The LLM distribution

Most LLMs are autoregressive, providing the probability *p*_LM_(*x*_*t*_∣*x*_<*t*_) of the next token *x*_*t*_ given the text so far *x*_<*t*_. Text is typically generated from the LLM using an autoregressive decoding method, which optionally modifies the LLM distribution *p*_LM_(⋅∣*x*_<*t*_) before sampling from it. Such modifications include top-*k* and top-*p*^34^ sampling, which truncate *p*_LM_(⋅∣*x*_<*t*_) to the *k* most likely tokens or the tokens covering the top-*p* probability mass; this can be combined with applying a temperature parameter *τ* (ref. ^35^). Although these modifications increase or decrease the amount of entropy in *p*_LM_(⋅∣*x*_<*t*_), SynthID-Text is compatible with any autoregressive decoding method that has non-zero entropy in the modified distribution. Thus, SynthID-Text is compatible with top-*k* sampling for all *k* ≥ 2, top-*p* sampling for all $p\in \left(0,1\right]$, and all temperatures *τ* > 0.

SynthID-Text is applied after any such modifications have been made, so for the purposes of this paper we define the LLM distribution *p*_LM_(⋅∣*x*_<*t*_) to be the distribution after any such modifications.

##### Definition 1 (LLM distribution)

*Given an autoregressive LLM*, *an autoregressive decoding method*, *and x*_<*t*_ = *x*_1_, …, *x*_*t*−1_, *a sequence of tokens from the vocabulary* *V*, *the LLM distribution p*_LM_(⋅∣*x*_<*t*_) *is the probability distribution from which the decoding method samples the next token* *x*_*t*_ ∈ *V*.

#### Watermarking framework

We present SynthID-Text as comprising a random seed generator, a sampling algorithm and a scoring function; this is similar to the generative watermarking framework of ref. ^21^. Intuitively, the sampling algorithm samples text from the LLM in a way that is biased by random seeds provided on each step by the random seed generator; later we can identify the watermark by detecting this bias through the scoring function. We describe the random seed generator and sampling algorithm in this section and describe several scoring functions in Supplementary Information section A. See Supplementary Information section B for a detailed discussion of related generative watermarking approaches.

#### Random seed generator

To generate a piece of watermarked text *x*_1_, …, *x*_*T*_, we require a sequence of random seeds ${r}_{1},\ldots ,{r}_{T}\in {\mathcal{R}}$ (where ${\mathcal{R}}$ is the space of all random seeds) to bias the sampling from the LLM distribution on each step. The random seed generator is the process by which we generate these random seeds. One approach is to make the random seed generator a deterministic function *f*_*r*_ that takes as input the sequence of tokens so far *x*_<*t*_ = *x*_1_, …, *x*_*t*−1_ and a watermarking key *k* and outputs a random seed ${r}_{t}:={f}_{r}({x}_{ < t},k)\in {\mathcal{R}}$. Randomizing the key *k* should randomize the seed; that is, for all ${x}_{ < t},{{\mathbb{P}}}_{{k \sim }\text{Unif}({\mathcal{R}})}\,[\,{f}_{r}({x}_{ < t},k)]=\text{Unif}\,({\mathcal{R}})$.

There are several possible choices for *f*_*r*_ (ref. ^21^); for our experiments, we use the sliding window *f*_*r*_(*x*_<*t*_, *k*) ≔ *h*(*x*_*t*−*H*_, …, *x*_*t*−1_, *k*), which is a hash function *h* of the last *H* tokens (for some context length *H* ≥ 1) and of the key *k*. This random seed generator is the same as that used by refs. ^22^^,^^23^. In this work, we also assume the watermarking key *k* and random seed *r*_*t*_ exist in the same space of *n*_sec_-bit integers, where *n*_sec_ is the security parameter.

##### Definition 2 (random seed space, random seed distribution)

*Given a security parameter n*_sec_, *the random seed space* ${\mathcal{R}}={\{0,1\}}^{{n}_{\text{sec}}}$ *is the space of all* *n*_sec_*-bit integers*. *The random seed distribution is the uniform distribution over all such integers* $\,\text{Unif}\,({\mathcal{R}})$.

We also assume that the family of functions ${\{h(\cdot ,\ldots ,\cdot ,k)\}}_{k\in {\mathcal{R}}}$ is a pseudorandom function family, meaning that (1) *h*(*x*_*t*−*H*_, …, *x*_*t*−1_, *k*) is efficiently computable for any *x*_*t*−*H*_, …, *x*_*t*−1_ and *k*, and (2) the distribution of ${\{h(\cdot ,\ldots ,\cdot ,k)\}}_{{k \sim }\text{Unif}({\mathcal{R}})}$ is computationally indistinguishable from a function sampled uniformly randomly from the set of all functions from *V*^*H*^ to ${\{0,1\}}^{{n}_{\text{sec}}}$.

#### *g*-values

As illustrated in Fig. 2, Tournament sampling requires *g*-values to decide which tokens win each match in the tournament. Intuitively, we want a function that takes a token *x* ∈ *V*, a random seed $r\in {\mathcal{R}}$ and the layer number *ℓ* ∈ {1, …, *m*}, and outputs a *g*-value *g*_*ℓ*_(*x*, *r*) that is a pseudorandom sample from some probability distribution *f*_*g*_ (the *g*-value distribution).

For example, in Fig. 2, the *g*-value distribution is Bernoulli(0.5). Given the random seed *r*, *g*_*ℓ*_(*x*, *r*) produces pseudorandom *g*-values of 0 or 1 for each token *x* in the vocabulary, for each layer *ℓ* = 1, 2, 3. In this paper, we primarily use the Bernoulli(0.5) *g*-value distribution, although we also explore Uniform[0, 1]. In general, any *g*-value distribution can be chosen, as a hyperparameter of the Tournament sampling method.

##### Definition 3 (*g*-value distribution)

*The* *g-value distribution is a probability distribution of any real-valued random variable*. *We write* *F*_*g*_ *to denote the cumulative distribution function, and f*_*g*_ *to denote the probability density function (if continuous) or probability mass function (if discrete)*.

Next, we need a way to produce a hash $h(x,{\ell },r)\in {\mathcal{R}}$ of a token *x* ∈ *V*, an integer *ℓ* ∈ {1, …, *m*} and a random seed $r\in {\mathcal{R}}$. Let’s assume we have a pseudorandom function family ${\{h(\cdot ,\cdot ,r)\}}_{r\in {\mathcal{R}}}$ similar to the one described in the ‘Random seed generator’ section, such that the distribution of ${\{h(\cdot ,\cdot ,r)\}}_{{r \sim }{\rm{Unif}}({\mathcal{R}})}$ is computationally indistinguishable from a function sampled uniformly randomly from the set of all functions from *V* × [*m*] to ${\{0,1\}}^{{n}_{\sec }}$.

##### Definition 4 (*g*-value)

*Given a* *g*-*value distribution with cumulative density function.* *F*_*g*_, *a random seed* $r\in {\mathcal{R}}$, *and integer* *ℓ* ∈ 1, …, *m*, *the layer-ℓ* *g-value of a token x* ∈ *V* *is given by*:

$${g}_{{\ell }}(x,r)\,:={F}_{g}^{-1}\,\left(\frac{h(x,{\ell },r)}{{2}^{{n}_{\text{sec}}}}\right),$$

*where* ${F}_{g}^{-1}$ *is the generalized inverse distribution function of F*_*g*_, *and* *h* *is a hash function as described above*.

Intuitively, Definition 4 says that we take a hash *h*(*x*, *ℓ*, *r*) of *x*, *ℓ* and *r*, which gives us a uniformly distributed *n*-bit integer, and divide it by 2^*n*^ to get a number in [0, 1]. For large *n*, this converges to a uniformly distributed number in [0, 1]. We then perform inverse transform sampling to turn this number into a sample from the *g*-value distribution given by *F*_*g*_.

#### Tournament sampling algorithm

##### Definition 5 (watermarking sampling algorithm)

*In a watermarking scheme*, *a sampling algorithm* ${\mathcal{S}}:\Delta V\times {\mathcal{R}}\to V$ *is an algorithm that takes as input a probability distribution* *p* ∈ Δ*V* *and a random seed* $r\in {\mathcal{R}}$ *and returns a token* ${\mathcal{S}}(p,r)\in V$. *If* ${\mathcal{S}}$ *always returns the same token given the same* *p* *and* *r*, *it is deterministic*. *Otherwise*, ${\mathcal{S}}$ *is probabilistic*.

We propose a new probabilistic sampling algorithm called Tournament sampling. We present the simplest, single-layer version of Tournament sampling in Algorithm 1. Instead of sampling directly from *p*_LM_(⋅∣*x*_<*t*_), we sample *N* tokens from *p*_LM_(⋅∣*x*_<*t*_), compute their *g*-values as described in the previous section and choose uniformly among those that have the maximal *g*-value.

Algorithm 2 presents the full multilayer version of Tournament sampling, which has an additional hyperparameter *m*, the number of layers. The process can be thought of as a knockout tournament with *m* stages, where each match is an instantiation of the single-layer algorithm; this continues until there is one winner. Importantly, each layer *ℓ* of the tournament uses different *g*-values *g*_*ℓ*_(⋅, *r*_*t*_) to decide the winners. Figure 2 gives a concrete example for *m* = 3 layers, *N* = 2 samples and a Bernoulli(0.5) *g*-value distribution.

##### Algorithm 1

Sampling a token with single-layer Tournament sampling

**Require**: LLM distribution *p*_LM_(⋅∣*x*_<*t*_), random seed ${r}_{t}\in {\mathcal{R}}$, number of samples *N* ≥ 2, *g* function with *g*-value distribution *f*_*g*_ (see Definition 4).

1: Draw *Y* = [*y*_1_, *y*_2_, …, *y*_*N*_] containing *N* independent samples from *p*_LM_(⋅∣*x*_<*t*_) (may contain repeats).

2: ${Y}^{* }:=\,[\,y\in Y:{g}_{1}(\,y,{r}_{t})=\mathop{\max }\limits_{{y}^{{\prime} }\in Y}{g}_{1}(\,{y}^{{\prime} },{r}_{t})]$ (may contain repeats).

3: Sample *x*_*t*_ ~ Unif(Y*)

4: **return** *x*_*t*_

##### Algorithm 2

Sampling a token with multilayer Tournament sampling.

**Require**: LLM distribution *p*_LM_(⋅∣*x*_<*t*_), random seed ${r}_{t}\in {\mathcal{R}}$, number of samples *N* ≥ 2, *g* function with *g*-value distribution *f*_*g*_ (see Definition 4), number of layers *m* ≥ 1.

1: Draw *N*^*m*^ independent samples ${y}_{0}^{0},{y}_{1}^{0},\ldots ,{y}_{{N}^{m}-1}^{0} \sim {p}_{{\rm{LM}}}(\cdot | {x}_{ < t})$ (may contain repeats).

2: **for** 1 ≤ *ℓ* ≤ *m* **do**

3:  **for** 0 ≤ *j* ≤ *N*^*m*−*ℓ*^ − 1 **do**

4:   $Y:=\,[\,{y}_{Nj}^{{\ell }-1},{y}_{Nj+1}^{{\ell }-1},\ldots ,{y}_{Nj+N-1}^{{\ell }-1}]$ (may contain repeats).

5:   ${Y}^{* }:=\,[\,y\in Y:{g}_{{\ell }}(\,y,{r}_{t})=\mathop{\max }\limits_{{y}^{{\prime} }\in Y}{g}_{{\ell }}(\,{y}^{{\prime} },{r}_{t})]$ (may contain repeats).

6:   Sample $${y}_{j}^{{\ell }} \sim \,{\rm{Unif}}\,({Y}^{* })$$.

7:  **end for**

8: **end for**

9: **return** $${x}_{t}:={y}_{0}^{m}$$

#### Repeated context masking

To generate a full response, we could simply apply Algorithm 2 on every decoding step, using the sliding-window random seed generator (‘Random seed generator’ section) to generate the random seed *r*_*t*_ for each step. However, it is possible that the same window of context, and thus the same random seed might occur more than once (particularly if the sliding-window size *H* is small or the response is long). It has been shown that in this scenario, the watermark can introduce a repeated bias that affects the quality of the text, for example, causing repeating loops^24,25^. One way to avoid this problem is to apply repeated context masking^27^, which prevents the watermark from being applied on step *t* if the context window (*x*_*t*−*H*_, …, *x*_*t*−1_) has been used to watermark previously.

We present the method in Algorithm 3, which we call *K*-sequence repeated context masking. The integer parameter *K* ≥ 1 controls for how long context windows are held in the history. In the simplest case of *K* = 1, we only hold the context history for the duration of generating a single response. For larger integers *K* > 1, we check against a history of contexts used in the last *K* responses. In the extreme case, we could set *K* = *∞* and retain the context history indefinitely. In Supplementary Information section G.2, we show that applying *K*-sequence repeated context masking achieves *K*-sequence non-distortion, an important property for quality preservation. In Supplementary Information section G.3, we discuss the trade-offs of smaller and larger *K*. For most of our experiments we use *K* = 1.

##### Algorithm 3

Generating watermarked responses with sliding-window random seed generation and *K*-sequence repeated context masking.

**Require**: LLM *p*_LM_(⋅∣⋅), context window size *H*, pseudorandom hash function *h*, watermarking key $k\in {\mathcal{R}}$, sampling algorithm ${\mathcal{S}}:\Delta V\times {\mathcal{R}}\to V$, integer *K* ≥ 1, stream of prompts (**x**^1^, **x**^2^, …).

1: **for** *i* ≥ 1 **do**

2:  $${C}_{i}:=\varnothing $$

3:  *t* ≔ *n* where *n* is the length of ${{\bf{x}}}^{i}={{\bf{x}}}_{1}^{i},\ldots ,{{\bf{x}}}_{n}^{i}$

4:  **while** $${{\bf{x}}}_{t}^{i}\ne {\mathtt{EOS}}$$ **do**

5:   *t* ≔ *t* + 1

6:   **if** $$({{\bf{x}}}_{t-H}^{i},\ldots ,{{\bf{x}}}_{t-1}^{i})\in {C}_{i}\cup {C}_{i-1}\cup \cdots \cup {C}_{i-K+1}$$ **then**

7:    Sample $${{\bf{x}}}_{t}^{i} \sim {p}_{{\rm{LM}}}(\cdot | {{\bf{x}}}_{ < t}^{i})$$

8:   **else**

9:    $${r}_{t}:=h({{\bf{x}}}_{t-H}^{i},\ldots ,{{\bf{x}}}_{t-1}^{i},k)$$

10:    Sample $${{\bf{x}}}_{t}^{i}:={\mathcal{S}}({p}_{{\rm{LM}}}(\cdot | {{\bf{x}}}_{ < t}^{i}),{r}_{t})$$

11:    $${C}_{i}:={C}_{i}\cup \{({{\bf{x}}}_{t-H}^{i},\ldots ,{{\bf{x}}}_{t-1}^{i})\}$$

12:   **end if**

13:  **end while**

14:  **return** Response $${{\bf{y}}}^{i}:={{\bf{x}}}_{n+1:t}^{i}$$

15: **end for**

#### Scoring functions

A scoring function takes a piece of text *x*_1_, …, *x*_*T*_ along with the random seeds *r*_1_, …, *r*_*T*_ and computes a score, which can then be compared with a threshold to classify the text as watermarked or unwatermarked. Here the random seeds *r*_*t*_ = *f*_*r*_(*x*_<*t*_, *k*) are from the random seed generator (‘Random seed generator’ section). It is noted that a scoring function only requires access to the tokenized text, the watermarking key *k* and the random seed generator *f*_*r*_; no access to the LLM is required.

For SynthID-Text, we propose several scoring functions, which are in Supplementary Information section A. All the scores are computed from the *g*-values of the text. The simplest of these is the mean score, which is simply the mean of the *g*-values across all timesteps and layers. We also propose a weighted mean score, which re-weights the evidence of each tournament layer. We propose frequentist versions of these scores, which perform a hypothesis test on these means to produce a *P* value. Lastly, we propose a parameterized Bayesian scoring function, which achieves better performance by learning from data (watermarked and unwatermarked texts) to compute the posterior probability that a text is watermarked.

### Experimental details

#### LLMs and LLM configurations

In our experiments, we use the IT variants of the Gemma 2B and 7B models^28^. We also use the v0.2 Mistral 7B-IT model^29^. To generate text, we use top-*k* sampling^36^. Following default settings, we use *k* = 100 for the IT models. We experiment with temperatures of 0.5, 0.7 and 1.0, as varying the temperature changes the entropy of the model, which affects watermark detectability.

#### Data

To prompt our models we use the ELI5^30^ dataset, which consists of English questions that require explanatory multi-sentence answers. This simulates a more task-oriented setting. For experiments with non-distortionary watermarking, our ELI5 test set and the development set each contain sets of 10,000 disjoint prompts that are used to prompt the model to obtain watermarked responses. For experiments with distortionary watermarking, we use 1,500 prompts from ELI5 for the test set to prompt the watermarked model. For the unwatermarked samples used as negatives, we use two disjoint sets of human-written responses to 10,000 questions from the ELI5 for the development and test sets.

#### Text lengths

For some experiments, we evaluate texts of fixed length—for example, 200 tokens. To obtain text of length exactly 200 tokens, we select the subset of texts that are longer than 200 tokens and then truncate them to have exactly 200 tokens.

#### Detectability metric

To report detectability, we use the true-positive rate (TPR) for a fixed false-positive rate (FPR) of *x*%, measured empirically. We denote this metric as TPR @ FPR = *x*%. For example to compute TPR @ FPR = 1%, we take the scores (under some scoring function) of the unwatermarked texts and compute a threshold corresponding to the top-1% highest scores. Then we compute the true-positive rate by measuring the fraction of watermarked texts that score above this threshold. Although some scoring functions allow a precise theoretical guarantee on the false-positive rate—for example, the frequentist scoring functions (Supplementary Information section A.3) which provide a *P* value—in this work we take the empirical approach described above.

#### Random seed generator settings

For all watermarking experiments (including Tournament, Gumbel and Soft Red List sampling algorithms), we use the same sliding-window-based random seed generator described in the ‘Random seed generator’ section, with context window size *H* = 4. We apply one-sequence repeated context masking (‘Repeated context masking’ section).

#### SynthID-Text settings

Unless otherwise mentioned, for all SynthID-Text experiments, we use *m* = 30 tournament layers, a Bernoulli(0.5) *g*-value distribution *f*_*g*_ (Algorithm 2) and the Bayesian scoring function (Supplementary Information section A.4).

## Online content

Any methods, additional references, Nature Portfolio reporting summaries, source data, extended data, supplementary information, acknowledgements, peer review information; details of author contributions and competing interests; and statements of data and code availability are available at 10.1038/s41586-024-08025-4.

## Supplementary information

Supplementary Information

## Figures and Tables

**Extended Data Fig. 2:**
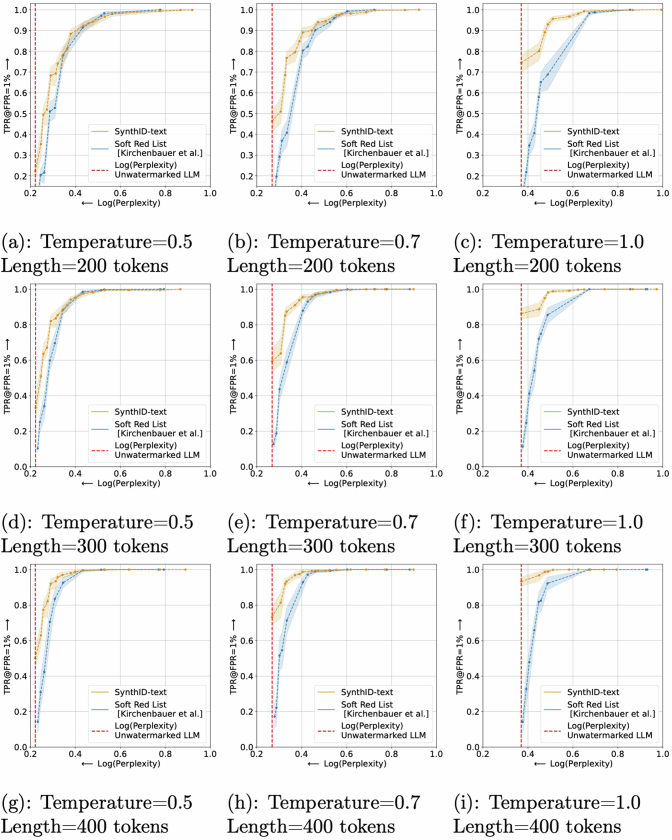
Detectability and text quality comparison of distortionary SynthID-Text and Soft Red List, for additional temperatures and text lengths. Detectability is measured by true positive rate at a fixed false positive rate of 1% (TPR@FPR=1%), and text quality is measured by log perplexity. Texts are generated by Gemma 7B-IT. See Supplementary Information section D for details. Dashed lines correspond to a bootstrap estimate (500 resamples) of the mean TPR@FPR=1%, and the shaded regions correspond to the 90% confidence interval on the mean estimate.

**Extended Data Fig. 3:**
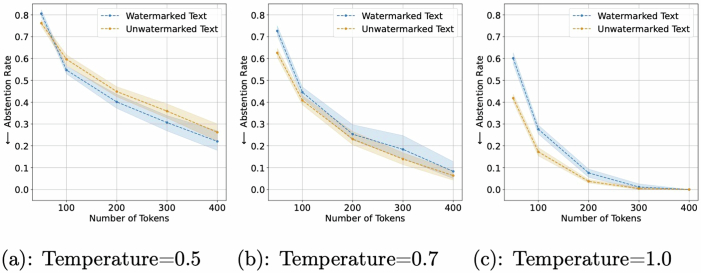
Abstention rates for non-distortionary SynthID-Text, for additional temperatures. The fraction of watermarked and unwatermarked texts abstained on by the selective prediction mechanism described in Supplementary Information section C.8, for Gemma 7B-IT. The mechanism abstains when the scoring function is uncertain, and is configured here to obtain a true positive rate of 95% and a false positive rate of 1% on the remaining texts. The abstention rate is lower for longer texts and higher temperatures. Dashed lines correspond to a bootstrap estimate (500 resamples) of the mean abstention rate on watermarked and unwatermarked texts, and the shaded regions correspond to the 90% confidence interval on the mean estimate.

**Extended Data Table 1:**
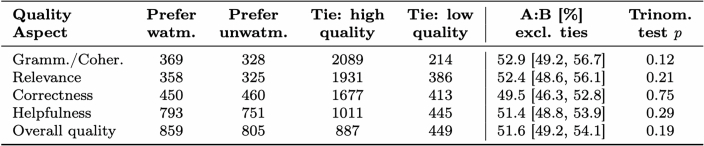
Human preference ratings for unwatermarked responses vs. responses watermarked with non-distortionary SynthID-Text

Responses are generated by Gemma 7B-IT in response to 3,000 ELI5 questions. We find no statistically significant difference in the preference for watermarked vs. unwatermarked responses. On the right, relative ratio of *prefer watermarked* vs. *prefer unwatermarked* outcomes with bootstrapped symmetric 95% confidence interval (A:B, excluding ties); and the *p*-value under a trinomial test for a symmetric distribution between watermarked and unwatermarked responses, in which ties are included and grouped as the third possible outcome. See Supplementary Information section C.4 for more details.

## Data Availability

The data from the human evaluation study described in Supplementary Information section C.4 (model responses and human annotations) is available in ref. ^7^.

## Code availability

The code to generate and detect text with SynthID-Text watermarking is available in ref. ^7^.
