# Supplementary material for: Scalable watermarking for identifying large language model outputs
Source: Nature. 2024 Oct 23;634(8035):818–23. doi: 10.1038/s41586-024-08025-4 (PMC11499265; doi:10.1038/s41586-024-08025-4)
Supplement: Supplementary file 1 — Supplementary Information [file 41586_2024_8025_MOESM1_ESM.pdf]

nature portfolio

https://doi.org/10.1038/s41586-024-08025-4

Supplementary information

Scalable watermarking for identifying large language model outputs

In the format provided by the authors and unedited

Supplementary Information

Appendix A Scoring functions

In this section we first introduce some notation, then describe several scoring functions for Tournament sampling.

A.1 *g*-value notation and masking

Our proposed scoring functions for Tournament sampling are computed from the g-values of the text, which provide the watermarking evidence. Specifically, recall that for multi-layer Tournament sampling (Methods Algorithm 2), we compute the g-values $g_1(x_t, r_t), \ldots, g_m(x_t, r_t)$ for each of the m layers. For conciseness we will write $g_{t,\ell} := g_\ell(x_t, r_t)$ to refer to these g-values.

In practice, our scoring functions do not use *all* the g-values $\{g_{t,\ell} : 1 \le t \le T, 1 \le \ell \le m\}$. To reflect the masking applied during generation (Methods Section 5.6), we make two modifications: (a) we discard the $g_{t,\ell}$ for $t = 1, \ldots, H$ due to the incomplete context window, and (b) we discard the $g_{t,\ell}$ for steps t where the context $x_{t-H}, \ldots, x_{t-1}$ appears previously in the sequence. This means that in practice, the collection of g-values used for scoring is $\{g_{t,\ell} : t \in \hat{T}, 1 \le \ell \le m\}$ for some subset $\hat{T} \subseteq \{1, \ldots, T\}$. For notational simplicity, we will write the following scoring functions assuming we use all the g-values; to obtain the masked version simply replace sums over $t = 1, \ldots, T$ with sums over $t \in \hat{T}$ and replace T with $|\hat{T}|$.

A.2 Mean

Tournament sampling works by returning tokens that are more likely to have high g-values. Thus, the simplest scoring function is simply to take the mean g-value across all tokens in the text and all layers:

$$\mathrm{MeanScore}(x) := \frac{1}{mT} \sum_{t=1}^{T} \sum_{\ell=1}^{m} g_{t,\ell}. \tag{A1}$$

For the Bernoulli(0.5) or Uniform[0,1] g-value distributions used in our experiments, the MeanScore of a text is between 0 and 1, with an expected score of 0.5 for unwatermarked text and a larger score expected for watermarked text.

A.2.1 Weighted Mean

In Supplementary Appendix H.4 we show that the amount of watermarking evidence contributed by each layer decreases as more layers are added. This motivates the Weighted Mean variant, which applies weights $\alpha_1 \ge \cdots \ge \alpha_m \ge 0$, where $\sum_{\ell=1}^{m} \alpha_\ell = m$, to the sum of the g-values:

$$\mathrm{WeightedMeanScore}(x, \alpha) := \frac{1}{mT} \sum_{t=1}^{T} \sum_{\ell=1}^{m} \alpha_\ell \, g_{t,\ell}. \tag{A2}$$

We find that for a simple linearly decreasing choice of α, WeightedMeanScore generally outperforms MeanScore. Specifically, we use $\alpha_1 = \kappa$, $\alpha_2 = \kappa - \frac{\kappa-\mu}{m-1}$, $\alpha_3 = \kappa - 2\frac{\kappa-\mu}{m-1}, \ldots, \alpha_m = \mu$ with $\kappa = 10, \mu = 1$, then renormalised so $\sum_{\ell=1}^{m} \alpha_\ell = m$.

A.3 Frequentist

In some cases it may be desirable to perform a hypothesis test against the null hypothesis that the text is unwatermarked; this has the advantage of providing a p-value which allows us to exactly control the false positive rate. Under the null hypothesis, each $g_{t,\ell}$ follows the g-value distribution f_g (Methods Definition 3); furthermore if we apply repeated context masking (Supplementary Appendix A.1) then the $g_{t,\ell}$ are independent. This allows us to compute[1] the p-value for the sum $\sum_{t=1}^{T} \sum_{\ell=1}^{m} g_{t,\ell}$:

$$p\text{-value} = 1 - \mathrm{CDF}_{\mathrm{Binomial}(mT,0.5)}\left(\left[\sum_{t=1}^{T}\sum_{\ell=1}^{m} g_{t,\ell}\right] - 1\right) \quad \text{if } f_g = \mathrm{Ber}(0.5) \tag{A3}$$

$$p\text{-value} = 1 - \mathrm{CDF}_{\text{Irwin-Hall}(mT)}\left(\sum_{t=1}^{T}\sum_{\ell=1}^{m} g_{t,\ell}\right) \quad \text{if } f_g = \mathrm{Unif}[0,1]. \tag{A4}$$

We define FrequentistScore(x) to be the negative p-value and classify texts as watermarked if the score exceeds a threshold.

When scoring a corpus of texts that are all exactly the same length, the FrequentistScore is equivalent to the MeanScore (i.e., they should produce the same detectability metrics); the WeightedFrequentistScore that follows is similarly equivalent to the WeightedMeanScore. For simplicity therefore, in our experiments we use the Mean versions instead of the Frequentist versions of the scores.

A.3.1 Weighted Frequentist

Similarly to the Weighted Mean score, we can weight the evidence of the earlier layers more strongly than later layers by applying weights $\alpha_1 \geq \ldots, \geq \alpha_m \geq 0$ where $\sum_{\ell=1}^{m} \alpha_\ell = m$. For this hypothesis test we use a Z-test. First, we compute the mean μ and variance σ^2 of the weighted sum on a single step, $\sum_{\ell=1}^{m} \alpha_\ell \, g_{t,\ell}$, under the null hypothesis; for example:

$$\mu = \frac{m}{2}, \quad \sigma^2 = \frac{1}{4}\sum_{\ell=1}^{m} \alpha_\ell^2 \qquad \text{if } f_g = \mathrm{Ber}(0.5)$$

$$\mu = \frac{m}{2}, \quad \sigma^2 = \frac{1}{12}\sum_{\ell=1}^{m} \alpha_\ell^2 \qquad \text{if } f_g = \mathrm{Unif(0,1)}.$$

[1]If the Binomial or Irwin-Hall CDFs are not easily computable, we can instead use the CDF of the normal approximation; this is equivalent to the method in Supplementary Appendix A.3.1 using all weights equal to 1.

It follows that the mean of these weighted sums across all steps, $\frac{1}{T}\sum_{t=1}^{T}\sum_{\ell=1}^{m}\alpha_\ell\, g_{t,\ell}$, is approximated by the Normal(μ, $\frac{\sigma^2}{T}$) distribution. Thus we can compute a p-value:

$$p\text{-value} = 1 - \mathrm{CDF}_{\mathrm{Normal}(\mu,\frac{\sigma^2}{T})}\left(\frac{1}{T}\sum_{t=1}^{T}\sum_{\ell=1}^{m}\alpha_\ell\, g_{t,\ell}\right). \tag{A5}$$

A.4 Bayesian

In this section we present a two-sided approach that (unlike the one-sided Frequentist approach which only assumes the unwatermarked g-value distribution) also uses knowledge of the watermarked g-value distribution, which is learned from data. Assuming we have access to a representative set of labeled watermarked and unwatermarked samples for training, this approach is able to offer more information than the Frequentist approach, by considering how g-values are distributed for *both* hypotheses.

Formally, we have two hypotheses: watermarked (w) or unwatermarked ($\neg w$). We treat the watermarking hypothesis as a latent variable and the g-values $\{g_{t,\ell}\}_{1\le t\le T, 1\le \ell\le m}$ as the observed evidence. The *prior* $P(w)$ is the probability *a priori* that a piece of text is watermarked; it can be learned empirically or set to reflect a belief about the watermarked base rate. The *posterior* $P(w|g)$ is the probability that the text is watermarked, given its g-values. The *likelihoods* $P(g|\neg w)$ and $P(g|w)$ are the probabilities of observing these g-values, in unwatermarked text or in watermarked text respectively. Bringing these together, we can compute the *log posterior odds*:

$$\begin{aligned}\mathrm{LogPosteriorOdds}(x) &= \log\left(\frac{P(w|g)}{P(\neg w|g)}\right)\\ &= \log\left(\frac{P(g|w)P(w)}{P(g|\neg w)P(\neg w)}\right)\\ &= \log P(g|w) - \log P(g|\neg w) + \log P(w) - \log\left(1 - P(w)\right).\end{aligned}$$

We define the BayesianScore as the the watermarked posterior $P(w|g)$, i.e., the probability that the text x is watermarked, given its g-values. This can be computed from the log posterior odds like so:

$$\begin{aligned}\mathrm{BayesianScore}(x) &:= P(w|g)\\ &= \sigma\left[\mathrm{LogPosteriorOdds}(x)\right]\\ &= \sigma\left[\log P(g|w) - \log P(g|\neg w) + \log P(w) - \log\left(1 - P(w)\right)\right]\end{aligned} \tag{A6}$$

where $\sigma(\cdot)$ is the sigmoid function. To use the BayesianScore for Tournament sampling, we just need to determine the likelihoods $P(g|\neg w)$ and $P(g|w)$:

Theorem 6 (Bayesian likelihoods for multi-layer Tournament sampling)**.** *For multi-layer Tournament sampling, the likelihoods can be factorized as:*

$$P(g|\neg w) = \prod_{t=1}^{T}\prod_{\ell=1}^{m} f_g(g_{t,\ell}) \tag{A7}$$

$$P(g|w) = \prod_{t=1}^{T}\prod_{\ell=1}^{m}\sum_{c=1}^{N} P(g_{t,\ell}|\psi_{t,\ell}=c)P(\psi_{t,\ell}=c|g_{t,<\ell}) \tag{A8}$$

where $\psi_{t,\ell}$ is a random variable representing the number of unique tokens in a tournament match on layer ℓ, on timestep t. Furthermore, $P(g_{t,\ell}|\psi_{t,\ell}=c)$ can be written in terms of the g-value distribution f_g and F_g (Methods Definition 3):

$$P(g_{t,\ell}|\psi_{t,\ell}=c) = \begin{cases} cF_g(g_{t,\ell})^{c-1} f_g(g_{t,\ell}) & \text{if } f_g \text{ is continuous} \\ F_g(g_{t,\ell})^{c} - [F_g(g_{t,\ell}) - f_g(g_{t,\ell})]^{c} & \text{if } f_g \text{ is discrete.} \end{cases} \tag{A9}$$

Proof. See Supplementary Appendix K.1. □

The factorization in Theorem 6 is based on two intuitions. First, the distribution of a watermarked g-value $g_{t,\ell}$ can be determined exactly if we know the number of unique candidates $\psi_{t,\ell}$ (it is given in Equation (A9)). Second, the number of unique samples $\psi_{t,\ell}$ is dependent on the amount of entropy in the distribution on layer ℓ; and this can be predicted as a function of the lower-level g-values $g_{t,<\ell}$ because on a high-entropy timestep t, the g-values $g_{t,<\ell}$ are likely to be larger. Accordingly, we model the probabilities $P(\psi_{t,\ell}=c|g_{t,<\ell})$ as learned functions of $g_{t,<\ell}$. Specifically, for experiments with $N=2$ samples, we use a logistic regression model to learn $P(\psi_{t,\ell}=2|g_{t,<\ell})$:

$$P(\psi_{t,\ell}=2|g_{t,<\ell}) = \sigma\left(\beta_\ell + \sum_{j=1}^{\ell-1}\delta_{\ell,j}\,g_{t,j}\right), \tag{A10}$$

where $\sigma(\cdot)$ is the sigmoid function, $\beta_\ell \in \mathbb{R}$ is the bias parameter for layer ℓ, and the weight $\delta_{\ell,j} \in \mathbb{R}$ refers to the effect of $g_{t,j}$ on the probability that $\psi_{t,\ell}=2$. As $N=2$, we can then set $P(\psi_{t,\ell}=1|g_{t,<\ell}) = 1 - P(\psi_{t,\ell}=2|g_{t,<\ell})$.

For the non-distortionary configurations used in this work, BayesianScore has a simple form, which follows directly from Theorem 6:

Theorem 7 (BayesianScore for $N=2$, Bernoulli(0.5) g-value distribution)**.** *If $N=2$ and $f_g =$ Bernoulli(0.5), then:*

$$\textit{BayesianScore}(x) = \sigma\left(\sum_{t=1}^{T}\sum_{\ell=1}^{m}\left[P(\psi_{t,\ell}=1|g_{t,<\ell}) + (g_{t,\ell}+0.5)P(\psi_{t,\ell}=2|g_{t,<\ell})\right]\right.$$

$$+ \log P(w) - \log(1 - P(w))\Bigg).$$

Proof. Follows from substituting $f_g(z) = 0.5$ and $F_g(z) = 0.5 + 0.5z$ into Theorem 6 and Equation (A6).

Theorem 8 (BayesianScore for $N = 2$, Uniform g-value distribution)**.** *If* $N = 2$ *and* $f_g = \textit{Uniform}[0, 1]$*, then:*

$$\textit{BayesianScore}(x) = \sigma\Bigg(\sum_{t=1}^{T}\sum_{\ell=1}^{m}\left[P(\psi_{t,\ell} = 1 | g_{t,<\ell}) + 2\, g_{t,\ell}\, P(\psi_{t,\ell} = 2 | g_{t,<\ell})\right]$$

$$+ \log P(w) - \log(1 - P(w))\Bigg).$$

Proof. Follows from substituting $f_g(z) = 1$ and $F_g(z) = z$ into Theorem 6 and Equation (A6).

Appendix B Related work: Generative watermarking

In this section we discuss other generative watermarks; we divide our discussion into sampling algorithms, random seed generators, scoring functions, and other techniques.

B.1 Sampling algorithms

In this section we describe existing sampling algorithms (Methods Definition 5) which are alternatives to Tournament sampling. Our two baselines are Gumbel sampling and Soft Red List, which we choose both for their prevalence in the literature and their high performance relative to other methods [21, 37]. We give detailed descriptions of our baselines, then discuss some other sampling algorithms.

B.1.1 Baseline: Gumbel (aka Exponential minimum) sampling

In general, the *Gumbel trick* [38] is a method to take a sample x^* from any categorical probability distribution $p(x_1), \ldots, p(x_V)$ by adding i.i.d. samples $G_1, \ldots, G_V$ from the Gumbel(0,1) distribution to the log probabilities:

$$x^* := \arg\max_{1 \le i \le V} \left[\log p(x_i) + G_i\right].$$

It can be shown that $\mathbb{P}(x^* = x_i) = p(x_i)$ for all i. It is also true that the Gumbel(0,1) distribution is equivalent to $-\log(-\log(U))$ if $U \sim \text{Uniform}[0, 1]$. Therefore, an equivalent formulation is to take i.i.d. samples $U_1, \ldots, U_V$ from the Uniform[0,1]

distribution, then choose x^* as follows, which can be written in several equivalent ways:

$$
\begin{aligned}
x^* &:= \underset{1\le i\le V}{\arg\max}\,[\log p(x_i) - \log(-\log(U_i))] \\
&= \underset{1\le i\le V}{\arg\max}\left[\log\left(-\frac{p(x_i)}{\log(U_i)}\right)\right] \\
&= \underset{1\le i\le V}{\arg\min}\left[-\frac{\log(U_i)}{p(x_i)}\right]. && \text{(Kuditipudi et al. [24] formulation)} \quad \text{(B11)} \\
&= \underset{1\le i\le V}{\arg\max}\left[U_i^{1/p(x_i)}\right]. && \text{(Aaronson and Kirchner [22] formulation)} \quad \text{(B12)}
\end{aligned}
$$

Aaronson and Kirchner [22] and Kuditipudi et al. [24] propose this method as a sampling algorithm, using $p := p_{\text{LM}}(\cdot|x_{<t})$ in the equations above; Kuditipudi et al. [24] call the method *exponential minimum sampling*. In the terminology of this paper, the Gumbel sampling algorithm for watermarking can be implemented by taking the random seed r_t and setting each U_i to be a pseudorandom uniform g-value $U_i := g(x_i, r_t)$ by setting the g-value distribution $f_g = \text{Uniform}[0, 1]$, as described in Methods Section 5.4.

Gumbel sampling is a non-distortionary (Definition 16) deterministic sampling algorithm that produces tokens with higher $g(\cdot, r_t)$ values. As it is deterministic, it provides no entropy to resample from; this is a disadvantage compared to probabilistic sampling algorithms like Tournament sampling.

To detect the Gumbel watermark, we take a text $x_1, \ldots, x_T$ and compute its g-values $g(x_1, r_1), \ldots, g(x_T, r_T)$ which we denote $g_1, \ldots, g_T$ for short; these are independently Uniform[0,1] distributed if x is unwatermarked and likely to be higher if x is watermarked. Aaronson and Kirchner [22] propose the following scoring function:

$$\text{LogScore}(x) := -\sum_{t=1}^{T} \log\left(1 - g_t\right). \tag{B13}$$

Another possible scoring function is $\text{MeanScore}(x) = \frac{1}{T}\sum_{t=1}^{T} g_t$, similar to Equation (A1) for Tournament sampling. To provide a fair comparison to the Bayesian scoring function for Tournament sampling (Supplementary Appendix A.4), we also develop a learned Bayesian scoring function for the Gumbel watermark. Here, we use the BayesianScore defined in Equation (A6), and approximate $P(g|w)$ with a simple multi-layer perceptron (MLP). Specifically, $P(g|w) = \prod_{t=1}^{T} P(g_t|w)$ where $P(g_t|w)$ is computed by the MLP, which takes just a single number g_t as input.

B.1.2 Baseline: Soft Red List sampling

We use the recommended Soft Red List sampling algorithm from Kirchenbauer et al. [23], in which a proportion $\gamma \in (0, 1)$ of the vocabulary is green, the rest are red, and

a constant $\delta > 0$ is added to all logits on the green list. Described in the terminology of Methdos Section 5.4, this can be implemented by taking the random seed r_t and computing a g-value $g(x_t, r_t)$ for each token $x_t \in V$ using the g-value distribution $f_g = \text{Bernoulli}(\gamma)$, then sampling an output token x^* as follows:

$$\begin{aligned}
\text{logit}(x_t) &:= \log p_{\text{LM}}(x_t|x_{<t}) + \delta g(x_t, r_t) && \text{for all } x_t \in V \\
p_{\text{wm}}(x_t) &:= \frac{\exp(\text{logit}(x_t))}{\sum_{x'_t \in V} \exp(\text{logit}(x'_t))} && \text{for all } x_t \in V \\
x^* &\sim p_{\text{wm}}.
\end{aligned}$$

This is a distortionary (Definition 16) probabilistic sampling algorithm that produces tokens with higher $g(\cdot, r_t)$ values. As a distortionary sampling algorithm, it has been shown to affect text quality (in particular increasing perplexity), especially when δ is large or γ is small [23, 24].

To detect the Soft Red List watermark, we take a text $x_1, \ldots, x_T$ and compute its g-values $g(x_1, r_1), \ldots, g(x_T, r_T)$ which we denote $g_1, \ldots, g_T$ for short; these are independently Bernoulli(γ) distributed if x is unwatermarked and likely to be higher if x is watermarked. We can apply $\text{MeanScore}(x) = \frac{1}{T}\sum_{t=1}^{T} g_t$, similarly to Equation (A1). Alternatively, we can apply a Frequentist scoring function, similar to the method used by Kirchenbauer et al. [23]:

$$p\text{-value} = 1 - \text{CDF}_{\text{Binomial}(T,\gamma)}\left(\left[\sum_{t=1}^{T} g_t\right] - 1\right). \tag{B14}$$

When all texts in the corpus are the same length, MeanScore is equivalent to FrequentistScore (see Supplementary Appendix A.3) and so in our experiments we use MeanScore to match our methodology for Tournament sampling.

B.1.3 Other sampling algorithms

Here we mention a few more sampling algorithms, that we do not include as baselines:

- Inverse Transform Sampling (ITS) is a simple deterministic non-distortionary watermarking sampling algorithm, however it has been shown to have lower detectability than Gumbel sampling [24, 25], so we do not include it in our experimental baselines.
- Zhao et al. [39] propose a probabilistic distortionary sampling algorithm GINSEW, which involves applying a sinusoidal perturbation to the LLM probability distribution. For the distortionary category, we focus our comparison on the more widely-known Soft Red List sampling algorithm; to our knowledge GINSEW has not been empirically compared to Soft Red List so its relative performance is unknown.
- Hopper et al. [40] propose a watermarking sampling algorithm that is equivalent to the special case of Tournament sampling with $m = 1$ layer, $N = 2$ samples, and a Bernoulli(0.5) g-value distribution; however, in its generality the Tournament sampling algorithm presented in this work is novel.

B.2 Random seed generators

In this work we use the sliding window random seed generator (Methods Section 5.3). As noted in the literature [24, 25], the sliding window method can introduce sequence-level distortion (e.g., repetitive loops in text) when the same context (and thus the same random seed) is used repeatedly. We avoid this problem by applying repeated context masking (Methods Section 5.6); however, there are other ways to designing a random seed generator while reducing the likelihood of repeatedly applying the same random seed.

Kuditipudi et al. [24] propose using a cycling sequence of random seeds – when paired with a distortion-free sampling algorithm, this method is *single-sequence non-distortionary* (Definition 20) if and only if the seed sequence is longer than the text length. However, meeting this criterion can be tricky in practice, as the maximum text length may be quite long, and increasing the seed sequence length reduces the overall watermark detectability as it requires searching for the correct alignment of the text and the seed sequence during detection. For this reason we do not use the cycling sequence method even though it is compatible with Tournament sampling; instead we choose a method (repeated context masking) that can give precise single-sequence non-distortion guarantees (Theorem 21) regardless of text length.

Another approach is proposed by Christ et al. [25]: like the sliding window method, they use recent text context to generate random seeds; however the algorithm adapts to the entropy in the text to guarantee that the likelihood of repeated seeds is low. While this approach (when paired with a non-distortionary sampling algorithm) meets a strong notion of cryptographic indistinguishability, it is also less robust to edits, more computationally expensive to detect, and has lower watermarking strength. However, if this type of indistinguishability is desired, the Tournament sampling algorithm can be combined with this entropy-adaptive method.

While the work discussed above focuses on avoiding random seed re-use in order to minimize distortion, Zhao et al. [41] take an opposite approach, using the same random seed on every step. They pair this random seed generator with the Soft Red List sampling algorithm and show that this 'Unigram' approach is more robust to edits than a sliding window approach. However, this robustness comes at the cost of decreased text quality and watermark security.

B.3 Scoring functions

In this work we focus on designing and evaluating scoring functions (Supplementary Appendix A) that score a whole text x, optimizing performance for the case that x is either completely unwatermarked, or x is the full unaltered text generated by the watermarked LLM. However, it can be useful to consider other cases, such as when x contains a mix of watermarked and unwatermarked text, or when x is a watermarked text that has been edited. Our scoring functions still work in these scenarios, but their performance reduces as the amount of original watermarked text decreases (Supplementary Appendix C.6).

Existing work has proposed alternative scoring functions that perform better under these circumstances. Kuditipudi et al. [24] propose a block-based scoring function that,

for some specified block size k, searches through the text for the length-k block of text with strongest watermarking evidence. Such a scoring function could be used with Tournament sampling; the scoring functions presented in Supplementary Appendix A could be modified to operate over blocks of text. Kuditipudi et al. [24] also propose a scoring function that is designed to be robust to edits; this scoring function searches for the minimum-cost alignment between the text and the watermark, accounting for edits with a Levenshtein cost. While both these scoring functions have the advantage of performing better when the text contains watermarked sub-passages, or when the text has been edited, their overall statistical power decreases in the case that the entire text is watermarked and unedited.

B.4 Additional techniques

Giboulot and Teddy [37] propose a generative watermarking approach that does not fit into the framework presented thus far – one samples multiple texts from the original unwatermarked LLM, then chooses the text that scores most highly according to a scoring function. While Giboulot and Teddy [37] show that this approach provides a good detectability-robustness-quality tradeoff, it substantially increases the computational cost of text generation. As computational cost is one of the most important priorities in a production system, we do not experiment with this method.

In the category of distortionary sampling algorithms, Wouters [42] propose a method to reduce the distortion by applying the watermark only on steps when the expected perplexity increase is sufficiently low. This method could be applied to any distortionary sampling algorithm such as Soft Red List or distortionary Tournament sampling; however it is important to note that even if the perplexity is equal or lower than the unwatermarked LLM, the method is still distortionary.

Appendix C Non-Distortionary watermarking experiments

In this section we present further experiments with non-distortionary SynthID-Text and the Gumbel sampling baseline.

C.1 Tournament depth and scoring functions

In this section we present our experiments comparing the performance of the different scoring functions for (non-distortionary) Tournament sampling (Supplementary Appendix A), and their interaction with Tournament depth (i.e., number of layers).

Bayesian learning procedure

To learn the Bayesian scoring function (Supplementary Appendix A.4), the parameters are optimized by minimizing the cross-entropy loss between the predictions and the labels (watermarked or unwatermarked) using gradient descent. We use 30% of the 10,000 watermarked and 10,000 unwatermarked training samples for cross-validation, and the rest for learning the parameters. During cross-validation, we choose the parameters maximizing TPR@FPR=1% for texts of length 200 tokens on the validation set.

We use a learning rate of 1×10^{-3}, a mini-batch size of 64, and 50 epochs. Empirically we find that truncating the watermarked sequences to 200 tokens during training to synthetically increase the difficulty of the classification task improves the generalization performance. During testing, the full length of the text available to use is utilized without any truncation.

Weighted Mean learning procedure

For the WeightedMean scoring function (Supplementary Appendix A.2.1), we find that the performance on the training/validation set is not sensitive to the choice of weights and we simply use a set of weights decaying linearly from 10.0 to 1.0 across the layers.

Results

In Figure C1 we see that the Mean and WeightedMean scoring functions peak at certain depths, with detectability degrading as the depth is further increased. This is due to the fact that earlier layers contain more watermarking information than later layers (see Supplementary Appendix H.5). By contrast the Bayesian scoring function provides better performance than Mean and WeightedMean across all temperatures and depths. In particular, the Bayesian performance plateaus but does not decrease as we add more layers; this is because the Bayesian scoring function is able to learn to reduce the contributions from the later layers (see Supplementary Appendix A.4). The Bayesian scoring function also benefits from being able to model the expected g-values for the later layers based on the g-values from the earlier layers. The g-values are used by the scoring function to adjust $p\,(g|w)$ for the later layers, leading to further improved detection performance. The WeightedMean and the Mean scoring functions are not able to adapt in a similar manner, resulting in their weaker performance. As we typically see diminishing returns beyond 30 tournament layers, for all experiments with non-distortionary SynthID-Text (including speculative sampling) we use 30 tournament layers.

C.2 Gumbel sampling: scoring functions

For Gumbel sampling, we compare the LogScore $\log(1-g)$ scoring function and the learned Bayesian scoring function described in Supplementary Appendix B.1.1.

Bayesian learning procedure

As described in Supplementary Appendix B.1.1, we train a MLP-based Bayesian scoring function for Gumbel sampling. Similar to the training procedure for the Tournament Bayesian scoring function, we use 30% of the 10,000 watermarked and 10,000 unwatermarked training samples for cross-validation, and the rest for learning the parameters. During cross-validation, as before, we choose the parameters maximizing TPR@FPR=1% for texts of length 200 tokens on the validation set. We use a learning rate of 1×10^{-3}, a mini-batch size of 64, and 50 epochs. We run a hyperparameter search where we vary the the number of hidden layers in the MLP over the set $\{1, 2\}$, the number of hidden neurons per layer is varied over the set $\{3, 5, 7, 10, 20, 50, 100\}$, the learning rate is varied over `logspace`(-3, -1, num=4), i.e., we try four equidistant

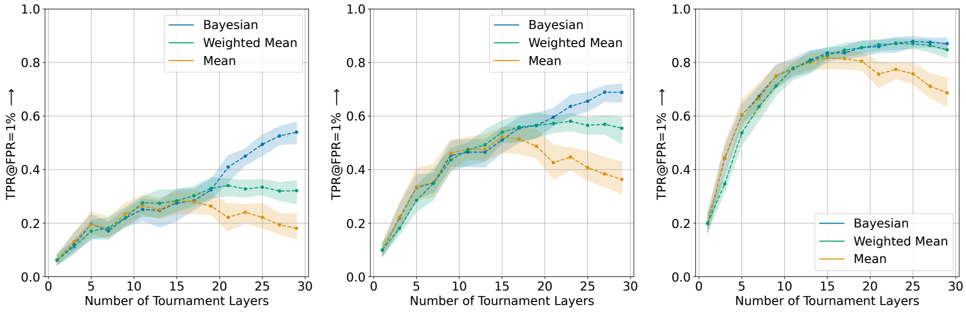

(a) Temperature=0.5 (b) Temperature=0.7 (c) Temperature=1.0

Fig. C1: Effect of number of tournament layers, and choice of scoring function on the detectability of text generated with non-distortionary SynthID-Text (all texts are 200 tokens). Texts are generated from `Gemma` 7B-IT with three different model temperatures. Detectability is measured by true positive rate at a false positive rate of 1% (TPR@FPR=1%). Dashed lines correspond to a bootstrap estimate of the mean TPR@FPR=1%, and shaded regions correspond to the 90% confidence interval on the mean estimate.

values for the learning rate on the log-scale, ranging between 10^{-3} to 10^{-1}. We vary the length for truncating the watermarked responses over 100, 200, 300, and 400 tokens. We train MLPs across all of these parameter settings, and select the one performing the best on the cross-validation set based on TPR@FPR=1% for texts of length 200 tokens. These parameters are then evaluated on the held-out test set without any truncation.

Results

We see in Figure C2 that the two scoring functions have very similar performance, with the LogScore $\log(1-g)$ performing slightly better in average, with the improvement in most settings not being statistically significant. Unlike for Tournament sampling, the learned scoring function does not improve performance; we conjecture this may be because the function being learned $\mathbb{P}(g|w)$, a mixture of beta distributions [43], is more complex for Gumbel sampling than that for Tournament sampling, where $\mathbb{P}(g|w)$ for each layer is a Bernoulli distribution. Additionally, the scoring function for Gumbel sampling is not able to benefit from information provided in earlier layers. Given the comparable performance of the two detection strategies, we use the $\log(1-g)$ scoring function as the baseline throughout the paper.

C.3 Diversity effects

We also measure the diversity effects of the two watermarks. As discussed in Supplementary Appendix G.3, our two non-distortionary baselines are *single-sequence non-distortionary*, meaning they do not affect the diversity within a single text (e.g., they do not cause repeating loops in text). However, they do reduce the diversity

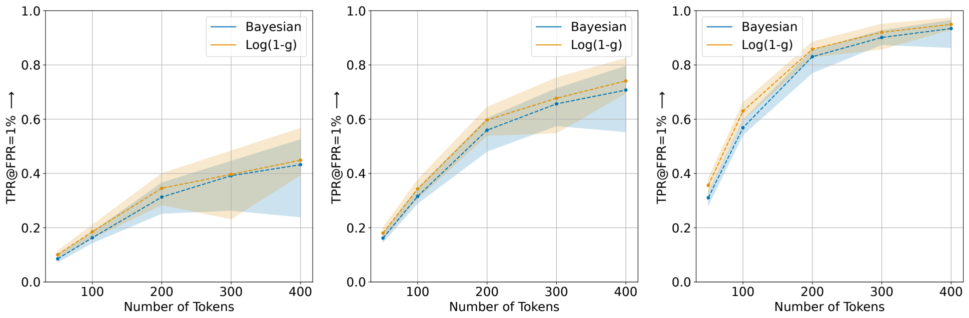

(a) Temperature=0.5 (b) Temperature=0.7 (c) Temperature=1.0

Fig. C2: Effect of choice of scoring function on the detectability of text generated with Gumbel sampling. Texts are generated from `Gemma` 7B-IT with three different model temperatures. Detectability is measured by true positive rate at a false positive rate of 1% (TPR@FPR=1%). Dashed lines correspond to a bootstrap estimate of the mean TPR@FPR=1%, and shaded regions correspond to the 90% confidence interval on the mean estimate.

across multiple responses; in particular, if we sample multiple responses to the same prompt, they are more likely to be similar to each other if they are watermarked, than if they are from the unwatermarked model. We measure this inter-response diversity empirically by measuring the Self-BLEU similarity [44] between pairs of responses to the same prompt.

To mitigate the inter-response diversity problem, Aaronson [45] suggest turning off the watermark on a fraction of all timesteps, thus increasing the chance that the texts diverge; however this reduces watermark detectability. We can achieve a similar diversity/detectability trade-off with SynthID-Text simply by varying the number of tournament layers; more layers provides stronger detectability and lower diversity, while fewer layers provides weaker detectability and higher diversity. Extended Data Figure 4 shows that the diversity/detectability trade-off is more favourable for SynthID-Text than for Gumbel sampling. For this experiment we generated two responses to each prompt using `Gemma` 7B-IT, and measured the pairwise Self-BLEU between each pair of responses to the same prompt. We varied the number of Tournament layers from 1 to 30, and the Gumbel watermark probability from 0.1 to 1.0.

C.4 Human preference test

In this section we provide details of the human preference test comparing non-distortionary SynthID-Text to unwatermarked responses. For this experiment we sample both a watermarked and an unwatermarked response to 3,000 `ELI5` [30] questions from a `Gemma` 7B-IT model with a temperature of 0.7. We present the two responses side-by-side, randomly labelled A and B, alongside the `ELI5` question, to human raters on the Prolific platform. Raters are presented with five questions:

- (Relevance) Which response is more relevant to the question?
- (Correctness) To the extent you can tell, which response is more correct?
- (Helpfulness) Which response do you find more helpful overall?
- (Grammaticality/coherence) Which response is better in terms of grammatical correctness, comprehensibility and coherence?
- (Overall quality) Taking into account the overall answer relevance, correctness, helpfulness, as well as grammatical correctness, which of the two responses is of higher quality?

For each of these five questions, raters choose one of the following options: *Response A*, *Response B*, *Both are low quality*, or *Both are high quality*.

To measure the rater agreement, we ran a pilot study over 100 examples, annotated fourfold, and measured the pairwise rater agreement over all paired non-tie ratings. We find agreements of 73.4% (*relevance*), 73.6% (*correctness*), 67.8% (*helpfulness*), 75.9% (*grammaticality / coherence*), and 63.7% (*overall quality*); broadly in line with previous work [46].

Extended Data Table 1 shows the results. For our analyses we consider the null hypothesis to be that there is no difference in the response quality between watermarked vs. unwatermarked responses. In our first analysis, we only consider the non-tie cases (i.e. where the rater expressed a preference for one of the two responses), and calculate the fraction of cases preferring the watermarked response vs. the cases preferring the unwatermarked response. We calculate symmetric 95% confidence intervals using bootstrap resampling of the 3,000 collected responses. For all of the five questions, 50% (the value expected under the null hypothesis) is within this confidence interval. In our second analysis, we include the neutral ratings by grouping the *Both are low quality* and *Both are high quality* ratings into a *tie* label. Similarly here, none of the p-values under a trinomial test reaches statistical significance. We conclude that for all five ratings, the data collected does not provide sufficient evidence to reject the null hypothesis of no difference between watermarked and unwatermarked responses.

C.5 Automatic quality evaluations

We provide results of several automatic quality evaluations to demonstrate that non-distortionary SynthID-Text is quality-neutral:

- Table C1 shows that non-distortionary SynthID-Text and the Gumbel baseline both have no effect on perplexity, for a variety of models and temperatures.
- Table C2 shows that non-distortionary SynthID-Text performs equally well as the equivalent unwatermarked model on a collection of automatic benchmarks assessing coding ability [47, 48], language modeling [49], mathematics [50, 51], and general abilities of foundation models [52, 53], for Gemma 2B-PT and 7B-PT. Note that these experiments use 20 tournament layers, rather than 30. We find no preference between responses watermarked with non-distortionary SynthID-Text, and unwatermarked responses.

Model	Temp.	Unwatermarked	Non-distort. SynthID-Text	Gumbel
2B-IT `Gemma`	1.0	1.720 [1.709, 1.729]	1.726 [1.716, 1.740]	1.715 [1.699, 1.732]
	0.7	1.509 [1.499, 1.515]	1.500 [1.496, 1.506]	1.487 [1.472, 1.499]
	0.5	1.401 [1.395, 1.407]	1.411 [1.407, 1.416]	1.395 [1.387, 1.407]
7B-IT `Gemma`	1.0	1.464 [1.447, 1.479]	1.451 [1.444, 1.459]	1.449 [1.441, 1.454]
	0.7	1.307 [1.304, 1.311]	1.306 [1.303, 1.310]	1.301 [1.292, 1.313]
	0.5	1.246 [1.242, 1.250]	1.241 [1.236, 1.249]	1.247 [1.241, 1.253]
7B-IT `Mistral`	1.0	1.408 [1.399, 1.418]	1.402 [1.393, 1.413]	1.399 [1.393, 1.405]
	0.7	1.269 [1.263, 1.276]	1.266 [1.262, 1.270]	1.268 [1.261, 1.273]
	0.5	1.218 [1.211, 1.222]	1.205 [1.200, 1.209]	1.203 [1.196, 1.210]

Table C1: Mean LLM perplexity [54] for different models and temperatures, for unwatermarked text and text watermarked with non-distortionary SynthID-Text and with Gumbel sampling. Each result is given with a 90% confidence interval based on bootstrapping. For these non-distortionary watermarks, there is no change to perplexity. The perplexity of the generated texts with and without watermarking is measured with respect to the probabilities provided by the underlying LLM.

C.6 Detectability under perturbation

We evaluate the detectability of (non-distortionary) SynthID-Text after the watermarked text has been perturbed via (a) random word deletion and (b) LLM paraphrasing. First, we generate watermarked texts using the `Gemma` 2B-IT and 7B-IT models prompted with 3,000 prompts from the `ELI5` dataset [30]. For random word deletion, we randomly delete either 20% or 50% of words (defined by space separation). For LLM paraphrasing, we prompt `Gemini Ultra` with *'Paraphrase the following article, while retaining the same semantic meaning, without losing any details. Please paraphrase sentence by sentence. Don't summarize only.*`\n` *Original: {query}*`\n`*'* and enforce the output sample to start with *"Paraphrase:"*. Some paraphrasing examples are shown in Table C3 (bottom).

Figure C3 shows the results. Like other generative watermarks, SynthID-Text provides some robustness to edits – i.e., editing the text weakens detectability, but the watermark can still be detected with high accuracy if the text is sufficiently long. The paraphrasing attack is quite strong, especially if we use a strong paraphrasing model like `Gemini Ultra` and obtain a thoroughly paraphrased text that changes most of the phrasing of the text.

Benchmark (type)		Metric ↑	Unwatermarked		Non-distort. SynthID-Text	
			2B-PT	7B-PT	2B-PT	7B-PT
`MMLU` (lang. modeling)	[49]	5-shot, top-1	32.42 [31.73%, 33.03%]	57.73 [57.05%, 58.38%]	32.9 [32.22%, 33.55%]	58.25 [57.6%, 58.97%]
`HumanEval` (coding)	[47]	pass@1	14.02 [9.76%, 18.9%]	26.22 [20.73%, 31.71%]	11.59 [7.32%, 15.24%]	25.61 [20.12%, 31.1%]
`MBPP` (coding)	[48]	3-shot	19.4 [16.6%, 22.4%]	34.4 [30.8%, 37.8%]	20.6 [17.8%, 23.8%]	37.2 [33.6%, 41.0%]
`GSM8K` (maths)	[50]	accuracy (all samples)	9.17 [8.72%, 9.61%]	38.26 [37.48%, 39.01%]	9.49 [9.0%, 9.96%]	39.14 [38.38%, 39.89%]
`MATH` (maths)	[51]	4-shot	7.28 [6.72%, 7.84%]	15.72 [14.82%, 16.56%]	7.2 [6.64%, 7.8%]	15.64 [14.82%, 16.46%]
`AGIEval` (general)	[52]	–	24.02 [22.39%, 25.43%]	42.44 [40.64%, 44.19%]	24.1 [22.69%, 25.43%]	42.44 [40.85%, 44.06%]
`BBH` (general)	[53]	–	31.92 [30.98%, 32.81%]	53.34 [52.39%, 54.35%]	31.45 [30.5%, 32.35%]	53.6 [52.59%, 54.68%]

Table C2: Comparing the performance of unwatermarked and non-distortionary SynthID-Text responses from `Gemma` 2B-PT and 7B-PT, across a range of automated LLM performance benchmarks. Each metric is given with a 90% confidence interval. Non-distortionary SynthID-Text does not affect performance on these benchmarks. The unwatermarked `Gemma` metrics differ to those reported in Team et al. [28] due to different sampling configurations.

Watermarked text	LLM-paraphrased version
The actual cause of death in hypothermia is respiratory failure. When the body temperature drops below 95 degrees Fahrenheit, the respiratory center in the brain starts to fail, which leads to shallow breathing and slow heart rate. If the body temperature drops to below 90 degrees Fahrenheit, the heart and kidneys begin to fail, which eventually leads to death.	Hypothermia ultimately results in death due to the cessation of breathing. `\n\n`As the body's temperature falls below 95 degrees Fahrenheit, the brain's respiratory control center malfunctions, causing shallower breaths and a reduced heart rate. `\n\n`Further cooling to below 90 degrees Fahrenheit triggers the failure of the heart and kidneys, leading to the inevitable outcome of death."
A rock does not stay at 0 mph for a single moment during its motion. It has a velocity of 0 mph at the instant it is thrown up, but it gains acceleration due to gravity as it rises and reaches a maximum velocity at the highest point of its trajectory. After that, it decelerates and approaches 0 velocity as it falls back to the ground.	During its entire journey, a rock never maintains a velocity of 0 mph. When initially propelled upwards, it momentarily possesses a velocity of 0 mph; however, the force of gravity causes it to accelerate during its ascent, culminating in its maximum velocity at the peak of its path. Subsequently, the rock decelerates as it descends, its velocity approaching 0 mph upon its return to the ground.

Table C3: Examples of watermarked text after paraphrasing with `Gemini Ultra`.

C.7 Comparison to post-hoc methods

As discussed in Section 1, *post-hoc methods* are a family of AI text detection methods that use machine learning or other statistical signals [14–16]. However, these methods can have inconsistent performance, for example on out-of-domain data [16, 17]. In this section we demonstrate that (non-distortionary) SynthID-Text performs more

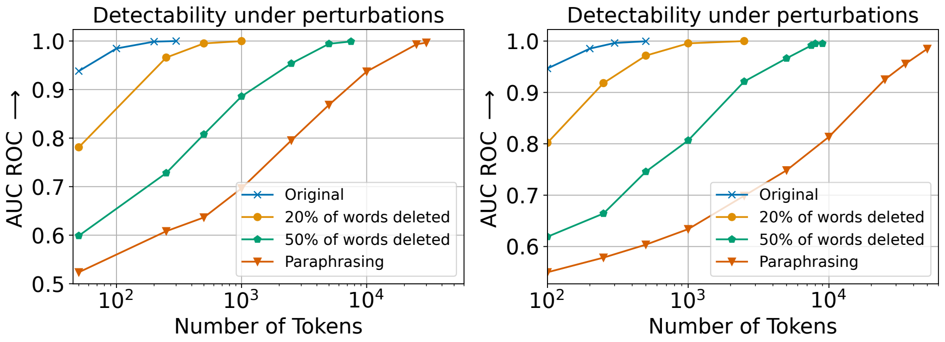

(a) `Gemma` 2B-IT (b) `Gemma` 7B-IT

Fig. C3: Detectability of SynthID-Text-watermarked text after applying perturbations to the watermarked text. Detectability is weakened by edits, particularly paraphrasing with a strong LLM (`Gemini Ultra`); however, the watermark is still detectable if the text is long enough.

consistently across different data sources than the most capable openly available post-hoc detector Binoculars [15]. Binoculars works by computing the cross-perplexity of the text with respect to two LLMs (the intuition being that text from two different LLMs is more similar than text from an LLM and text from a human). Hans et al. [15] report that Binoculars performs best using the `Falcon` 7B and `Falcon` 7B-instruct models [55]; we use these for our comparison.

To test detection performance across multiple languages, we evaluate both Binoculars and SynthID-Text across 8 languages, using the `XLSum` dataset [56]. To produce AI-generated text, for each language we use `Gemma` 7B-IT with SynthID-Text to generate 256 watermarked news articles from `XLSum` summaries, using one of the following two prompts: *'Read the following sentence carefully and then expand it to a news article:'* and *'Write a news article based on the following summary:'*. We performed no further filtering of generated text. We then evaluate detection performance, using an equal proportion of `XLSum` news articles as human-written data. Hans et al. [15] report that Binoculars performs more poorly on non-English and lower-resource languages, due to the fact that the `Falcon` models have limited capabilities in these languages. Indeed, in Figure C4 we see that Binoculars performs poorly on Hindi, Arabic and Russian; in contrast SynthID-Text detects all languages well.

Our results serve as a demonstration that like other generative watermarks, SynthID-Text is data-agnostic – its performance depending only on the length and entropy of the generated text; this is a significant advantage of generative watermarking compared to post-hoc methods. Other relative advantages of generative watermarking include the option to provide an interpretable decision (e.g. a p-value) that can be used to control the false positive rate; and not requiring the additional cost of running LLMs during detection. While our results indicate that generative

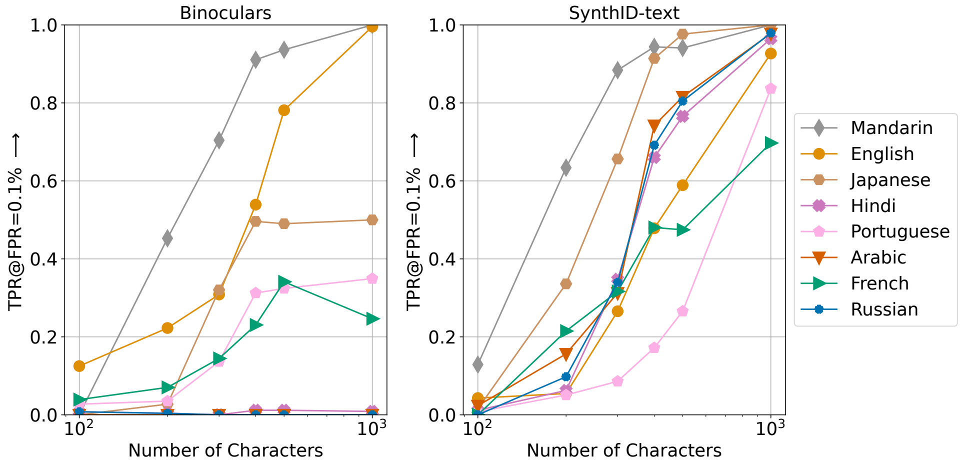

Fig. C4: Comparison of detection rates for `Gemma` 7B-IT-generated text in different languages: SynthID-Text watermarking vs. the post-hoc Binoculars detector [15]. We assess texts in 8 languages, prompted with `XLSum` [56]. Binoculars, which relies on cross-perplexity statistics drawn from underlying LLMs, performs poorly on some languages such as Hindi, Arabic and Russian. By contrast SynthID-Text performs well in all languages considered.

watermarking is a superior choice when one has control over the generation procedure, post-hoc methods remain a useful and complementary tool when that control is unavailable.

C.8 Selective Prediction

In some applications it may be critical to maintain a low false positive rate and a low false negative rate. In such scenarios, particularly if the texts are short or the LLM distribution has low entropy (e.g. due to low temperature or instruction tuning), the detection performance may be lower than desired. In this case we may use a selective prediction mechanism that abstains when it is uncertain about the presence or absence of the watermark in a piece of text. This allows us to achieve the desired error rates on the non-abstained texts.

The mechanism operates based on the principles of standard hypothesis testing [57]. For each length of text, we compute a threshold τ_{negative} on the watermarking scores that corresponds to the desired false negative rate (computed empirically based on a set of watermarked texts). Similarly, we compute a threshold τ_{positive} corresponding to a desired false positive rate, based on a set of unwatermarked texts. A given piece of text is classified as watermarked if its score is over the τ_{positive} threshold for its length, unwatermarked if its score is under τ_{negative}, and no prediction is made (abstention) if the score is between τ_{negative} and τ_{positive}. Note that when $\tau_{\text{positive}} < \tau_{\text{negative}}$, the scoring function's performance at that length already satisfies the desired error rates without need for abstention.

For example, suppose we require a false positive rate of 1% and a false negative rate of 5%. Extended Data Figure 3 shows the necessary abstention rates in order to achieve these error rates on the non-abstained texts, for `Gemma` 7B-IT at various temperatures and text lengths.

Appendix D Distortionary watermarking experiments

In this section we present our experiments comparing distortionary SynthID-Text to the Soft Red List watermark. We use the `Gemma` 7B-IT model, and a test set of 1500 prompts from the `ELI5` dataset. Extended Data Figure 2 shows the detectability/quality results for a variety of temperatures and text lengths.

Distortionary Tournament sampling settings

We evaluate Tournament sampling with the number of leaves per node (N) set to 2, 3, 4, 5, 7, 10, 15, 50 and 1000, and the number of layers (m) set to 2, 3, 4, 6, 8 and 10. For simplicity,we only plot the Pareto front of the tournament configurations in Extended Data Figure 2, showing the best detection performance given an allowance for quality (i.e. perplexity) degradation. To compute this, we consider various thresholds for perplexity (x-axis), and plot the best-performing tournament configuration with a perplexity less than this threshold.

Soft Red List settings

Following the methodology of Kirchenbauer et al. [23], we sweep over $\delta = 1, 2, 5, 10$ where δ is the scaling factor of the perturbation added to the logits, and $\gamma = 0.1, 0.25, 0.5, 0.75, 0.9$ where γ is the size of the green list as fraction of the LLM vocabulary. We also evaluate stronger watermarking with $\delta = 15, 20$. Similarly to Tournament sampling, we plot the Pareto front in Extended Data Figure 2.

Appendix E Vectorized Tournament sampling

In this section we derive vectorized formulations of Tournament sampling, providing an alternative but equivalent implementation to Methods Algorithm 2. First we define some notation:

Definition 9 (Watermarked distribution)**.** *Given a probability distribution p over V, a random seed $r \in \mathcal{R}$, a number of samples $N \geq 2$, a g-value distribution f_g, and a number of layers $m \geq 1$, the* watermarked distribution *$p_{wm}(\cdot|p, r, f_g, N, m)$ is the probability distribution of the winner of Methods Algorithm 2:*

$$p_{wm}(x_t|p, r, f_g, N, m) = \mathbb{P}\left[Alg2(p, r, f_g, N, m) \text{ returns } x_t\right].$$

Definition 10 . *Given a probability distribution p over V, random seed $r \in \mathcal{R}$, and g-values $\{g_\ell(x,r)\}_{x \in V}$ as defined in Methods Definition 4, we define the notation:*

$$p(V^{=g_\ell(x_t,r)}) := \sum_{x \in V : g_\ell(x,r) = g_\ell(x_t,r)} p(x)$$
$$p(V^{<g_\ell(x_t,r)}) := \sum_{x \in V : g_\ell(x,r) < g_\ell(x_t,r)} p(x)$$
$$p(V^{\leq g_\ell(x_t,r)}) := \sum_{x \in V : g_\ell(x,r) \leq g_\ell(x_t,r)} p(x).$$

E.1 Single-layer Tournament sampling

Theorem 11 (Vectorized form, single-layer Tournament sampling)**.** *Given a probability distribution p over V, random seed $r \in \mathcal{R}$, g-value distribution f_g, and number of samples $N \geq 2$, the watermarked distribution $p_{wm}(\cdot|p,r,f_g,N,m)$ for $m = 1$ is given by:*

$$p_{wm}(x_t|p,r,f_g,N,1) = \begin{cases} p(x_t)\left(\dfrac{p(V^{\leq g_1(x_t,r)})^N - p(V^{<g_1(x_t,r)})^N}{p(V^{=g_1(x_t,r)})}\right) & \text{if } p(x_t) \neq 0 \\ 0 & \text{if } p(x_t) = 0. \end{cases} \tag{E15}$$

Proof. See Supplementary Appendix K.2. □

E.1.1 Simplified formulations for special cases

In practice, Equation (E15) has simpler formulations for certain choices of the number of samples N or the g-value distribution f_g. All of our experiments use one the forms provided in this subsection.

Corollary 12 (Vectorized form, single-layer Tournament sampling, two samples)**.** *If in Theorem 11 the number of samples N equals 2, then:*

$$p_{wm}(x_t|p,r,f_g,N,1) = p(x_t)\left[p(V^{=g_1(x_t,r)}) + 2p(V^{<g_1(x_t,r)})\right]. \tag{E16}$$

Corollary 13 (Vectorized form, single-layer Tournament sampling, continuous g-values)**.** *If in Theorem 11 the g-value distribution f_g is continuous (i.e. the probability that two g-values are the same is zero) then:*

$$p_{wm}(x_t|p,r,f_g,N,1) = \left(p(x_t) + p(V^{<g_1(x_t,r)})\right)^N - p(V^{<g_1(x_t,r)})^N. \tag{E17}$$

In particular if $N = 2$, then:

$$p_{wm}(x_t|p,r,f_g,2,1) = p(x_t)\left[p(x_t) + 2p(V^{<g_1(x_t)})\right]. \tag{E18}$$

Corollary 14 (Vectorized form, single-layer Tournament sampling, binary g-values)**.** *If in Theorem 11 the g-value distribution f_g is binary (i.e. all g-values are 0 or 1) then:*

$$p_{wm}(x_t|p,r,f_g,N,1) = \begin{cases} p(x_t)p(V^{g_1=0})^{N-1} & \text{if } g_1(x_t,r)=0 \\ p(x_t)\left(\dfrac{1-p(V^{g_1=0})^N}{p(V^{g_1=1})^{N-1}}\right) & \text{if } g_1(x_t,r)=1 \end{cases} \tag{E19}$$

where the notation $p(V^{g_1=0})$ means $\sum_{x\in V:g_1(x,r)=0} p(x)$ and similarly for $p(V^{g_1=1})$. In particular, if $N=2$, then:

$$p_{wm}(x_t|p,r,f_g,2,1) = p(x_t)\left[1+g_1(x_t,r)-p(V^{g_1=1})\right]. \tag{E20}$$

E.2 Multi-layer Tournament sampling

Now we show that we can simply repeatedly apply Equation (E15) (or one of the special cases in Supplementary Appendix E.1.1) to obtain the vectorized form of a multi-layer tournament:

Theorem 15 (Vectorized form, multi-layer Tournament sampling)**.** *Given a probability distribution $p \in \triangle V$, a number of samples $N \geq 2$, and a set of real values $\{g(x)\}_{x\in V}$, define the transformation W which gives a distribution $W(p,g(\cdot),N) \in \triangle V$:*

$$W(p,g(\cdot),N)(x_t) = \begin{cases} p(x_t)\left(\dfrac{p(V^{\leq g(x_t)})^N - p(V^{<g(x_t)})^N}{p(V^{=g(x_t)})}\right) & \text{if } p(x_t)\neq 0 \\ 0 & \text{if } p(x_t)=0. \end{cases} \tag{E21}$$

Now, given a random seed $r \in \mathcal{R}$, g-value distribution f_g, number of samples $N \geq 2$, and number of layers $m \geq 1$, consider the following sequence of distributions, defined through repeated application of W:

$$\begin{aligned} p^{(1)}_{wm}(\cdot) &:= W(p, g_1(\cdot,r), N) \\ p^{(2)}_{wm}(\cdot) &:= W(p^{(1)}_{wm}, g_2(\cdot,r), N) \\ &\cdots \\ p^{(m)}_{wm}(\cdot) &:= W(p^{(m-1)}_{wm}, g_m(\cdot,r), N). \end{aligned} \tag{E22}$$

It follows that $p^{(m)}_{wm}(\cdot)$ is equal to the m-layer Tournament watermarked distribution $p_{wm}(\cdot|p,r,f_g,N,m)$ (Definition 9).

Proof. Proof by induction on m. The base case $m=1$ is given by Theorem 11.

For the induction case, suppose Theorem 15 is true for $m-1$. Now consider an m-layer tournament; it is equivalent to running N-many $(m-1)$-layer tournaments and then putting the winners into a single-layer tournament using $g_m(\cdot,r)$. By the induction assumption, the N winners are drawn from $p^{(m-1)}_{\text{wm}}(\cdot)$ as defined in

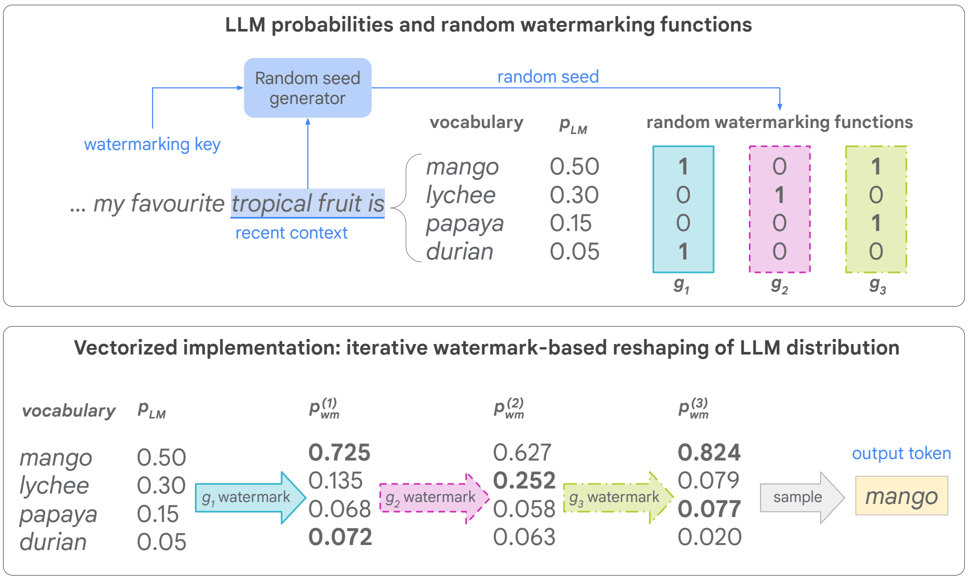

Fig. E5: Illustration of the vectorized implementation of SynthID-Text watermarking for the same example as Figure 2 in the main paper. Each 'watermark' arrow corresponds to a tournament layer, and represents an application of Equation (E20), which modifies the LLM distribution based on a random watermarking function g_ℓ. The output token is sampled from the final distribution after all layers (here, 3) have been applied.

Equation (E22), and by Theorem 11 the winner of the single-layer tournament is given by $W(p_{\text{wm}}^{(m-1)}, g_m(\cdot, r), N)$. □

E.3 Implementation

Theorem 15 provides an alternative implementation to Algorithm 2 for a multi-layer tournament: instead of sampling and running a tournament, we can simply compute Equations E22 to obtain the watermarked distribution $p_{\text{wm}}(\cdot|p, r, f_g, N, m)$, then sample directly from it. Figure E5 shows how this works for the three-layer ($m = 3$) two-sample ($N = 2$) tournament with binary g-values previously presented in Figure 2 in the main paper.

One advantage of the vectorized implementation is that it provides the entire watermarked distribution (which can be useful for downstream purposes), whereas the tournament implementation provides just one sample from the watermarked distribution. The two implementations have different computational advantages; see Supplementary Appendix F. In practice we use the vectorized formulation for our experiments.

Method	Samples	g-value computations	Other operations
Tournament (Alg 2)	N^m	$\min(m\|V\|, N^{m+1})$	$N^m - 1$
Vectorised tournament, general (Thm 15)	1	$m\|V\|$	$O(m\|V\| \log \|V\|)$
Vectorised tournament, binary g-values (Cor 14)	1	$m\|V\|$	$O(m\|V\|)$
Gumbel sampling	0	$\|V\|$	$O(\|V\|)$
Soft Red List	1	$\|V\|$	$O(\|V\|)$

Table F4: Computational complexity of the Tournament, Gumbel, and Soft Red List sampling algorithms. $|V|$ is the size of the support of the LLM distribution as defined in Methods Definition 1. For Tournament sampling, m is number of layers and N is the number of samples per node. Proofs are given in Supplementary Appendix K.3.

Appendix F Computational complexity

In Table F4 we summarise the theoretical computational complexity of the Tournament, Gumbel, and Soft Red List sampling algorithms. Tournament sampling generally has higher computational complexity than Gumbel or Soft Red List sampling; however if $|V|$ is large compared to N^{m+1} then Tournament sampling (the tournament-based Methods Algorithm 2 implementation) may have lower complexity. Nonetheless, in the context of the computational complexity of generating text from a large LLM, these differences are in practice negligible (see Section 3 in main paper).

When implementing Tournament sampling, there is the option to use the vectorised version presented in Supplementary Appendix E, instead of the tournament-style implementation presented in Methods Algorithm 2. Furthermore, the complexity of the vectorised version depends on our choice of g-value distribution; if we are using binary g-values (e.g. Bernoulli g-value distribution) the complexity is lower than if we are using continuous g-values (e.g. Uniform g-value distribution). In our experiments, we find that the vectorised implementation is faster than the tournament-style implementation – in general this is true especially if N^m is large compared to $|V|$. However, if $|V|$ is comparatively large, then the tournament-style implementation may be faster. Note that $|V|$ is the size of the support of the LLM distribution $p_{\text{LM}}(\cdot|x_{<t})$ as defined in Methods Definition 1; if top-p or top-k truncation is applied, this can be considerably smaller than the size of the LLM's full vocabulary.

Appendix G Non-distortion

Ideally, a watermark should not distort the LLM's output distribution, as we would like watermarked text to have the same quality as text from the unwatermarked LLM. In this section we show that Tournament sampling with $N = 2$ samples is *non-distortionary* at the token level, and when paired with repeated context masking, is non-distortionary at the (multi-)sequence level too. We then discuss these different levels of non-distortion and their trade-offs.

G.1 Non-distortion at the token level

A sampling algorithm (Methods Definition 5) is *non-distortionary* as defined by Kuditipudi et al. [24][2] if in expectation over the random seed r, the watermarked distribution is equal to the original LLM distribution. We call this property *single-token non-distortion*:

Definition 16 (Single-token non-distortionary sampling algorithm)**.** *A sampling algorithm $\mathcal{S} : \triangle V \times \mathcal{R} \to V$ is (single-token) non-distortionary if for any probability distribution $p \in \triangle V$ and token $x \in V$:*

$$\mathbb{E}_{r\sim Unif(\mathcal{R})}\left[\mathbb{P}\left(\mathcal{S}(p,r)=x\right)\right] = p(x).$$

If $\mathcal{S}$ is not non-distortionary, we call it distortionary.

Definition 16 is an important property of a sampling algorithm, providing a guarantee at the single token level; specifically, that $\mathcal{S}$ is a valid pseudorandom sampler with respect to the seed r. However, it makes no guarantee at the sequence level; for this reason we refer to Definition 16 as *single-token non-distortion*, to differentiate it from sequence-level non-distortion (discussed in the next subsection). Of our baseline sampling algorithms, Gumbel sampling (Supplementary Appendix B.1.1) is non-distortionary and Soft Red List (Supplementary Appendix B.1.2) is distortionary.

We now show in the next three theorems that two-sample ($N = 2$) Tournament sampling is a non-distortionary sampling algorithm (single-layer and multi-layer); however, Tournament sampling with $N > 2$ samples is distortionary. These theorems refer to the watermarked distribution p_{wm} from Definition 9.

Theorem 17 (Single-layer two-sample Tournament sampling is non-distortionary)**.** *For any probability distribution p over V, g-value distribution f_g, and token $x_t \in V$:*

$$\mathbb{E}_{r_t\sim Unif(\mathcal{R})}\left[p_{wm}(x_t|p, r_t, f_g, 2, 1)\right] = p(x_t).$$

Proof. See Supplementary Appendix K.4. □

Theorem 18 (Multi-layer two-sample Tournament sampling is non-distortionary)**.** *For any probability distribution p over V, g-value distribution f_g, number of layers $m \geq 1$, and token $x_t \in V$:*

$$\mathbb{E}_{r_t\sim Unif(\mathcal{R})}\left[p_{wm}(x_t|p, r_t, f_g, 2, m)\right] = p(x_t). \tag{G23}$$

Proof. See Supplementary Appendix K.5. □

Theorem 19 (Tournament sampling is distortionary for $N > 2$ samples)**.** *Given any g-value distribution f_g (that is not one-hot) and any integer $N > 2$, then single-layer Tournament sampling using f_g and N is distortionary.*

[2] Kuditipudi et al. [24] call this property *distortion-free*.

Proof. See Supplementary Appendix K.6. □

G.2 Non-distortion at the (multi-)sequence level

We now move to a notion of non-distortion at the level of one or more sequences. We define a watermarking scheme to be K*-sequence non-distortionary* if the probability of the watermarked model generating a particular sequence of $K \geq 1$ responses to a particular sequence of K prompts supplied consecutively is, in expectation over the watermarking key, the same as generating them from the original model. Our definition is similar to the K*-shot undetectable* property defined by Hu et al. [27], though we generalize it to the case where the K prompts may be different.

To give the formal definition, we first define some notation. Given a sequence of K prompts $\mathbf{x}^1, \ldots, \mathbf{x}^K \in V^*$ (where V^* is the set of all finite sequences in V) and given a sequence of K responses $\mathbf{y}^1, \ldots, \mathbf{y}^K \in V^*$, we write $\mathbb{P}_{\text{wm}}\left(\mathbf{y}^i|\mathbf{x}^i, k; (\mathbf{x}^1, \mathbf{y}^1), \ldots, (\mathbf{x}^{i-1}, \mathbf{y}^{i-1})\right)$ to denote the probability of the watermarking scheme using watermarking key k generating response $\mathbf{y}^i$ in response to prompt $\mathbf{x}^i$, given that the last $i-1$ prompt/response pairs to be supplied to/generated by the watermarked model are $(\mathbf{x}^1, \mathbf{y}^1), \ldots, (\mathbf{x}^{i-1}, \mathbf{y}^{i-1})$. Then:

Definition 20 (K-sequence non-distortionary watermarking scheme)**.** *A watermarking scheme $\mathbb{P}_{wm}$ is K-sequence non-distortionary for some $K \geq 1$ if, for any sequence of K prompts $\mathbf{x}^1, \ldots, \mathbf{x}^K \in V^*$ and sequence of K responses $\mathbf{y}^1, \ldots, \mathbf{y}^K \in V^*$:*

$$\mathbb{E}_{k \sim Unif(\mathcal{R})}\left[\prod_{i=1}^{K} \mathbb{P}_{wm}\left(\mathbf{y}^i|\mathbf{x}^i, k; (\mathbf{x}^1, \mathbf{y}^1), \ldots, (\mathbf{x}^{i-1}, \mathbf{y}^{i-1})\right)\right] = \prod_{i=1}^{K} p_{LM}(\mathbf{y}^i|\mathbf{x}^i).$$

This definition extends the notion of non-distortion from a single token (Definition 16) to one or more consecutively-generated sequences. In particular, while Definition 16 is a property of the sampling algorithm alone (such as Gumbel or Tournament sampling), Definition 20 is a property of the whole watermarking scheme (which includes the sampling algorithm, the random seed generator, and any other details of how the watermarked LLM is operated across multiple queries).

We now show that by applying K*-sequence repeated context masking* (Methods Section 5.6) with a non-distortionary sampling algorithm, we can construct a K-sequence non-distortionary watermarking scheme:

Theorem 21 (K-sequence repeated context masking + non-distortionary sampling algorithm → K-sequence non-distortionary watermarking scheme)**.** *Let $\mathcal{S}$ be a non-distortionary sampling algorithm (Def 16). For any $K \geq 1$, let $\mathbb{P}_{wm}$ denote the watermarking scheme that applies $\mathcal{S}$ with sliding window random seed generation and K-sequence repeated context masking (Methods Algorithm 3). Then $\mathbb{P}_{wm}$ is K-sequence non-distortionary.*

Proof. See Supplementary Appendix K.7. □

In particular, Theorem 21 with Theorem 18 tells us that two-sample ($N = 2$) Tournament sampling is K-sequence non-distortionary if applied with K-sequence repeated

context masking. The same is true for other non-distortionary sampling algorithms such as Gumbel sampling (Supplementary Appendix B.1.1).

G.3 Discussion

In this section we have defined several levels of non-distortion that a watermarking scheme may satisfy; from weakest to strongest they are:

- Single-token non-distortion (Definition 16)
- Single-sequence non-distortion (Definition 20 for $K = 1$)
- K-sequence non-distortion (Definition 20 for a particular integer $K > 1$)
- Infinite-sequence non-distortion (Definition 20 for $K = \infty$)

Single-token non-distortion can be achieved by using any non-distortionary sampling algorithm such as Gumbel sampling or Tournament sampling with $N = 2$; however, in circumstances where high detectability is more important than quality preservation, one might choose to use a distortionary sampling algorithm such as Soft Red List or Tournament sampling with $N > 2$.

Single-sequence non-distortion is an important property as it guarantees that the quality of a watermarked response is on average the same as an unwatermarked response. In particular, a single-sequence non-distortionary watermarking scheme will not cause repeating loops or lower diversity *within* a response – a phenomenon which has been observed in schemes that lack single-sequence non-distortion (e.g., using a sliding window random seed generator without repeated context masking) [24, 25]. A single-sequence non-distortionary watermarking scheme should match the unwatermarked model on any evaluation comprising measurements on individual responses, such as perplexity (Table C1), pairwise quality assessment (Extended Data Table 1), and other automatic benchmarks (Table C2). In our experiments with Gumbel and $N = 2$ Tournament sampling we use 1-sequence repeated context masking and so achieve single-sequence non-distortion.

While single-sequence non-distortion guarantees the quality of each individual response, it does not necessarily preserve diversity across multiple responses. This can be observed in Extended Data Figure 4, which shows that when sampling several responses to the same prompt, the similarity between the responses is greater for the watermarked responses than the unwatermarked responses. This could be problematic in scenarios where inter-response diversity is important, or could lower the overall quality of a system which generates many responses then selects the best one. It could also be problematic from a security perspective, as an adversary might steal the watermark by detecting the repeated biases that appear across multiple responses [32].

If these concerns are particularly important, one can choose a watermarking scheme achieving K-sequence non-distortion for a larger $K > 1$; however there are some trade-offs. The primary trade-off is detectability: if we apply K-sequence repeated context masking with larger K then the watermark will be masked more often, reducing its detectability. Another trade-off is the computational complexity and storage requirements of maintaining the context history, particularly for large K. Ultimately, complete theoretical non-distortion (infinite-sequence non-distortion) can be achieved

by implementing infinite repeated context masking, but this is impractical from a computational and detectability point of view.

Appendix H Analysis of watermarking strength

The intuition of Tournament sampling is that it returns a token that is likely to have larger g-values; these high g-values are what is later measured when detecting the watermark. The watermarking strength is related to how much higher these g-values are for watermarked text compared to unwatermarked text. In this section we quantify this bias, and first show that it is greater when we use more samples N in the tournament. Second, we show the bias is greater when the LLM has high entropy (in particular, collision entropy), but that each layer of watermarking reduces the entropy of the distribution.

H.1 Notation

Definition 22 (Collision probability)**.** *Given a probability distribution p, the* collision probability *C_p of p is the probability that two samples drawn i.i.d. from p are the same. If $p = (p_i)_{i=1}^N$ is discrete, the collision probability equals $\sum_{i=1}^N p_i^2$.*

Collision probability is related to *collision entropy*, sometimes called *Rényi entropy*, $H_2(p) = -\log \sum_{i=1}^N p_i^2$.

Definition 23 (Higher-order collision probabilities)**.** *Given a probability distribution p and integers $N, j \geq 1$, let $C_p^{N,j}$ denote the probability that N samples drawn i.i.d. from p have exactly j unique values. Note that $C_p^{2,1}$ is the collision probability of p. In general, we refer to $C_p^{N,j}$ as the* higher-order collision probabilities *of p.*

Definition 24 (Watermarked g-value distribution)**.** *Given a probability distribution p, a g-value distribution f_g, and number of samples $N \geq 2$, let F_{gw} denote the cumulative density function of the g-value of a token sampled from the single-layer watermarked distribution $p_{wm}(\cdot|p, r, f_g, N, 1)$ (Definition 9), in expectation over the random seed r:*

$$F_{gw}(z) := \mathbb{P}_{r \sim Unif(\mathcal{R}), x \sim p_{wm}(\cdot|p,r,f_g,N,1)} \left[g_1(x, r) \leq z \right].$$

Let f_{gw} denote the probability density/mass function corresponding to F_{gw}. We refer to f_{gw} as the watermarked g-value distribution*.*

The watermarking strength of a single layer of Tournament sampling can therefore be described as the distributional difference between the watermarked g-value distribution f_{gw} (which describes the expected g-value distribution of the watermarked token) and the 'unwatermarked' g-value distribution f_g (which describes the expected g-value distribution of the unwatermarked token).

H.2 Watermarked g-value distribution

The following theorem describes the watermarked g-value distribution f_{gw} in terms of the unwatermarked g-value distribution f_g and the higher-order LLM collision probabilities $C_{p_{\mathrm{LM}}}^{N,j}$.

Theorem 25 (Watermarked g-value distribution for single-layer tournament)**.** *Given a probability distribution p_{LM}, a g-value distribution f_g, and number of samples $N \geq 2$, the c.d.f. of the watermarked g-value distribution F_{gw} is given by:*

$$F_{gw}(z) = \sum_{j=1}^{N} C_{p_{LM}}^{N,j} F_g(z)^j. \tag{H24}$$

If f_g is continuous, the p.d.f. of the watermarked g-value distribution f_{gw} is given by:

$$f_{gw}(z) = f_g(z) \sum_{j=1}^{N} C_{p_{LM}}^{N,j} j F_g(z)^{j-1}. \tag{H25}$$

If f_g is discrete, the p.m.f. of the watermarked g-value distribution f_{gw} is given by:

$$f_{gw}(z) = f_g(z) \sum_{j=1}^{N} C_{p_{LM}}^{N,j} \left(\sum_{k=1}^{j} (-1)^{k-1} \binom{j}{k} F_g(z)^{j-k} f_g(z)^{k-1} \right). \tag{H26}$$

Proof. See Supplementary Appendix K.8. □

Theorem 25 shows that the watermarked g-value distribution depends on how much collision entropy there is in the LLM distribution. In particular, Equation (H24) says that the watermarked c.d.f. F_{gw} is a linear combination of powers of the unwatermarked c.d.f. F_g, with $C_{p_{\mathrm{LM}}}^{N,j}$ as the coefficients. If p_{LM} is high-entropy, then $\{C_{p_{\mathrm{LM}}}^{N,j}\}^{j=1,\ldots,N}$ is more heavily weighted towards the larger values of j, and so F_{gw} is more weighted towards the higher powers of F_g; this biases the distribution of the watermarked g-value to be larger.

H.2.1 Simplified formulations for special cases

For certain special cases (e.g., choices of N or f_g), Theorem 25 has simplified forms, which we provide here.

Corollary 26 (Watermarked g-value distribution for single-layer tournament, two samples)**.** *If in Theorem 25 the number of samples N is equal to 2, then the c.d.f. F_{gw} is given by:*

$$F_{gw}(z) = C_{p_{LM}} F_g(z) + (1 - C_{p_{LM}}) F_g(z)^2. \tag{H27}$$

If g is continuous, the p.d.f. f_{gw} is given by:

$$f_{gw}(z) = f_g(z)\left[C_{p_{LM}} + 2(1 - C_{p_{LM}})F_g(z)\right]. \tag{H28}$$

If g is discrete, the p.m.f. f_{gw} is given by:

$$f_{gw}(z) = f_g(z)\left[C_{p_{LM}} + (1 - C_{p_{LM}})\left(2F_g(z) - f_g(z)\right)\right]. \tag{H29}$$

Proof. Follows from Theorem 25 and $C^{2,1}_{p_{\text{LM}}} = C_{p_{\text{LM}}}$ and $C^{2,2}_{p_{\text{LM}}} = 1 - C_{p_{\text{LM}}}$. □

Corollary 27 (Watermarked g-value distribution for single-layer tournament, two samples, Bernoulli g-value distribution)**.** *If in Theorem 25 the number of samples N is equal to 2 and the g-value distribution f_g is Bernoulli(q) for some $0 < q < 1$, then the watermarked g-value distribution is given by the p.m.f.:*

$$f_{gw}(1) = q + q(1 - q)(1 - C_{p_{LM}}). \tag{H30}$$

In particular, if $q = 0.5$ then:

$$f_{gw}(1) = \frac{1}{2} + \frac{1}{4}(1 - C_{p_{LM}}).$$

Proof. This follows from Equation (H29) in Corollary 26. □

Equation (H30) shows that for a Bernoulli g-value distribution, the expected watermarked g-value $f_{gw}(1)$ is greater than the expected unwatermarked g-value (which is q); furthermore, it increases linearly with the LLM's non-collision probability $(1 - C_{p_{\text{LM}}})$.

Corollary 28 (Watermarked g-value distribution for single-layer tournament, two samples, Uniform g-value distribution)**.** *If in Theorem 25 the number of samples N is equal to 2 and the g-value distribution f_g is Uniform[0,1], then the watermarked g-value distribution is given by the p.d.f.:*

$$f_{gw}(z) = C_{p_{LM}} + 2(1 - C_{p_{LM}})z \qquad \forall\ 0 \le z \le 1.$$

Furthermore the expected watermarked g-value is:

$$\mathbb{E}_{r\sim \mathit{Unif}(\mathcal{R}),x\sim p_{wm}(\cdot|p,r,f_g,2,1)}\left[g_1(x,r)\right] = \frac{1}{2} + \frac{1}{6}(1 - C_{p_{LM}}). \tag{H31}$$

Proof. The p.d.f. follows from Equation (H28) in Corollary 26. The expected value follows from integrating:

$$\int_0^1 z f_{gw}(z)dz = \int_0^1 C_{p_{\text{LM}}} z + 2(1 - C_{p_{\text{LM}}})z^2 dz$$

$$= \frac{C_{p_{\text{LM}}}}{2} + \frac{2(1 - C_{p_{\text{LM}}})}{3}$$
$$= \frac{1}{2} + \frac{1}{6}(1 - C_{p_{\text{LM}}}).$$

□

Equation (H31) shows that for a Uniform g-value distribution, the expected watermarked g-value is greater than the expected unwatermarked g-value (which is $\frac{1}{2}$); and it increases linearly with the LLM's non-collision probability $(1 - C_{p_{\text{LM}}})$.

H.3 Stronger watermarking with larger N

Theorem 25 shows that watermarking strength depends on the number of samples N used in the tournament. In this section we provide two results about how watermarking strength changes as N increases: First, Theorem 29 shows that, provided there is some entropy in the LLM distribution, a single layer of Tournament sampling using $N + 1$ samples provides greater watermarking strength than one using N samples. Then, Corollary 30 shows that, provided the LLM distribution has sufficiently large support, we can achieve arbitrarily high watermarking strength by increasing the number of samples N.

Theorem 29 (g-value bias increases with N, single-layer tournament)**.** *Given a probability distribution p_{LM} and g-value distribution f_g, let F^N_{gw} be the c.d.f. of the watermarked g-value distribution for a single-layer tournament with N samples. Let F^{N+1}_{gw} be the same for a single-layer tournament with $N + 1$ samples. Then for all z:*

$$F^{N+1}_{gw}(z) \leq F^N_{gw}(z).$$

When $0 < F^N_{gw}(z) < 1$, equality holds iff p_{LM} is one-hot.

Proof. See Supplementary Appendix K.9. □

Corollary 30 (Watermarked g-value distribution for single-layer tournament as $N \to \infty$)**.** *Given a probability distribution p_{LM} and g-value distribution f_g: for all z, the c.d.f. of the watermarked g-value distribution $F_{gw}(z) \to F_g(z)^V$ as $N \to \infty$, where V is the size of the support of p_{LM}.*

Proof. Equation (H24) gives us:

$$F_{gw}(z) = \sum_{j=1}^{N} C^{N,j}_{p_{\text{LM}}} F_g(z)^j.$$

For $N > V$, $C^{N,j}_{p_{\text{LM}}} = 0$ for all $j > V$. Furthermore as $N \to \infty$, $C^{N,V}_{p_{\text{LM}}} \to 1$ and $C^{N,j}_{p_{\text{LM}}} \to 0$ for all $j \leq V - 1$. It follows that $F_{gw}(z) \to F_g(z)^V$. □

H.4 Entropy analysis for $N = 2$

Corollary 26 shows that for $N = 2$ samples, the watermarking strength of a single layer of Tournament sampling depends on the collision probability of the input distribution. For a multi-layer tournament, this means that the watermarking strength of each layer depends on the collision probability of the previous layer. In this section we show that the expected collision probability increases (and so the expected watermarking strength of each layer decreases) with each added layer.

First, in Theorem 31 we derive the expected collision probability of the single-layer watermarked distribution; then in Theorem 32 we show this is greater than the collision probability of the input distribution.

Theorem 31 (Expected collision probability for single-layer tournament, two samples)**.** *Given a probability distribution p_{LM}, random seed $r \in \mathcal{R}$ and g-value distribution f_g, let $C^{2,1}_{p_{wm}}$ denote the collision probability of the watermarked distribution $p_{wm}(\cdot|p_{LM}, r, f_g, 2, 1)$ for a $N = 2$ sample single-layer tournament. In expectation over the random seed r, the collision probability is:*

$$\mathbb{E}_{r\sim Unif(\mathcal{R})}\left[C^{2,1}_{p_{wm}}\right] = \left[\frac{4}{3} - \frac{1}{3}C^{3,1}_{f_g}\right] C^{2,1}_{p_{LM}} + \left[\frac{2}{3} + \frac{1}{3}C^{3,1}_{f_g} - C^{2,1}_{f_g}\right]\left(C^{2,1}_{p_{LM}}\right)^2 - \left[\frac{2}{3} - \frac{2}{3}C^{3,1}_{f_g}\right] C^{3,1}_{p_{LM}} - \left[\frac{1}{3} + \frac{2}{3}C^{3,1}_{f_g} - C^{2,1}_{f_g}\right] C^{4,1}_{p_{LM}}. \tag{H32}$$

where $C^{N,j}_{p_{LM}}$ and $C^{N,j}_{g}$ are the higher order collision probabilities (Def 23), respectively, of p_{LM} and f_g.

Proof. See Supplementary Appendix K.10. □

Theorem 32 (Single-layer tournament increases the expected collision probability, two samples)**.** *The expected collision probability of a single-layer tournament with $N = 2$ samples is greater than or equal to the LLM collision probability: $\mathbb{E}_{r\sim Unif(\mathcal{R})}\left[C^{2,1}_{p_{wm}}\right] \geq C^{2,1}_{p_{LM}}$, with equality iff p_{LM} is one-hot.*

Proof. See Supplementary Appendix K.11. □

In the case of a multi-layer tournament, Theorem 32 says that the sequence of m watermarked distributions (see Definition 9):

$$p_{\text{wm}}(\cdot|p_{\text{LM}}, r, f_g, 2, 1),\ \ p_{\text{wm}}(\cdot|p_{\text{LM}}, r, f_g, 2, 2),\ \ \ldots,\ \ p_{\text{wm}}(\cdot|p_{\text{LM}}, r, f_g, 2, m)$$

have (in expectation over r) increasing collision probability (i.e., decreasing collision entropy). Thus the amount of watermarking strength contributed by each new layer decreases. For the tournament as a whole, this implies that increasing the number of layers m may give diminishing returns in terms of overall watermarking strength.

H.4.1 Effect of g-value distribution f_g

Now turning to the particular choice of f_g, the following result shows that a Uniform[0,1] layer raises the collision probability of the next layer (and so reduces its watermarking strength) more than a Bernoulli(0.5) layer does. This suggests a natural trade-off: while a single Uniform layer provides more watermarking strength than a single Bernoulli layer, it also more greatly reduces the amount of entropy available to be used by subsequent layers.

Corollary 33 (Expected collision probability for single-layer tournament, two samples, Bernoulli(0.5) or Uniform(0,1) g-value distribution)**.** *If $f_g = Bernoulli(0.5)$ then Equation (H32) equals:*

$$\mathbb{E}_{r\sim Unif(\mathcal{R})}\left[C^{2,1}_{p_{wm}}\right] = \frac{5}{4}C^{2,1}_{p_{LM}} + \frac{1}{4}\left(C^{2,1}_{p_{LM}}\right)^2 - \frac{1}{2}C^{3,1}_{p_{LM}}. \tag{H33}$$

If $f_g = Uniform[0,1]$ then Equation (H32) equals:

$$\mathbb{E}_{r\sim Unif(\mathcal{R})}\left[C^{2,1}_{p_{wm}}\right] = \frac{4}{3}C^{2,1}_{p_{LM}} + \frac{2}{3}\left(C^{2,1}_{p_{LM}}\right)^2 - \frac{2}{3}C^{3,1}_{p_{LM}} - \frac{1}{3}C^{4,1}_{p_{LM}}. \tag{H34}$$

Furthermore, for any distribution p_{LM}, $\mathbb{E}_{r\sim Unif(\mathcal{R})}\left[C^{2,1}_{p_{wm}}\right]$ is greater for $f_g = Uniform[0,1]$ than for $f_g = Bernoulli(0.5)$.

Proof. For Equation (H33), substitute $C^{2,1}_{f_g} = \frac{1}{2}$ and $C^{3,1}_{f_g} = \frac{1}{4}$ into Equation (H32). For Equation (H34), substitute $C^{2,1}_{f_g} = C^{3,1}_{f_g} = 0$. Now the difference:

$$\begin{aligned}
&\mathbb{E}_{r\sim\text{Unif}(\mathcal{R})}\left[C^{2,1}_{p_\text{wm},\text{Unif}}\right] - \mathbb{E}_{r\sim\text{Unif}(\mathcal{R})}\left[C^{2,1}_{p_\text{wm},\text{Ber}}\right]\\
=&\frac{1}{12}C^{2,1}_{p_\text{LM}} + \frac{5}{12}\left(C^{2,1}_{p_\text{LM}}\right)^2 - \frac{1}{6}C^{3,1}_{p_\text{LM}} - \frac{1}{3}C^{4,1}_{p_\text{LM}}\\
\geq&\frac{1}{12}C^{2,1}_{p_\text{LM}} + \frac{5}{12}\left(C^{2,1}_{p_\text{LM}}\right)^2 - \frac{1}{6}\left(\frac{1}{2}C^{2,1}_{p_\text{LM}}(1+C^{2,1}_{p_\text{LM}})\right) - \frac{1}{3}\left(C^{2,1}_{p_\text{LM}}\right)^2 && \text{(Lemma 44)}\\
=&0. && \text{(simplify)}
\end{aligned}$$

□

H.5 Discussion

As shown in Supplementary Appendix H.4, the amount of watermarking evidence contributed by each layer decreases as more layers are added. Consequently, if we keep adding layers to a multi-layer tournament, at some point the noise outweighs the signal, and the detectability of the watermark begins to degrade. However, the optimal number of layers depends on the particular collision probabilities of the LLM distribution, which itself varies step-to-step and also depends on the prompt distribution. For our experiments, we determine the optimal number of layers empirically (Supplementary Appendix C.1).

The choice of the g-value distribution f_g used in Tournament sampling (Methods Definition 3) also plays a key role in the detectability of the watermark. In Supplementary Appendix H.4 we showed theoretically that while a single layer with f_g = Uniform[0, 1] provides more watermarking evidence than a single layer with f_g = Bernoulli(0.5), on the other hand the Uniform layer more greatly reduces the amount of entropy available in the output distribution, meaning that subsequent layers have lower watermarking strength. Intuitively, this means that with Uniform, Tournament sampling can apply a few layers of strong watermarking, and with Bernoulli, Tournament sampling can apply many layers of weak watermarking. This corresponds with our empirical observations that for shallow tournaments (small number of layers), Uniform generally outperforms Bernoulli in terms of overall watermark detectability, while for deeper tournaments, Bernoulli outperforms Uniform. If we are free to choose any number of layers, we find that overall the best watermark detectability is usually achieved with many layers of weak Bernoulli watermarking, rather than fewer layers of strong Uniform watermarking.

Appendix I Generative watermarking with speculative sampling

Speculative sampling [5] is an algorithm designed to speed up sampling text from a large target LLM q, by using a smaller draft LLM p. As speculative sampling is commonly used in production, we wish to combine speculative sampling with generative watermarking. In this section we introduce speculative sampling, then discuss the desired properties of a combined solution; finally we present two algorithms for combining a generative watermark (such as SynthID-Text) with speculative sampling.

I.1 Speculative sampling

The algorithm for speculative sampling is presented in Algorithm 4.[3]
Algorithm 4 uses the $(\cdot)_+$ operator on Line 13, which is defined as:

Definition 34 ($(\cdot)_+$ operator)**.**

$$(f(x))_+ := \frac{\max(0, f(x))}{\sum_{x'} \max(0, f(x'))}.$$

In Algorithm 4, the draft LLM's suggestions are either accepted or rejected by the target LLM. This is the *acceptance rate*:

[3]Algorithm 4 is the same as the algorithm in [5], though we fix some minor notational confusion in the original incrementing both n and t. It also overloads t as both the prompt length and the iterator from 1 to K; but we keep this to be consistent with the original.

Algorithm 4 Speculative sampling [5]

1: Given lookahead K, minimum target sequence length T, target model $q(\cdot|\cdot)$, draft model $p(\cdot|\cdot)$, initial prompt sequence $x_1, \ldots, x_t$.
2: Initialize $n \leftarrow t$.
3: **while** $n < T$ **do**
4:   **for** $t = 1 : K$ **do**
5:     Sample draft auto-regressively $\tilde{x}_t \sim p(\cdot|x_{1:n}, \tilde{x}_{1:t-1})$
6:   **end for**
7:   In parallel, compute $K+1$ sets of logits from drafts $\tilde{x}_1, \ldots, \tilde{x}_K$: $q(\cdot|x_{1:n}),\ q(\cdot|x_{1:n}, \tilde{x}_1),\ \ldots,\ q(\cdot|x_{1:n}, \tilde{x}_{1:K})$
8:   **for** $t = 1 : K$ **do**
9:     Sample $r \sim U[0,1]$ from a uniform distribution.
10:     **if** $r < \min\left(1, q(\tilde{x}_t|x_{1:n})/p\left(\tilde{x}_t|x_{1:n}\right)\right)$ **then**
11:       Set $x_{n+1} \leftarrow \tilde{x}_t$ and $n \leftarrow n+1$.
12:     **else**
13:       Sample $x_{n+1} \sim (q(\cdot|x_{1:n}) - p(\cdot|x_{1:n}))_+$ and set $n \leftarrow n+1$ and exit for loop.
14:     **end if**
15:   **end for**
16:   If all tokens $\tilde{x}_1, \ldots, \tilde{x}_K$ are accepted, sample extra token $x_{n+1} \sim q(\cdot|x_{1:n})$ and set $n \leftarrow n+1$.
17: **end while**

Definition 35 (acceptance rate)**.** *Given text so far $x_{1:n}$, the* acceptance rate *of Algorithm 4 is the probability of accepting the draft model's token x_{n+1} on line 11:*

$$\textit{acceptance rate} = \sum_{x_{n+1} \in V} p(x_{n+1}|x_{1:n}) \min\left(1, \frac{q(x_{n+1}|x_{1:n})}{p(x_{n+1}|x_{1:n})}\right).$$

Intuitively, the closer p is to q, the higher the acceptance rate is likely to be. A high acceptance rate is desirable as it speeds up the sampling process.

Lastly, we highlight a core property of speculative sampling, which is that it is equivalent to sampling from the target distribution:

Theorem 36 (Speculative sampling is equivalent to target distribution)**.** *The output probability distribution of Algorithm 4 given the prompt $x_1, \ldots, x_t$ is equal to the target distribution $q(\cdot|x_1, \ldots, x_t; k)$.*

Proof. See Chen et al. [5]. □

I.2 Desiderata

We would like to design a *generative watermarking with speculative sampling* algorithm to generate text while applying both speculative sampling and a generative watermarking scheme. Ideally, such an algorithm should satisfy the following desiderata:

1. **Non-distortionary** The generative watermarking with speculative sampling algorithm should have the same non-distortion properties as the underlying generative watermarking scheme (see Supplementary Appendix G).
2. **Preserve acceptance rate** The acceptance rate (the rate at which tokens from the draft LLM are accepted) should be the same for speculative sampling with watermarking and speculative sampling without watermarking.
3. **Preserve watermark detectability** The watermark detection performance should be the same for watermarking with speculative sampling, and watermarking the target LLM without speculative sampling.

In the following sections we provide two *generative watermarking with speculative sampling* algorithms, both of which are non-distortionary. First, we provide a method which preserves watermark detectability, but it may reduce the acceptance rate; we call this algorithm **high-detectability watermarked speculative sampling**. For latency-critical applications where high acceptance rate is important, we provide an alternative method which preserves acceptance rate, but may reduce watermark detectability; we call it **fast watermarked speculative sampling**.

I.3 Compatibility with generative watermarking schemes

Our two algorithms can generally be used with most generative watermarking schemes, with two important caveats:

1. For the 'preserve acceptance rate' property to hold in the **fast watermarked speculative sampling** algorithm, the watermarking scheme's sampling algorithm $\mathcal{S}$ must be *single-token non-distortionary* (Definition 16) – e.g., Gumbel sampling or two-sample Tournament sampling.
2. The **high-detectability watermarked speculative sampling** algorithm requires that the sampling algorithm $\mathcal{S}$ is *vectorisable*; i.e., given any probability distribution p and random seed r, it is possible to directly compute the watermarked probability distribution $\mathbb{P}\left[\mathcal{S}(p, r) = \cdot\right]$. For Tournament sampling, this means that we need to use the vectorised implementation (Supplementary Appendix E).

I.4 High-detectability watermarked speculative sampling

This algorithm uses the straightforward approach of taking Algorithm 4 and replacing the draft distribution and the target distribution with their watermarked versions. The watermark detection method is then the same as for the underlying generative watermarking scheme. We first define some notation, then present the method in Algorithm 5.

Definition 37 . *Given a watermarking sampling algorithm $\mathcal{S} : \triangle V \times \mathcal{R} \to V$ (see Methods Definition 5), a watermarking key $k \in \mathcal{R}$, and a random seed generator f_r (see Methods Section 5.3), we use the following notation to refer to the watermarked versions of the target distribution q and the draft distribution p:*

$$p_{wm}(x_t|x_{<t}; k) := \mathbb{P}\left[\mathcal{S}(p(\cdot|x_{<t}), f_r(x_{<t}, k)) = x_t\right]$$

$$q_{wm}(x_t|x_{<t};k) := \mathbb{P}\left[\mathcal{S}(q(\cdot|x_{<t}), f_r(x_{<t},k)) = x_t\right].$$

Note that Algorithm 5 requires directly computing the probabilities/logits from the watermarked distributions p_{wm} and q_{wm} rather than just sampling from them; this is the reason why $\mathcal{S}$ must be vectorisable (Supplementary Appendix I.3).

Algorithm 5 High-detectability watermarked speculative sampling

```
Given lookahead K, minimum target sequence length T, watermarked target
  model q_wm(·|·;k), watermarked draft model p_wm(·|·;k), initial prompt sequence
  x_1, ..., x_t.
Initialize n ← t.
while n < T do
  for t = 1 : K do
    Sample draft auto-regressively x̃_t ~ p_wm(·|x_{1:n}, x̃_{1:t-1}; k)
  end for
  In parallel, compute K + 1 sets of logits from drafts x̃_1, ..., x̃_K :
  q_wm(·|x_{1:n}; k),  q_wm(·|x_{1:n}, x̃_1; k),  ...,  q_wm(·|x_{1:n}, x̃_{1:K}; k)
  for t = 1 : K do
    Sample r ~ U[0, 1] from a uniform distribution.
    if r < min(1, q_wm(x̃_t|x_{1:n}; k)/p_wm(x̃_t|x_{1:n}; k)) then
      Set x_{n+1} ← x̃_t and n ← n + 1.
    else
      Sample x_{n+1} ~ (q_wm(·|x_{1:n}; k) − p_wm(·|x_{1:n}; k))_+ and set n ← n + 1 and
      exit for loop.
    end if
  end for
  If all tokens x̃_1, ..., x̃_K are accepted, sample extra token x_{n+1} ~ q_wm(·|x_{1:n}; k)
  and set n ← n + 1.
end while
```

I.4.1 Properties

In this section we show that Algorithm 5 preserves watermark detectability and is non-distortionary but decreases acceptance rate. First we establish the following theorem, which says that generating text from Algorithm 5 is equivalent to generating text from the watermarked target LLM without speculative sampling.

Theorem 38 (Algorithm 5 is equivalent to watermarked target distribution)**.** *The output probability distribution of Algorithm 5 given the prompt $x_1, \ldots, x_t$ is equal to the watermarked target distribution $q_{wm}(\cdot|x_1, \ldots, x_t; k)$.*

Proof. Follows from Theorem 36. □

It follows trivially from Theorem 38 that as Algorithm 5 is equivalent to generating text directly from the target LLM q watermarked with $\mathcal{S}$ and f_r, the watermark detection performance is also identical (for any detection method).

It also follows that Algorithm 5 inherits all non-distortion properties of the generative watermarking scheme; in particular, if $\mathcal{S}$ is single-token non-distortionary, then so is Algorithm 5. Furthermore if the generative watermarking scheme is K-sequence non-distortionary (Definition 20), for example by applying repeated context masking, then so is Algorithm 5 (assuming the repeated context masking is applied in the same way).

Theorem 39 (Algorithm 5 has expected acceptance rate $\leq$ speculative sampling without watermarking)**.** *Assume the sampling algorithm $\mathcal{S}$ is single-token non-distortionary (Definition 16). Given $x_{1:n}$, the acceptance rate of Algorithm 5 (speculative sampling with watermarking) on step $n+1$ is, in expectation over the watermarking key k, less than or equal to the acceptance rate for speculative sampling without watermarking (Definition 35).*

Proof. See Supplementary Appendix K.12. □

I.5 Fast watermarked speculative sampling

For this method, we use two watermarking keys: one key k^D for sampling from the draft model and one key k^T for sampling from the target model (and for sampling when the draft tokens are rejected). We show this allows us to preserve acceptance rate, but it weakens watermark detection performance because during detection we must use a scoring function that checks all tokens against both keys (the scoring functions are described in Supplementary Appendix I.5.2). We now introduce some notation then present the algorithm in Algorithm 6.

Definition 40 . *Given a watermarking sampling algorithm $\mathcal{S} : \triangle V \times \mathcal{R} \to V$ (see Methods Definition 5), watermarking keys k^D and k^T, and a random seed generator f_r (see Methods Section 5.3), we use the following notation:*

$$\begin{aligned} p_{wm}(x_t|x_{<t};k^D) &:= \mathbb{P}\left[\mathcal{S}\left(p(\cdot|x_{<t}), f_r(x_{<t}, k^D)\right) = x_t\right] \\ q_{wm}(x_t|x_{<t};k^T) &:= \mathbb{P}\left[\mathcal{S}\left(q(\cdot|x_{<t}), f_r(x_{<t}, k^T)\right) = x_t\right] \\ (q-p)_+^{wm}(x_t|x_{<t};k^T) &:= \mathbb{P}\left[\mathcal{S}\left(\left[q(\cdot|x_{<t}) - p(\cdot|x_{<t})\right]_+, f_r(x_{<t}, k^T)\right) = x_t\right] \end{aligned}$$

where $(\cdot)_+$ is the operator defined in Definition 34.

Note that Algorithm 6 does not require direct computation of the watermarked probabilities p_{wm}, q_{wm} or $(q-p)_+^{\text{wm}}$; it only requires sampling from them. This is why Algorithm 6 does not require $\mathcal{S}$ to be vectorisable (Supplementary Appendix I.3).

I.5.1 Properties

We now show that Algorithm 6 is non-distortionary and preserves acceptance rate.

Algorithm 6 Fast watermarked speculative sampling

Given lookahead K, minimum target sequence length T, auto-regressive target model $q(.|.)$, auto-regressive draft model $p(.|.)$, initial prompt sequence $x_1, \ldots, x_t$, watermarked models $p_{\text{wm}}(\cdot|\cdot; k^D)$, $q_{\text{wm}}(\cdot|\cdot; k^T)$, $(q-p)_+^{\text{wm}}(\cdot|\cdot; k^T)$.
Initialize $n \leftarrow t$.
while $n < T$ **do**
  for $t = 1 : K$ **do**
    Sample draft auto-regressively $\tilde{x}_t \sim p_{\text{wm}}(\cdot|x_{1:n}, \tilde{x}_{1:t-1}; k^D)$
  end for
  In parallel, compute $K+1$ sets of logits from drafts $\tilde{x}_1, \ldots, \tilde{x}_K$: $q(\cdot|x_{1:n}), \quad q(\cdot|x_{1:n}, \tilde{x}_1), \quad \ldots, \quad q(\cdot|x_{1:n}, \tilde{x}_{1:K})$
  for $t = 1 : K$ **do**
    Sample $r \sim U[0,1]$ from a uniform distribution.
    if $r < \min\left(1, q(\tilde{x}_t|x_{1:n})/p\left(\tilde{x}_t|x_{1:n}\right)\right)$ **then**
      Set $x_{n+1} \leftarrow \tilde{x}_t$ and $n \leftarrow n+1$.
    else
      Sample $x_{n+1} \sim (q-p)_+^{\text{wm}}\left(\cdot|x_{1:n}; k^T\right)$, and set $n \leftarrow n+1$ and exit for loop.
    end if
  end for
  If all tokens $\tilde{x}_1, \ldots, \tilde{x}_K$ are accepted, sample extra token $x_{n+1} \sim q_{\text{wm}}(\cdot|x_{1:n}; k^T)$ and set $n \leftarrow n+1$.
end while

Theorem 41 (Algorithm 6 is single-token non-distortionary[4])**.** *Assume the sampling algorithm $\mathcal{S}$ is single-token non-distortionary (Definition 16). Given $x_{1:n}$, let $q'(\cdot|x_{1:n}; k^D, k^T)$ denote the probability distribution of the next token x_{n+1} generated by Algorithm 6 on step $n+1$. For all $x_{n+1} \in V$:*

$$\mathbb{E}_{k^D \sim \textit{Unif}(\mathcal{R}), k^T \sim \textit{Unif}(\mathcal{R})}\left[q'(x_{n+1}|x_{1:n}; k^D, k^T)\right] = q(x_{n+1}|x_{1:n}).$$

Proof. See Supplementary Appendix K.13. □

If the watermarking scheme has a stronger level of non-distortion (e.g. K-sequence non-distortion, Definition 20), for example via repeated context masking, then we can correspondingly extend Theorem 41 to show the same level of non-distortion, in a similar way to Theorem 21.

Theorem 42 (Algorithm 6 preserves acceptance rate)**.** *Assume the sampling algorithm $\mathcal{S}$ is single-token non-distortionary (Definition 16). Given $x_{1:n}$, the acceptance rate of Algorithm 6 (fast speculative sampling with watermarking) is, in expectation over the keys k^D, k^T, equal to the acceptance rate of speculative sampling without watermarking (Definition 35).*

[4] For notational convenience we prove single-token non-distortion in expectation over the watermarking keys k^D, k^T, but we could also prove non-distortion over the corresponding random seeds, which more closely matches Definition 16.

Proof. See Supplementary Appendix K.14. □

I.5.2 Scoring functions

In Algorithm 6, each generated token x_t is watermarked with either the draft key k^D or the target key k^T, but when it comes time to detect the watermark in a piece of text, we do not know which key was used for each token. This necessitates checking each token against both keys, but half of all these checks will follow an 'unwatermarked' distribution; this is the reason why Algorithm 6 has a lower detection performance than watermarking without speculative sampling.

Nevertheless, in this section we provide adaptations of our scoring functions for SynthID-Text presented in Supplementary Appendix A. Similarly to Supplementary Appendix A.1, let $g^D = \{g^D_{t,\ell}\}_{1\leq t\leq T, 1\leq \ell m}$ denote the g-values computed with the draft key k^D and similarly g^T denote the g-values computed with the target key k^T.

(Weighted) Mean

For the (Weighted) Mean Score (Equation (A2)) we simply sum over g^D and g^T:

$$\text{WeightedMeanScore}(x, \alpha) := \frac{1}{2mT} \sum_{\gamma=D,T} \sum_{t=1}^{T} \sum_{\ell=1}^{m} \alpha_\ell \, g^\gamma_{t,\ell}.$$

(Weighted) Frequentist

Similarly for the (Weighted) Frequentist Score (Equation (A5)), we consider the sum $\frac{1}{2T} \sum_{\gamma=D,T} \sum_{t=1}^{T} \sum_{\ell=1}^{m} \alpha_\ell \, g^\gamma_{t,\ell}$, which follows the Normal$(\mu, \frac{\sigma^2}{2T})$ distribution under the null hypothesis, where μ and σ are defined as previously in Supplementary Appendix A.3.1. Thus:

$$p\text{-value} = 1 - \text{CDF}_{\text{Normal}(\mu, \frac{\sigma^2}{2T})} \left(\frac{1}{2T} \sum_{\gamma=D,T} \sum_{t=1}^{T} \sum_{\ell=1}^{m} \alpha_\ell \, g^\gamma_{t,\ell} \right).$$

Bayesian

For the Bayesian approach in Supplementary Appendix A.4, we can replace the posteriors $P(w|g)$ and $P(\neg w|g)$ with $P(w|g^D, g^T)$ and $P(\neg w|g^D, g^T)$ and similarly the likelihoods $P(g|w)$ and $P(g|\neg w)$ with $P(g^D, g^T|w)$ and $P(g^D, g^T|\neg w)$. To compute the BayesianScore (Equation (A6)), we need to derive the likelihoods $P(g^D_{t,\ell}, g^T_{t,\ell}|\neg w)$ and $P(g^D_{t,\ell}, g^T_{t,\ell}|w)$. For the unwatermarked likelihoods, we have independence of the g-values for the two keys, so:

$$P(g^D_{t,\ell}, g^T_{t,\ell}|\neg w) = P(g^D_{t,\ell}|\neg w) P(g^T_{t,\ell}|\neg w) = f_g(g^D_{t,\ell}) f_g(g^T_{t,\ell}).$$

For the watermarked likelihoods, we marginalize over the key k_t used on step t:

$$P(g^D_{t,\ell}, g^T_{t,\ell}|w) = \sum_{\gamma\in D,T} P(g^D_{t,\ell}, g^T_{t,\ell}|k_t = k^\gamma) P(k_t = k^\gamma)$$

Temp.	Spec. sampling, unwatermarked	Fast watermarked speculative sampling + non-distortionary SynthID-Text				No spec. sampling + non-dist. SynthID-Text	
	Acceptance rate ↑	Acceptance rate ↑	Scoring function	TPR@FPR=1% ↑ 200 tokens	TPR@FPR=1% ↑ 400 tokens	TPR@FPR=1% ↑ 200 tokens	TPR@FPR=1% ↑ 400 tokens
0.7	1.486	1.495	Weighted-Mean	14.33 [14.19, 14.47]	34.15 [33.80, 34.49]		
			Bayesian	**54.66** [54.42, 54.90]	**60.35** [59.93, 60.77]	69.64 [69.48, 69.81]	86.64 [86.42, 86.85]
1.0	1.513	1.514	Weighted-Mean	31.62 [31.42, 31.83]	61.89 [61.61, 62.17]		
			Bayesian	**59.10** [58.95, 59.23]	**65.24** [65.02, 65.47]	87.39 [87.29, 87.48]	97.52 [97.47, 97.57]

Table I5: Results for our novel *fast watermarked speculative sampling* algorithm which combines speculative sampling with non-distortionary SynthID-Text. The addition of the watermark does not affect speculative sampling's efficiency (reflected in the acceptance rate). However, the addition of speculative sampling does reduce the detectability of the watermark (measured using true positive rate for fixed false positive rate of 1%). Results are provided with 90% confidence intervals.

$$= P(g^D_{t,\ell}|k_t = k^D) f_g(g^T_{t,\ell}) P(k_t = k^D) + P(g^T_{t,\ell}|k_t = k^T) f_g(g^D_{t,\ell}) \left[1 - P(k_t = k^D)\right].$$

Note that the prior probability $P(k_t = k^D)$ is equal to the fraction of tokens that come from the draft. This can be learned as a latent parameter of the Bayesian scorer, or set based on the empirical acceptance rate of the LLMs. We then factorize $P(g^\gamma_{t,\ell}|w, k_t = k^\gamma)$ similarly to Theorem 6.

I.5.3 Experimental results

We evaluate our fast watermarked speculative sampling algorithm with non-distortionary SynthID-Text, using `Gemma` 7B-IT as the target model and `Gemma` 2B-IT as the smaller draft model which proposes three 'lookahead' tokens at a time.

Table I5 demonstrates the two key features of fast watermarked speculative sampling. First, that it **preserves acceptance rate**: we see that the speculative sampling acceptance rate (and thus overall latency) is very similar with and without watermarking. While we ran our experiment with non-distortionary SynthID-Text, we expect this result would hold for any non-distortionary generative watermark (Theorem 42). Second, that it **does not preserve detectability**: the watermark detectability is less with fast watermarked speculative sampling, than if we apply the same watermark to `Gemma` 7B-IT without speculative sampling.

Lastly, Table I5 also shows that of the adapted scoring functions for fast watermarked speculative sampling presented in Supplementary Appendix I.5.2, the Bayesian scoring function performs substantially better than WeightedMean.

Appendix J Lemmas

Lemma 43 . *For any integer $j \geq 1$, and real numbers a and b:*

$$\sum_{i=1}^{j} \binom{j}{i} \frac{i}{j} a^i b^{j-i} = a(a+b)^{j-1}.$$

Proof. First note that:

$$\binom{j}{i}\frac{i}{j} = \frac{j!}{i!(j-i)!}\frac{i}{j} = \frac{(j-1)!}{(i-1)!(j-i)!} = \binom{j-1}{i-1}.$$

Then using the binomial formula for the last equality:

$$\sum_{i=1}^{j} \binom{j}{i}\frac{i}{j} a^i b^{j-i} = a\sum_{i=1}^{j}\binom{j-1}{i-1} a^{i-1} b^{j-i} = a\sum_{i=0}^{j-1}\binom{j-1}{i} a^i b^{j-1-i} = a(a+b)^{j-1}.$$

□

Lemma 44 (Upper bound for sum of cubed probabilities)**.** *For any probability distribution $(p_i)_{i=1}^N$:*

$$\sum_{i=1}^{N} p_i^3 \leq \frac{1}{2}\left(\sum_{i=1}^{N} p_i^2\right)\left(1 + \sum_{i=1}^{N} p_i^2\right)$$

with equality iff $(p_i)_{i=1}^N$ is one-hot.

Proof. Note that for all $1 \leq i \leq N$:

$$1 + \sum_{j=1}^{N} p_j^2 \geq 1 + p_i^2 = (1-p_i)^2 + 2p_i \geq 2p_i,$$

with equality iff $p_i = 1$. Therefore

$$\sum_{i=1}^{N} p_i^3 \leq \sum_{i=1}^{N} p_i^2 \frac{1}{2}\left(1 + \sum_{j=1}^{N} p_j^2\right) = \frac{1}{2}\left(\sum_{i=1}^{N} p_i^2\right)\left(1 + \sum_{i=1}^{N} p_i^2\right)$$

with equality iff $p_i = 0$ or $p_i = 1$ for all i. □

Lemma 45 (Lower bound for sum of cubed probabilities)**.** *For any probability distribution $(p_i)_{i=1}^N$:*

$$\sum_{i=1}^{N} p_i^3 \geq \frac{3}{2}\sum_{i=1}^{n} p_i^2 - \frac{1}{2}.$$

Proof. By induction on N. For the base case $N = 1$, LHS $= 1$ and RHS $= \frac{3}{2} - \frac{1}{2} = 1$. Now suppose the statement is true for $N - 1$. Then

$$\begin{aligned}
\sum_{i=1}^{N} p_i^3 &= (1 - p_N)^3 \sum_{i=1}^{N-1} \left(\frac{p_i}{1 - p_N} \right)^3 + p_N^3 \\
&\geq (1 - p_N)^3 \left[\frac{3}{2} \sum_{i=1}^{N-1} \left(\frac{p_i}{1 - p_N} \right)^2 - \frac{1}{2} \right] + p_N^3 && \text{(induction assumption)} \\
&= \frac{3}{2}(1 - p_N) \sum_{i=1}^{N-1} p_i^2 - \frac{1}{2}(1 - p_N)^3 + p_N^3 && \text{(rearrange)} \\
&= \frac{3}{2} \sum_{i=1}^{N-1} p_i^2 - \frac{3}{2} p_N \sum_{i=1}^{N-1} p_i^2 - \frac{1}{2} + \frac{3}{2} p_N - \frac{3}{2} p_N^2 + \frac{3}{2} p_N^3 && \text{(rearrange)} \\
&= \frac{3}{2} \sum_{i=1}^{N} p_i^2 - \frac{1}{2} - \frac{3}{2} p_N \sum_{i=1}^{N} p_i^2 + \frac{3}{2} p_N - 3p_N^2 + 3p_N^3. && \text{(rearrange)}
\end{aligned}$$

Note that $\sum_{i=1}^{N} p_i^2 \leq p_N^2 + (1 - p_N)^2 = 1 - 2p_N + 2p_N^2$, so:

$$\begin{aligned}
\sum_{i=1}^{N} p_i^3 &\geq \frac{3}{2} \sum_{i=1}^{N} p_i^2 - \frac{1}{2} - \frac{3}{2} p_N \left(1 - 2p_N + 2p_N^2\right) + \frac{3}{2} p_N - 3p_N^2 + 3p_N^3 \\
&= \frac{3}{2} \sum_{i=1}^{N} p_i^2 - \frac{1}{2}.
\end{aligned}$$

□

Appendix K Proofs

K.1 Proof of Theorem 6

Proof. For the unwatermarked case $P(g|\neg w)$, the g-values $\{g_{t,\ell}\}_{1 \leq t \leq T, 1 \leq \ell \leq m}$ are independent across timesteps t and across layers ℓ. Furthermore, each $g_{t,\ell}$ follows the (unwatermarked) g-value distribution with p.d.f/p.m.f. f_g, thus:

$$\begin{aligned}
P(g|\neg w) &= \prod_{t=1}^{T} \prod_{\ell=1}^{m} P(g_{t,\ell}|\neg w) \\
&= \prod_{t=1}^{T} \prod_{\ell=1}^{m} f_g(g_{t,\ell}).
\end{aligned}$$

For the watermarked case $P(g|w)$, we assume the g-values are independent across timesteps t but not across layers ℓ:

$$P(g|w) = \prod_{t=1}^{T} \prod_{\ell=1}^{m} P(g_{t,\ell}|w, g_{t,<\ell}).$$

To compute $P(g_{t,\ell}|w, g_{t,<\ell})$, we introduce and marginalize over a latent variable $\psi_{t,\ell} \in \{1, \dots, N\}$ which represents the number of unique candidate tokens in a tournament 'match' at layer ℓ, on timestep t:

$$P(g_{t,\ell}|w, g_{t,<\ell}) = \sum_{c=1}^{N} P(g_{t,\ell}|\psi_{t,\ell} = c) P(\psi_{t,\ell} = c|g_{t,<\ell}).$$

Next, the distribution $P(g_{t,\ell}|\psi_{t,\ell} = c)$ is equal to the distribution of the maximum of c i.i.d. samples from f_g, which can be shown to equal:

$$P(g_{t,\ell}|\psi_{t,\ell} = c) = \begin{cases} cF_g(g_{t,\ell})^{c-1} f_g(g_{t,\ell}) & \text{if } f_g \text{ is continuous} \\ F_g(g_{t,\ell})^c - [F_g(g_{t,\ell}) - f_g(g_{t,\ell})]^c & \text{if } f_g \text{ is discrete.} \end{cases}$$

□

K.2 Proof of Theorem 11

Proof. In this proof we refer to Methods Algorithm 1 for single layer Tournament sampling. First note that if $p(x_t) = 0$ then $\mathbb{P}(\text{Alg 1 returns } x_t) = 0$; the rest of this proof assumes $p(x_t) \neq 0$.

$$\begin{aligned} &\mathbb{P}(\text{Alg 1 returns } x_t) \\ &= \sum_{j=1}^{N} \sum_{i=1}^{j} \mathbb{P}(|Y^*| = j,\ x_t \text{ appears } i \text{ times in } Y^*, \text{Alg 1 returns } x_t) \\ &= \sum_{j=1}^{N} \sum_{i=1}^{j} \binom{N}{j} p(V^{<g_1(x_t,r)})^{N-j} \binom{j}{i} p(x_t)^i p(V^{=g_1(x_t,r)} \setminus x_t)^{j-i} \frac{i}{j} \\ &= \sum_{j=1}^{N} \binom{N}{j} p(V^{<g_1(x_t,r)})^{N-j} \sum_{i=1}^{j} \binom{j}{i} \frac{i}{j} p(x_t)^i p(V^{=g_1(x_t,r)} \setminus x_t)^{j-i}. \qquad \text{(rearrange)} \end{aligned}$$

Now note that, by application of Lemma 43:

$$\begin{aligned} \sum_{i=1}^{j} \binom{j}{i} \frac{i}{j} p(x_t)^i p(V^{=g_1(x_t,r)} \setminus x_t)^{j-i} &= p(x_t) \left[p(x_t) + p(V^{=g_1(x_t,r)} \setminus x_t) \right]^{j-1} && \text{(Lemma 43)} \\ &= p(x_t)\ \ p(V^{=g_1(x_t,r)})^{j-1}. && \text{(simplify)} \end{aligned}$$

Substituting this back in:

$$\begin{aligned}
&\mathbb{P}(\text{Alg 1 returns } x_t) \\
&= \sum_{j=1}^{N} \binom{N}{j} p(V^{<g_1(x_t,r)})^{N-j} p(x_t) p(V^{=g_1(x_t,r)})^{j-1} \\
&= \frac{p(x_t)}{p(V^{=g_1(x_t,r)})} \sum_{j=1}^{N} \binom{N}{j} p(V^{<g_1(x_t,r)})^{N-j} p(V^{=g_1(x_t,r)})^{j} && \text{(rearrange)} \\
&= \frac{p(x_t)}{p(V^{=g_1(x_t,r)})} \left(\left[p(V^{<g_1(x_t,r)}) + p(V^{=g_1(x_t,r)}) \right]^N - p(V^{<g_1(x_t,r)})^N \right) && \text{(binomial formula)} \\
&= \frac{p(x_t)}{p(V^{=g_1(x_t,r)})} \left(p(V^{\leq g_1(x_t,r)})^N - p(V^{<g_1(x_t,r)})^N \right). && \text{(simplify)}
\end{aligned}$$

□

K.3 Proof of computational complexities

Tournament sampling

The tournament-based implementation of multi-layer Tournament sampling presented in Methods Algorithm 2 requires N^m samples to be taken from $p_{\text{LM}}(\cdot|x_{<t})$ and $N^m - 1$ comparison operations to decide the winners of the matches. The number of g-values to be computed is at most $N^m + N^{m-1} + \cdots + N = N^{m+1} - N$ (if you compute the g-values for all candidates in the tournament) or $m|V|$ (if you compute g-values for all tokens in the vocabulary for every layer).

Vectorised tournament, general

The general vectorised implementation of Tournament sampling presented in Theorem 15 requires m applications of Equation (E21). Equation (E21) requires the computation of $p(V^{<g(x_t)})$ and $p(V^{=g(x_t)})$ for each $x_t \in V$; this can be computed in $O(|V| \log |V|)$ operations by first sorting the g-values. The number of g-values to be computed is $m|V|$, and only one sample needs to be taken at the end of the process.

Vectorised tournament, binary g-values

In the special case of binary g-values (which we use in most of our experiments, with a Bernoulli g-value distribution), each layer only requires the computation of $p(V^{g_1=0})$ and $p(V^{g_1=1})$ (see Corollary 14), thus no sort is required and the number of operations is $O(|V|)$ per layer.

Gumbel sampling

Gumbel sampling (Supplementary Appendix B.1.1) requires us to compute $|V|$ g-values – i.e., U_i in Equation (B12). We then need to compute $-\frac{p(x_i)}{\log(U_i)}$ for every $x_i \in V$ then take the argmin, which requires $O(|V|)$ operations.

Soft Red List sampling

Soft Red List sampling (Supplementary Appendix B.1.2) requires us to compute $|V|$ (binary) g-values. Adding a constant to all logits on the green list and taking softmax requires $O(|V|)$ operations, then finally we take a single sample from p_{wm}.

K.4 Proof of Theorem 17

Proof. Equation (E16) gives an expression for $p_{\text{wm}}(x_t|p, r, f_g, 2, 1)$ which we can rewrite:

$$\begin{aligned}
p_{\text{wm}}(x_t|p,r,f_g,2,1) &= p(x_t)\left(p(V^{=g_1(x_t,r)}) + 2p(V^{<g_1(x_t,r)})\right) && \text{(Eqn E16)}\\
&= p(x_t)\left(\sum_{x\in V} p(x)\left[\mathbb{1}_{g_1(x,r)=g_1(x_t,r)} + 2\mathbb{1}_{g_1(x,r)<g_1(x_t,r)}\right]\right) && \text{(rearrange)}
\end{aligned}$$

Next observe that for any $x, x_t \in V$ (here for conciseness we write $\mathbb{E}_r$ to mean $\mathbb{E}_{r\sim\text{Unif}(\mathcal{R})}$):

$$\begin{aligned}
&\mathbb{E}_r\left[\mathbb{1}_{g_1(x,r)=g_1(x_t,r)}\right] + 2\mathbb{E}_r\left[\mathbb{1}_{g_1(x,r)<g_1(x_t,r)}\right]\\
=&\mathbb{E}_r\left[\mathbb{1}_{g_1(x,r)=g_1(x_t,r)}\right] + \mathbb{E}_r\left[\mathbb{1}_{g_1(x,r)<g_1(x_t,r)}\right] + \mathbb{E}_r\left[\mathbb{1}_{g_1(x,r)>g_1(x_t,r)}\right] && \text{(by Methods Def 4)}\\
=&\mathbb{E}_r\left[\mathbb{1}_{g_1(x,r)=g_1(x_t,r)} + \mathbb{1}_{g_1(x,r)<g_1(x_t,r)} + \mathbb{1}_{g_1(x,r)>g_1(x_t,r)}\right]\\
=&\mathbb{E}_r[1]\\
=&1.
\end{aligned}$$

Substituting back:

$$\begin{aligned}
\mathbb{E}_r\left[p_{\text{wm}}(x_t|p,r,f_g,2,1)\right] &= p(x_t)\left(\sum_{x\in V} p(x)\right)\\
&= p(x_t).
\end{aligned}$$

□

K.5 Proof of Theorem 18

Proof. Proof by induction. The $m = 1$ base case is given by Theorem 17. For the induction case, suppose Equation (G23) is true for $m-1$. From Theorem 15, we know $p_{\text{wm}}(\cdot|p, r_t, f_g, 2, m) = W(p_{\text{wm}}^{(m-1)}, g_m(\cdot, r_t), 2)$ where $p_{\text{wm}}^{(m-1)} = p_{\text{wm}}(\cdot|p, r_t, f_g, 2, m-1)$ is the watermarked distribution for $m-1$ layers. So:

$$\begin{aligned}
&\mathbb{E}_{r_t\sim\text{Unif}(\mathcal{R})}\left[p_{\text{wm}}(x_t|p,r_t,f_g,2,m)\right]\\
=&\mathbb{E}_{r_t\sim\text{Unif}(\mathcal{R})}\left[W(p_{\text{wm}}(\cdot|p,r_t,f_g,2,m-1), g_m(\cdot,r_t),2)\right].
\end{aligned}$$

Now consider that $p_{\mathrm{wm}}(\cdot|p, r_t, f_g, 2, m-1)$ depends on r_t only via the $g_\ell(\cdot, r_t)$ values for $\ell = 1, \ldots, m-1$. Because of our definition of g-values using a pseudorandom hash function (Methods Definition 4), we can separate the expectation for different layers:

$$
\begin{aligned}
=&\mathbb{E}_{r_t\sim\mathrm{Unif}(\mathcal{R})}\left[\mathbb{E}_{r_t'\sim\mathrm{Unif}(\mathcal{R})}\left[W(p_{\mathrm{wm}}(\cdot|p, r_t, f_g, 2, m-1), g_m(\cdot, r_t'), 2)\right]\right] \\
=&\mathbb{E}_{r_t\sim\mathrm{Unif}(\mathcal{R})}\left[p_{\mathrm{wm}}(\cdot|p, r_t, f_g, 2, m-1)\right] && \text{(Thm 17)} \\
=&p(x_t). && \text{(induction assumption)}
\end{aligned}
$$

881 □

882 K.6 Proof of Theorem 19

Proof. Consider the family of probability distributions over a two-word vocabulary $V = \{a, b\}$ with $p_{\mathrm{LM}}(a) = p$ and $p_{\mathrm{LM}}(b) = 1-p$ for some $p \in [0, 1]$. Then by considering the cases where a appears i times in the N samples, we can write:

$$
\begin{aligned}
&\mathbb{E}_r\left[p_{\mathrm{wm}}(a|p_{\mathrm{LM}}, r, f_g, N, 1)\right] \\
=&\mathbb{E}_r\left[p^N + \sum_{i=1}^{N-1}\binom{N}{i}p^i(1-p)^{N-i}\left[\mathbb{1}_{g_1(a,r)>g_1(b,r)} + \mathbb{1}_{g_1(a,r)=g_1(b,r)}\frac{i}{N}\right]\right] \\
=&p^N + \sum_{i=1}^{N-1}\binom{N}{i}p^i(1-p)^{N-i}\left[\frac{1-C_{f_g}}{2} + C_{f_g}\frac{i}{N}\right], \qquad \text{(K35)}
\end{aligned}
$$

where C_{f_g} is the collision probability of f_g. Expression K35 is a polynomial in p of degree $\leq N$. If the sampling algorithm is non-distortionary, then this polynomial equals $p_{\mathrm{LM}}(a) = p$ for all $p \in [0, 1]$, so the polynomial coefficients must be zero for all powers other than p^1. However, consider the coefficient of p^2:

$$
\begin{aligned}
&\sum_{i=1}^{2}\binom{N}{i}\binom{N-1}{2-i}(-1)^{2-i}\left[\frac{1-C_{f_g}}{2} + C_{f_g}\frac{i}{N}\right] \\
=&-N(N-1)\left[\frac{1-C_{f_g}}{2} + C_{f_g}\frac{1}{N}\right] + \frac{N(N-1)}{2}\left[\frac{1-C_{f_g}}{2} + C_{f_g}\frac{2}{N}\right] \\
=&\frac{N(N-1)}{4}\left[C_{f_g} - 1\right].
\end{aligned}
$$

883 This is non-zero as $N > 2$ and $C_{f_g} \neq 1$. Proof by contradiction. □

884 K.7 Proof of Theorem 21

885 *Proof.* In Methods Algorithm 3, each response $\mathbf{y}^i$ is in fact a continuation of its cor-
886 responding prompt $\mathbf{x}^i$. Therefore we write $\mathbf{y}_i = \mathbf{x}^i_{n_i+1}, \ldots, \mathbf{x}^i_{T_i}$ where n_i is the length
887 of prompt $\mathbf{x}^i = \mathbf{x}^i_1, \ldots, \mathbf{x}^i_{n_i}$.

Now, each $\mathbb{P}_{\mathrm{wm}}\left(\mathbf{y}^i|\mathbf{x}^i, k; (\mathbf{x}^1, \mathbf{y}^1), \ldots, (\mathbf{x}^{i-1}, \mathbf{y}^{i-1})\right)$ can be written as a product $\prod_{t=n_i+1}^{T_i}\mathbb{P}_{\mathrm{wm}}\left(\mathbf{x}^i_t|\mathbf{x}^i_{<t}, k; (\mathbf{x}^1, \mathbf{y}^1), \ldots, (\mathbf{x}^{i-1}, \mathbf{y}^{i-1})\right)$. Let W_i denote the set of all

timesteps $t = n_i+1, \ldots, T_i$ for which the context window $\mathbf{x}^i_{t-H:t-1} := (\mathbf{x}^i_{t-H}, \ldots, \mathbf{x}^i_{t-1})$ is already in the context history $C_1 \cup C_2 \cup \cdots \cup C_i$ (see line 6 in Methods Algorithm 3). Thus:

$$\begin{aligned}&\mathbb{P}_{\text{wm}}\left(\mathbf{x}^i_t | \mathbf{x}^i_{<t}, k; (\mathbf{x}^1, \mathbf{y}^1), \ldots, (\mathbf{x}^{i-1}, \mathbf{y}^{i-1})\right)\\ &= \begin{cases} p_{\text{LM}}(\mathbf{x}^i_t | \mathbf{x}^i_{<t}) & \text{if } t \in W_i \\ \mathbb{P}\left[\mathcal{S}\left(p_{\text{LM}}(\cdot|\mathbf{x}^i_{<t}), h(\mathbf{x}^i_{t-H:t-1}, k)\right) = \mathbf{x}^i_t\right] & \text{otherwise.}\end{cases}\end{aligned}$$

Thus:

$$\begin{aligned}&\mathbb{E}_{k\sim\text{Unif}(\mathcal{R})}\left[\prod_{i=1}^{K} \mathbb{P}_{\text{wm}}\left(\mathbf{y}^i | \mathbf{x}^i, k; (\mathbf{x}^1, \mathbf{y}^1), \ldots, (\mathbf{x}^{i-1}, \mathbf{y}^{i-1})\right)\right]\\ =&\mathbb{E}_{k\sim\text{Unif}(\mathcal{R})}\left[\prod_{i=1}^{K}\prod_{t=n_i+1}^{T_i} \mathbb{P}_{\text{wm}}\left(\mathbf{x}^i_t | \mathbf{x}^i_{<t}, k; (\mathbf{x}^1, \mathbf{y}^1), \ldots, (\mathbf{x}^{i-1}, \mathbf{y}^{i-1})\right)\right]\\ =&\mathbb{E}_{k\sim\text{Unif}(\mathcal{R})}\left[\prod_{i=1}^{K}\prod_{t\in W_i} p_{\text{LM}}(\mathbf{x}^i_t|\mathbf{x}^i_{<t}) \prod_{t\notin W_i} \mathbb{P}\left[\mathcal{S}\left(p_{\text{LM}}(\cdot|\mathbf{x}^i_{<t}), h(\mathbf{x}^i_{t-H:t-1}, k)\right) = \mathbf{x}^i_t\right]\right]\end{aligned}$$

Note that this product depends on k only through $h(\mathbf{x}^i_{t-H:t-1}, k)$, where all $\mathbf{x}^i_{t-H:t-1}$ terms are different. By pseudorandom definition of h (Methods Section 5.3), taking expectation $\mathbb{E}_{k\sim\text{Unif}(\mathcal{R})}$ over the whole product is equivalent to taking separate expectations over the random seed produced by h:

$$\begin{aligned}&=\prod_{i=1}^{K}\prod_{t\in W_i} p_{\text{LM}}(\mathbf{x}^i_t|\mathbf{x}^i_{<t}) \prod_{t\notin W_i} \mathbb{E}_{r\sim\text{Unif}(\mathcal{R})}\left(\mathbb{P}\left[\mathcal{S}\left(p_{\text{LM}}(\cdot|\mathbf{x}^i_{<t}), r\right) = \mathbf{x}^i_t\right]\right)\\ &=\prod_{i=1}^{K}\prod_{t\in W_i} p_{\text{LM}}(\mathbf{x}^i_t|\mathbf{x}^i_{<t}) \prod_{t\notin W_i} p_{\text{LM}}(\mathbf{x}^i_t|\mathbf{x}^i_{<t}) \qquad (\mathcal{S}\text{ non-distortionary})\\ &=\prod_{i=1}^{K}\prod_{t=n_i+1}^{T_i} p_{\text{LM}}(\mathbf{x}^i_t|\mathbf{x}^i_{<t})\\ &=\prod_{i=1}^{K} p_{\text{LM}}(\mathbf{y}^i|\mathbf{x}^i).\end{aligned}$$

888 □

889 K.8 Proof of Theorem 25

Proof. We can divide $F_{gw}(z)$ by how many unique samples there are in the N samples drawn from p_{LM} in Methods Algorithm 1:

$$F_{gw}(z) = \mathbb{P}_{r\sim\text{Unif}(\mathcal{R}), x\sim p_{\text{wm}}(\cdot|p,r,f_g,N,1)}\left[g_1(x,r) \leq z\right] \qquad \text{(Definition 24)}$$

$$= \sum_{j=1}^{N} C_{p_{\mathrm{LM}}}^{N,j} \mathbb{P}_{r\sim\mathrm{Unif}(\mathcal{R})} \left[j \text{ unique } y_1, \ldots, y_j \text{ all have } g_1(y_i, r) \le z \right]$$

$$= \sum_{j=1}^{N} C_{p_{\mathrm{LM}}}^{N,j} F_g(z)^j. \qquad \text{(Methods Definition 3)}$$

Next, if f_g is continuous, then:

$$f_{gw}(z) = \frac{d}{dz} F_{gw}(z)$$

$$= f_g(z) \sum_{j=1}^{N} C_{p_{\mathrm{LM}}}^{N,j} j F_g(z)^{j-1}. \qquad \text{(chain rule)}$$

Lastly, if f_g is is discrete with support $z_1 < z_2 < \cdots < z_L$, then for each z_i:

$$f_{gw}(z_i) = F_{gw}(z_i) - F_{gw}(z_{i-1}) \qquad \text{(let } F_{gw}(z_0) = 0.)$$

$$= \sum_{j=1}^{N} C_{p_{\mathrm{LM}}}^{N,j} F_g(z_i)^j - \sum_{j=1}^{N} C_{p_{\mathrm{LM}}}^{N,j} F_g(z_{i-1})^j \qquad \text{(shown above)}$$

$$= \sum_{j=1}^{N} C_{p_{\mathrm{LM}}}^{N,j} \left(F_g(z_i)^j - [F_g(z_i) - f_g(z_i)]^j \right) \qquad \text{(rearrange)}$$

$$= \sum_{j=1}^{N} C_{p_{\mathrm{LM}}}^{N,j} \left(\sum_{k=1}^{j} (-1)^{k-1} \binom{j}{k} F_g(z_i)^{j-k} f_g(z_i)^k \right) \qquad \text{(binomial formula)}$$

$$= f_g(z_i) \sum_{j=1}^{N} C_{p_{\mathrm{LM}}}^{N,j} \left(\sum_{k=1}^{j} (-1)^{k-1} \binom{j}{k} F_g(z_i)^{j-k} f_g(z_i)^{k-1} \right).$$

□

K.9 Proof of Theorem 29

Proof. From Equation (H24),

$$F_{gw}^{N+1}(z) = \sum_{j=1}^{N+1} C_{p_{\mathrm{LM}}}^{N+1,j} F_g(z)^j.$$

Note that $C_{p_{\mathrm{LM}}}^{N+1,j}$ can be written as:

$$C_{p_{\mathrm{LM}}}^{N+1,j} = C_{p_{\mathrm{LM}}}^{N,j} \mathbb{P}_{p_{\mathrm{LM}}}^{N,j}(\text{same}) + C_{p_{\mathrm{LM}}}^{N,j-1} \mathbb{P}_{p_{\mathrm{LM}}}^{N,j-1}(\text{new})$$

where $\mathbb{P}_{p_{\mathrm{LM}}}^{N,j}(\text{same})$ is the probability that an additional sample from p_{LM} is already in a collection Y of N samples sampled i.i.d. from p_{LM}, given that Y contains j unique

elements. Similarly $\mathbb{P}^{N,j-1}_{p_{\text{LM}}}(\text{new})$ is the probability that an additional sample from p_{LM} is not already in the collection Y of N samples sampled from p_{LM}, given that Y contains $j-1$ unique elements.

Now, we can substitute this in:

$$\begin{aligned}
F^{N+1}_{gw}(z) &= \sum_{j=1}^{N+1} \left[C^{N,j}_{p_{\text{LM}}}\mathbb{P}^{N,j}_{p_{\text{LM}}}(\text{same}) + C^{N,j-1}_{p_{\text{LM}}}\mathbb{P}^{N,j-1}_{p_{\text{LM}}}(\text{new})\right] F_g(z)^j \\
&= \sum_{j=1}^{N} C^{N,j}_{p_{\text{LM}}}\left[\mathbb{P}^{N,j}_{p_{\text{LM}}}(\text{same})F_g(z)^j + \mathbb{P}^{N,j}_{p_{\text{LM}}}(\text{new})F_g(z)^{j+1}\right] && \text{(reindex)} \\
&= \sum_{j=1}^{N} C^{N,j}_{p_{\text{LM}}}\left[\mathbb{P}^{N,j}_{p_{\text{LM}}}(\text{same}) + \mathbb{P}^{N,j}_{p_{\text{LM}}}(\text{new})F_g(z)\right] F_g(z)^j && \text{(rearrange)} \\
&= \sum_{j=1}^{N} C^{N,j}_{p_{\text{LM}}}\left[1 - (1-F_g(z))\mathbb{P}^{N,j}_{p_{\text{LM}}}(\text{new})\right] F_g(z)^j && (\mathbb{P}(\text{new}) + \mathbb{P}(\text{same}) = 1) \\
&= F^{N}_{gw}(z) - (1-F_g(z))\sum_{j=1}^{N}\mathbb{P}^{N,j}_{p_{\text{LM}}}(\text{new})C^{N,j}_{p_{\text{LM}}}F_g(z)^j && \text{(Eqn H24)} \\
&\geq F^{N}_{gw}(z).
\end{aligned}$$

When $0 < F^N_{gw}(z) < 1$, the equality holds iff $\mathbb{P}^{N,j}_{p_{\text{LM}}}(\text{new})C^{N,j}_{p_{\text{LM}}} = 0$ for all $j = 1, \ldots, N$; equivalently iff the support of $p_{\text{LM}}(\cdot|x_{<t})$ has j or fewer elements for all $j = 1, \ldots, N$. This is true iff $p_{\text{LM}}(\cdot|x_{<t})$ is one-hot. □

K.10 Proof of Theorem 31

Proof. For conciseness, we will write $g(x)$ to mean $g_1(x, r)$. From Equation (E16):

$$\begin{aligned}
p_{\text{wm}}(x|p_{\text{LM}}, r, f_g, 2, 1) &= p_{\text{LM}}(x)\left[p_{\text{LM}}(V^{=g(x)}) + 2p_{\text{LM}}(V^{<g(x)})\right] \\
&= p_{\text{LM}}(x)\left[p_{\text{LM}}(x) + \sum_{x'\in V, x'\neq x} p_{\text{LM}}(x')\left(\mathbb{1}_{g(x')=g(x)} + 2\mathbb{1}_{g(x')<g(x)}\right)\right].
\end{aligned}$$

So the collision probability $C^{2,1}_{p_{\text{wm}}} = \sum_{x\in V} p_{\text{wm}}(x|p_{\text{LM}}, r, f_g, 2, 1)^2$ equals:

$$C^{2,1}_{p_{\text{wm}}} = \sum_{x\in V} p_{\text{LM}}(x)^2\left[p_{\text{LM}}(x) + \sum_{x'\in V, x'\neq x} p_{\text{LM}}(x')\left(\mathbb{1}_{g(x')=g(x)} + 2\mathbb{1}_{g(x')<g(x)}\right)\right]^2.$$

Expanding this out, it can be written as $C^{2,1}_{p_{\text{wm}}} = A + B + C + D$ where:

$$A = \sum_{x\in V} p_{\text{LM}}(x)^4$$

$$B = 2\sum_{x\in V} p_{\text{LM}}(x)^3 \sum_{x'\in V, x'\neq x} p_{\text{LM}}(x')\left(\mathbb{1}_{g(x')=g(x)} + 2\mathbb{1}_{g(x')<g(x)}\right)$$
$$C = \sum_{x\in V} p_{\text{LM}}(x)^2 \sum_{x'\in V, x'\neq x} p_{\text{LM}}(x')^2\left(\mathbb{1}_{g(x')=g(x)} + 2\mathbb{1}_{g(x')<g(x)}\right)^2$$
$$D = \sum_{x\in V} p_{\text{LM}}(x)^2 \sum_{\substack{x_1,x_2\in V,\\ x_1\neq x, x_2\neq x, x_1\neq x_2}} p_{\text{LM}}(x_1)p_{\text{LM}}(x_2)\left(\mathbb{1}_{g(x_1)=g(x)} + 2\mathbb{1}_{g(x_1)<g(x)}\right)\left(\mathbb{1}_{g(x_2)=g(x)} + 2\mathbb{1}_{g(x_2)<g(x)}\right)$$

Tackling these individually, first we have $A = C^{4,1}_{p_{\text{LM}}}$. Now B: for $x' \neq x$:

$$\mathbb{E}_r\left[\mathbb{1}_{g(x')=g(x)} + 2\mathbb{1}_{g(x')<g(x)}\right] = \mathbb{E}_r\left[\mathbb{1}_{g(x')=g(x)} + \mathbb{1}_{g(x')<g(x)} + \mathbb{1}_{g(x')>g(x)}\right] = 1$$

so:

$$\mathbb{E}_r[B] = 2\sum_{x\in V} p_{\text{LM}}(x)^3 \sum_{x'\in V, x'\neq x} p_{\text{LM}}(x') = 2\sum_{x\in V} p_{\text{LM}}(x)^3(1 - p_{\text{LM}}(x)) = 2C^{3,1}_{p_{\text{LM}}} - 2C^{4,1}_{p_{\text{LM}}}.$$

Next C:

$$\begin{aligned}\mathbb{E}_r\left[\left(\mathbb{1}_{g(x')=g(x)} + 2\mathbb{1}_{g(x')<g(x)}\right)^2\right] &= \mathbb{E}_r\left[\mathbb{1}_{g(x')=g(x)} + 4\mathbb{1}_{g(x')<g(x)}\right]\\ &= C^{2,1}_{f_g} + 4\frac{1 - C^{2,1}_{f_g}}{2}\\ &= 2 - C^{2,1}_{f_g}.\end{aligned}$$

and so:

$$\begin{aligned}\mathbb{E}_r[C] &= \sum_{x\in V} p_{\text{LM}}(x)^2 \sum_{x'\in V, x'\neq x} p_{\text{LM}}(x')^2\left(2 - C^{2,1}_{f_g}\right)\\ &= \left(2 - C^{2,1}_{f_g}\right)\sum_{x\in V} p_{\text{LM}}(x)^2(C^{2,1}_{p_{\text{LM}}} - p_{\text{LM}}(x)^2)\\ &= \left(2 - C^{2,1}_{f_g}\right)(C^{2,1}_{p_{\text{LM}}})^2 - \left(2 - C^{2,1}_{f_g}\right)C^{4,1}_{p_{\text{LM}}}.\end{aligned}$$

Lastly for D, note that for $x_1 \neq x$, $x_2 \neq x$, $x_1 \neq x_2$:

$$\begin{aligned}&\mathbb{E}_r\left[\left(\mathbb{1}_{g(x_1)=g(x)} + 2\mathbb{1}_{g(x_1)<g(x)}\right)\left(\mathbb{1}_{g(x_2)=g(x)} + 2\mathbb{1}_{g(x_2)<g(x)}\right)\right]\\ =&\mathbb{E}_r\left[\mathbb{1}_{g(x_1)=g(x_2)=g(x)} + 2\mathbb{1}_{g(x_1)<g(x_2)=g(x)} + 2\mathbb{1}_{g(x_2)<g(x_1)=g(x)} + 4\mathbb{1}_{g(x_1)<g(x),g(x_2)<g(x)}\right]\\ =&C^{3,1}_{f_g} + 2\frac{C^{3,2}_{f_g}}{3\times 2} + 2\frac{C^{3,2}_{f_g}}{3\times 2} + 4\left(\frac{C^{3,2}_{f_g}}{3\times 2} + \frac{C^{3,3}_{f_g}}{3}\right)\\ =&C^{3,1}_{f_g} + \frac{4}{3}C^{3,2}_{f_g} + \frac{4}{3}C^{3,3}_{f_g}\\ =&\frac{4}{3} - \frac{1}{3}C^{3,1}_{f_g}\end{aligned}$$

where the last equality is because because $C_{f_g}^{3,1} + C_{f_g}^{3,2} + C_{f_g}^{3,3} = 1$. Also note that:

$$\begin{aligned}\sum_{\substack{x_1, x_2 \in V, \\ x_1 \neq x, x_2 \neq x, x_1 \neq x_2}} p_{\text{LM}}(x_1) p_{\text{LM}}(x_2) &= \sum_{\substack{x_1 \in V: \\ x_1 \neq x}} p_{\text{LM}}(x_1) \left(1 - p_{\text{LM}}(x) - p_{\text{LM}}(x_1)\right) \\ &= (1 - p_{\text{LM}}(x))^2 - C_{p_{\text{LM}}}^{2,1} + p_{\text{LM}}(x)^2 \\ &= 1 - C_{p_{\text{LM}}}^{2,1} - 2p_{\text{LM}}(x) + 2p_{\text{LM}}(x)^2.\end{aligned}$$

And so:

$$\begin{aligned}\mathbb{E}_r[D] &= \sum_{x \in V} p_{\text{LM}}(x)^2 \left(1 - C_{p_{\text{LM}}}^{2,1} - 2p_{\text{LM}}(x) + 2p_{\text{LM}}(x)^2\right) \left(\frac{4}{3} - \frac{1}{3} C_{f_g}^{3,1}\right) \\ &= \left(\frac{4}{3} - \frac{1}{3} C_{f_g}^{3,1}\right) \left[\left(1 - C_{p_{\text{LM}}}^{2,1}\right) \sum_{x \in V} p_{\text{LM}}(x)^2 - 2 \sum_{x \in V} p_{\text{LM}}(x)^3 + 2 \sum_{x \in V} p_{\text{LM}}(x)^4\right] \\ &= \left(\frac{4}{3} - \frac{1}{3} C_{f_g}^{3,1}\right) \left[C_{p_{\text{LM}}}^{2,1} - (C_{p_{\text{LM}}}^{2,1})^2 - 2C_{p_{\text{LM}}}^{3,1} + 2C_{p_{\text{LM}}}^{4,1}\right].\end{aligned}$$

Summing all four together and rearranging:

$$\begin{aligned}\mathbb{E}_{r \sim \text{Unif}(\mathcal{R})}\left[C_{p_{\text{wm}}}^{2,1}\right] =& C_{p_{\text{LM}}}^{4,1} + 2C_{p_{\text{LM}}}^{3,1} - 2C_{p_{\text{LM}}}^{4,1} + \left(2 - C_{f_g}^{2,1}\right) (C_{p_{\text{LM}}}^{2,1})^2 - \left(2 - C_{f_g}^{2,1}\right) C_{p_{\text{LM}}}^{4,1} \\ &+ \left(\frac{4}{3} - \frac{1}{3} C_{f_g}^{3,1}\right) \left[C_{p_{\text{LM}}}^{2,1} - (C_{p_{\text{LM}}}^{2,1})^2 - 2C_{p_{\text{LM}}}^{3,1} + 2C_{p_{\text{LM}}}^{4,1}\right] \\ &= \left[\frac{4}{3} - \frac{1}{3} C_{f_g}^{3,1}\right] C_{p_{\text{LM}}}^{2,1} + \left[\frac{2}{3} + \frac{1}{3} C_{f_g}^{3,1} - C_{f_g}^{2,1}\right] \left(C_{p_{\text{LM}}}^{2,1}\right)^2 \\ &- \left[\frac{2}{3} - \frac{2}{3} C_{f_g}^{3,1}\right] C_{p_{\text{LM}}}^{3,1} - \left[\frac{1}{3} + \frac{2}{3} C_{f_g}^{3,1} - C_{f_g}^{2,1}\right] C_{p_{\text{LM}}}^{4,1}.\end{aligned}$$

□

K.11 Proof of Theorem 32

Proof. From Theorem 31 we have:

$$\begin{aligned}\mathbb{E}_{r \sim \text{Unif}(\mathcal{R})}\left[C_{p_{\text{wm}}}^{2,1}\right] &= \left[\frac{4}{3} - \frac{1}{3} C_{f_g}^{3,1}\right] C_{p_{\text{LM}}}^{2,1} + \left[\frac{2}{3} + \frac{1}{3} C_{f_g}^{3,1} - C_{f_g}^{2,1}\right] \left(C_{p_{\text{LM}}}^{2,1}\right)^2 \\ &- \left[\frac{2}{3} - \frac{2}{3} C_{f_g}^{3,1}\right] C_{p_{\text{LM}}}^{3,1} - \left[\frac{1}{3} + \frac{2}{3} C_{f_g}^{3,1} - C_{f_g}^{2,1}\right] C_{p_{\text{LM}}}^{4,1}.\end{aligned}$$

Noting that $\left[\frac{2}{3} - \frac{2}{3}C_{f_g}^{3,1}\right] \geq 0$, and from Lemma 44, we have $C_{p_{\mathrm{LM}}}^{3,1} \leq \frac{1}{2}C_{p_{\mathrm{LM}}}^{2,1}(1 + C_{p_{\mathrm{LM}}}^{2,1})$ (with equality iff p_{LM} is one-hot), and so:

$$
\begin{aligned}
\mathbb{E}_{r\sim\mathrm{Unif}(\mathcal{R})}\left[C_{p_{\mathrm{wm}}}^{2,1}\right] \geq& \left[\frac{4}{3} - \frac{1}{3}C_{f_g}^{3,1}\right] C_{p_{\mathrm{LM}}}^{2,1} + \left[\frac{2}{3} + \frac{1}{3}C_{f_g}^{3,1} - C_{f_g}^{2,1}\right] \left(C_{p_{\mathrm{LM}}}^{2,1}\right)^2 \\
&- \left[\frac{2}{3} - \frac{2}{3}C_{f_g}^{3,1}\right] \frac{1}{2}C_{p_{\mathrm{LM}}}^{2,1}(1 + C_{p_{\mathrm{LM}}}^{2,1}) - \left[\frac{1}{3} + \frac{2}{3}C_{f_g}^{3,1} - C_{f_g}^{2,1}\right] C_{p_{\mathrm{LM}}}^{4,1} \quad \text{(substitute)} \\
=& C_{p_{\mathrm{LM}}}^{2,1} + \left[\frac{1}{3} + \frac{2}{3}C_{f_g}^{3,1} - C_{f_g}^{2,1}\right] \left[\left(C_{p_{\mathrm{LM}}}^{2,1}\right)^2 - C_{p_{\mathrm{LM}}}^{4,1}\right]. \quad \text{(rearrange)}
\end{aligned}
$$

Note that $\left(C_{p_{\mathrm{LM}}}^{2,1}\right)^2 \geq C_{p_{\mathrm{LM}}}^{4,1}$. From Lemma 45 we have $\frac{2}{3}C_g^{3,1} \geq C_g^{2,1} - \frac{1}{3}$. It follows that $\mathbb{E}_{r\sim\mathrm{Unif}(\mathcal{R})}\left[C_{p_{\mathrm{wm}}}^{2,1}\right] \geq C_{p_{\mathrm{LM}}}^{2,1}$. □

K.12 Proof of Theorem 39

Proof. For Algorithm 5, the acceptance rate is:

$$
\begin{aligned}
&\sum_{x_{n+1}\in V} p_{\mathrm{wm}}(x_{n+1}|x_{1:n};k) \min\left(1, \frac{q_{\mathrm{wm}}(x_{n+1}|x_{1:n};k)}{p_{\mathrm{wm}}(x_{n+1}|x_{1:n};k)}\right) \\
=& \sum_{x_{n+1}\in V} \min\left(p_{\mathrm{wm}}(x_{n+1}|x_{1:n};k), q_{\mathrm{wm}}(x_{n+1}|x_{1:n};k)\right).
\end{aligned}
$$

Note that $\min(a, b)$ is concave in (a, b). Thus for two random variables a, b, we have $\mathbb{E}[\min\{a, b\}] \leq \min\left(\mathbb{E}[a], \mathbb{E}[b]\right)$ by Jensen's inequality. So taking expectation over k:

$$
\begin{aligned}
&\mathbb{E}_{k\sim\mathrm{Unif}(\mathcal{R})}\left[\text{acceptance rate}\right] \\
=& \sum_{x_{n+1}\in V} \mathbb{E}_{k\sim\mathrm{Unif}(\mathcal{R})}\left[\min\left(p_{\mathrm{wm}}(x_{n+1}|x_{1:n};k), q_{\mathrm{wm}}(x_{n+1}|x_{1:n};k)\right)\right] \\
\leq& \sum_{x_{n+1}\in V} \min\left(\mathbb{E}_{k\sim\mathrm{Unif}(\mathcal{R})}\left[p_{\mathrm{wm}}(x_{n+1}|x_{1:n};k)\right], \mathbb{E}_{k\sim\mathrm{Unif}(\mathcal{R})}\left[q_{\mathrm{wm}}(x_{n+1}|x_{1:n};k)\right]\right).
\end{aligned}
$$

Now note that:

$$
\begin{aligned}
&\mathbb{E}_{k\sim\mathrm{Unif}(\mathcal{R})}\left[p_{\mathrm{wm}}(x_{n+1}|x_{1:n};k)\right] \\
:=&\mathbb{E}_{k\sim\mathrm{Unif}(\mathcal{R})}\left(\mathbb{P}\left[\mathcal{S}(p(\cdot|x_{1:n}), f_r(x_{1:n}, k)) = x_{n+1}\right]\right) && \text{(Definition 37)} \\
=&\mathbb{E}_{r\sim\mathrm{Unif}(\mathcal{R})}\left(\mathbb{P}\left[\mathcal{S}(p(\cdot|x_{1:n}), r) = x_{n+1}\right]\right) && \text{(property of } f_r \text{, see Methods Section 5.3)} \\
=&p(x_{n+1}|x_{1:n}) && (\mathcal{S} \text{ non-distortionary})
\end{aligned}
$$

and similarly for q. Thus:

$$
\mathbb{E}_{k\sim\mathrm{Unif}(\mathcal{R})}\left[\text{acceptance rate}\right] \leq \sum_{x_{n+1}\in V} \min\left(p(x_{n+1}|x_{1:n}), q(x_{n+1}|x_{1:n})\right)
$$

$$= \sum_{x_{n+1} \in V} p(x_{n+1}|x_{1:n}) \min\left(1, \frac{q(x_{n+1}|x_{1:n})}{p(x_{n+1}|x_{1:n})}\right).$$

This is the acceptance rate for speculative sampling without watermarking (Definition 35). □

K.13 Proof of Theorem 41

Proof. There are two cases. **Case 1:** If x_{n+1} is sampled within the for loop on lines 8 to 15, we can write down the following expression for $q'(x_{n+1}|x_{1:n}; k^D, k^T)$:

$$q'(x_{n+1}|x_{1:n}; k^D, k^T) = p_{\text{wm}}(x_{n+1}|x_{1:n}; k^D) \min\left\{1, \frac{q(x_{n+1}|x_{1:n})}{p(x_{n+1}|x_{1:n})}\right\} + \left(1 - \sum_{x \in V} p_{\text{wm}}(x|x_{1:n}; k^D) \min\left\{1, \frac{q(x|x_{1:n})}{p(x|x_{1:n})}\right\}\right) (q-p)^{+}_{\text{wm}} (x_{n+1}|x_{1:n}; k^T).$$

The first term corresponds to the probability of sampling x_{n+1} from the draft model and accepting it. The second term corresponds to the probability of not accepting any token from the draft model, then sampling x_{n+1} from the rejection distribution.

Now recall that, from Definition 40:

$$\begin{aligned}
p_{\text{wm}}(x_t|x_{<t}; k^D) &:= \mathbb{P}\left[\mathcal{S}\left(p(\cdot|x_{<t}), f_r(x_{<t}, k^D)\right) = x_t\right] \\
q_{\text{wm}}(x_t|x_{<t}; k^T) &:= \mathbb{P}\left[\mathcal{S}\left(q(\cdot|x_{<t}), f_r(x_{<t}, k^T)\right) = x_t\right] \\
(q-p)^{\text{wm}}_{+}(x_t|x_{<t}; k^T) &:= \mathbb{P}\left[\mathcal{S}\left(\left[q(\cdot|x_{<t}) - p(\cdot|x_{<t})\right]_{+}, f_r(x_{<t}, k^T)\right) = x_t\right]
\end{aligned}$$

Now, taking expectation over the keys $k^D \sim \text{Unif}(\mathcal{R})$ and $k^T \sim \text{Unif}(\mathcal{R})$ is equivalent to taking expectation over the random seed $r \sim \text{Unif}(\mathcal{R})$ (see Methods Section 5.3); furthermore $\mathcal{S}$ is non-distortionary (Definition 16), so it follows that:

$$\begin{aligned}
\mathbb{E}_{k^D \sim \text{Unif}(\mathcal{R})}\left[p_{\text{wm}}(x_t|x_{<t}; k^D)\right] &= p(x_t|x_{<t}) \\
\mathbb{E}_{k^T \sim \text{Unif}(\mathcal{R})}\left[q_{\text{wm}}(x_t|x_{<t}; k^T)\right] &= q(x_t|x_{<t}) \\
\mathbb{E}_{k^T \sim \text{Unif}(\mathcal{R})}\left[(q-p)^{\text{wm}}_{+}(x_t|x_{<t}; k^T)\right] &= \left[q(x_t|x_{<t}) - p(x_t|x_{<t})\right]_{+}.
\end{aligned}$$

It follows that:

$$\mathbb{E}_{k^D \sim \text{Unif}(\mathcal{R}), k^T \sim \text{Unif}(\mathcal{R})}\left[q'(x_{n+1}|x_{1:n}; k^D, k^T)\right] = p(x_{n+1}|x_{1:n}) \min\left\{1, \frac{q(x_{n+1}|x_{1:n})}{p(x_{n+1}|x_{1:n})}\right\} + \left(1 - \sum_{x \in V} p(x|x_{1:n}) \min\left\{1, \frac{q(x|x_{1:n})}{p(x|x_{1:n})}\right\}\right) (q-p)^{+} (x_{n+1}|x_{1:n}).$$

This expression is equal to the probability distribution of the next token generated by speculative sampling, and it can be shown (see Theorem 1 proof in [5]) to be equal to the target distribution $q(x_{n+1}|x_{1:n})$.

Case 2: If x_{n+1} is sampled from $q_{\text{wm}}(\cdot|x_{1:n}; k^T)$ on line 16, then in expectation over k^T this is also $q(\cdot|x_{1:n})$. □

K.14 Proof of Theorem 42

Proof. For Algorithm 6,

$$
\begin{aligned}
&\mathbb{E}_{k^D\sim\text{Unif}(\mathcal{R}),k^T\sim\text{Unif}(\mathcal{R})}[\text{acceptance rate}] \\
=&\mathbb{E}_{k^D\sim\text{Unif}(\mathcal{R}),k^T\sim\text{Unif}(\mathcal{R})}\left[\sum_{x\in V} p_{\text{wm}}(x|x_{1:n};k^D)\min\left(1,\frac{q(x|x_{1:n})}{p(x|x_{1:n})}\right)\right] \\
=&\sum_{x\in V}\mathbb{E}_{k^D\sim\text{Unif}(\mathcal{R})}\left[p_{\text{wm}}(x|x_{1:n};k^D)\right]\min\left(1,\frac{q(x|x_{1:n})}{p(x|x_{1:n})}\right) \\
=&\sum_{x\in V} p(x|x_{1:n})\min\left(1,\frac{q(x|x_{1:n})}{p(x|x_{1:n})}\right).
\end{aligned}
\tag{K36}
$$

The last equality follows from $\mathcal{S}$ being non-distortionary (Definition 16) and the fact that taking expectation over the key is equivalent to taking expectation over the random seed (Methods Section 5.3). The expression in Equation (K36) is the acceptance rate for speculative sampling without watermarking (Definition 35). □

References

[37] Giboulot, E., Teddy, F.: Watermax: breaking the LLM watermark detectability-robustness-quality trade-off. Preprint at https://arxiv.org/abs/2403.04808 (2024).

[38] Gumbel, E.J.: Statistical Theory of Extreme Values and Some Practical Applications: a Series of Lectures vol. 33. US Government Printing Office, Washington (1954).

[39] Zhao, X., Wang, Y.-X., Li, L.: Protecting language generation models via invisible watermarking. In: International Conference on Machine Learning, pp. 42187–42199 (2023).

[40] Hopper, N., Ahn, L., Langford, J.: Provably secure steganography. IEEE Transactions on Computers **58**(5), 662–676 (2009).

[41] Zhao, X., Ananth, P.V., Li, L., Wang, Y.-X.: Provable robust watermarking for AI-generated text. In: The Twelfth International Conference on Learning Representations (2024).

[42] Wouters, B.: Optimizing watermarks for large language models. In: Forty-first International Conference on Machine Learning (2024).

[43] Fernandez, P., Chaffin, A., Tit, K., Chappelier, V., Furon, T.: Three bricks to consolidate watermarks for large language models. 2023 IEEE International Workshop on Information Forensics and Security (WIFS) (2023).

[44] Zhu, Y., Lu, S., Zheng, L., Guo, J., Zhang, W., Wang, J., Yu, Y.: Texygen: A benchmarking platform for text generation models. In: The 41st International ACM SIGIR Conference on Research & Development in Information Retrieval, pp. 1097–1100 (2018).

[45] Aaronson, S.: Watermarking of LLMs. Lecture at https://www.youtube.com/live/2Kx9jbSMZqA (2023).

[46] Glaese, A., McAleese, N., Trebacz, M., Aslanides, J., Firoiu, V., Ewalds, T., Rauh, M., Weidinger, L., Chadwick, M.J., Thacker, P., Campbell-Gillingham, L., Uesato, J., Huang, P., Comanescu, R., Yang, F., See, A., Dathathri, S., Greig, R., Chen, C., Fritz, D., Elias, J.S., Green, R., Mokrá, S., Fernando, N., Wu, B., Foley, R., Young, S., Gabriel, I., Isaac, W., Mellor, J., Hassabis, D., Kavukcuoglu, K., Hendricks, L.A., Irving, G.: Improving alignment of dialogue agents via targeted human judgements. Preprint at https://arxiv.org/abs/2209.14375 (2022).

[47] Chen, M., Tworek, J., Jun, H., Yuan, Q., Oliveira Pinto, H.P., Kaplan, J., Edwards, H., Burda, Y., Joseph, N., Brockman, G., Ray, A., Puri, R., Krueger, G., Petrov, M., Khlaaf, H., Sastry, G., Mishkin, P., Chan, B., Gray, S., Ryder, N., Pavlov, M., Power, A., Kaiser, L., Bavarian, M., Winter, C., Tillet, P., Such, F.P., Cummings, D., Plappert, M., Chantzis, F., Barnes, E., Herbert-Voss, A., Guss, W.H., Nichol, A., Paino, A., Tezak, N., Tang, J., Babuschkin, I., Balaji, S., Jain, S., Saunders, W., Hesse, C., Carr, A.N., Leike, J., Achiam, J., Misra, V., Morikawa, E., Radford, A., Knight, M., Brundage, M., Murati, M., Mayer, K., Welinder, P., McGrew, B., Amodei, D., McCandlish, S., Sutskever, I., Zaremba, W.: Evaluating large language models trained on code Preprint at https://arxiv.org/abs/2107.03374 (2021).

[48] Austin, J., Odena, A., Nye, M.I., Bosma, M., Michalewski, H., Dohan, D., Jiang, E., Cai, C.J., Terry, M., Le, Q.V., Sutton, C.: Program synthesis with large language models. Preprint at https://arxiv.org/abs/2108.07732 (2021).

[49] Hendrycks, D., Burns, C., Basart, S., Zou, A., Mazeika, M., Song, D., Steinhardt, J.: Measuring Massive Multitask Language Understanding. Proceedings of the International Conference on Learning Representations (2021).

[50] Cobbe, K., Kosaraju, V., Bavarian, M., Chen, M., Jun, H., Kaiser, L., Plappert, M., Tworek, J., Hilton, J., Nakano, R., Hesse, C., Schulman, J.: Training verifiers to solve math word problems. Preprint at https://arxiv.org/abs/2110.14168 (2021).

[51] Hendrycks, D., Burns, C., Kadavath, S., Arora, A., Basart, S., Tang, E., Song, D., Steinhardt, J.: Measuring mathematical problem solving with the MATH dataset.

Proceedings of the Neural Information Processing Systems Track on Datasets and Benchmarks, vol. 1 (2021).

[52] Zhong, W., Cui, R., Guo, Y., Liang, Y., Lu, S., Wang, Y., Saied, A., Chen, W., Duan, N.: AGIEval: A Human-Centric Benchmark for Evaluating Foundation Models. Preprint at https://arxiv.org/abs/2304.06364 (2023).

[53] Suzgun, M., Scales, N., Schärli, N., Gehrmann, S., Tay, Y., Chung, H.W., Chowdhery, A., Le, Q., Chi, E., Zhou, D., Wei, J.: Challenging BIG-Bench tasks and whether chain-of-thought can solve them. In: Findings of the Association for Computational Linguistics: ACL 2023, pp. 13003–13051. Association for Computational Linguistics, Toronto, Canada (2023).

[54] Jelinek, F., Mercer, R.L., Bahl, L.R., Baker, J.K.: Perplexity—a measure of the difficulty of speech recognition tasks. The Journal of the Acoustical Society of America **62**(S1), 63–63 (1977).

[55] Almazrouei, E., Alobeidli, H., Alshamsi, A., Cappelli, A., Cojocaru, R., Debbah, M., Goffinet, E., Heslow, D., Launay, J., Malartic, Q., Noune, B., Pannier, B., Penedo, G.: Falcon-40B: an open large language model with state-of-the-art performance. Preprint at https://arxiv.org/abs/2311.16867 (2023).

[56] Hasan, T., Bhattacharjee, A., Islam, M.S., Mubasshir, K., Li, Y.-F., Kang, Y.-B., Rahman, M.S., Shahriyar, R.: XL-Sum: Large-scale multilingual abstractive summarization for 44 languages. In: Findings of the Association for Computational Linguistics: ACL-IJCNLP 2021, pp. 4693–4703. Association for Computational Linguistics, (2021).

[57] Shaffer, J.P.: Multiple hypothesis testing. Annual review of psychology **46**(1), 561–584 (1995).
